# Direct Synthesis of 2-(4-Hydroxyphenoxy)benzamide Derivatives from 2-Aryloxybenzamide via PhIO-Mediated Oxidation Reaction

**DOI:** 10.3390/molecules29246048

**Published:** 2024-12-22

**Authors:** Zhenhua Shang, Dechen Jiao, Haoran Cheng, Daowei Huang

**Affiliations:** 1 School of Chemical and Pharmaceutical Engineering, Hebei University of Science and Technology, Shijiazhuang 050018, China; dechenjiao1999@163.com (D.J.); chenghr1114@163.com (H.C.); 2 Hebei Research Center of Pharmaceutical and Chemical Engineering, Shijiazhuang 050018, China; 3 State Key Laboratory Breeding Base-Hebei Province Key Laboratory of Molecular Chemistry for Drug, Shijiazhuang 050200, China

**Keywords:** 2-(4-hydroxyphenoxy)benzamide, 2-aryloxybenzamide, PhIO, hypervalent iodine

## Abstract

The 2-(4-hydroxyphenoxy)benzamide scaffold is frequently found in a variety of bioactive compounds, displaying a broad spectrum of properties, such as antibacterial and antitumor effects. In this study, we developed a new method for synthesizing 2-(4-hydroxyphenoxy)benzamide derivatives from 2-aryloxybenzamide via a PhIO-mediated oxidation reaction. The optimal reaction conditions were established as follows: TFA was used as the solvent, PhIO served as the oxidant with a substrate-to-oxidant ratio of 1:2, and the reaction was conducted at room temperature. This method, characterized by mild reaction conditions, broad applicability, and a metal-free nature, considerably improves the accessibility of 2-(4-hydroxyphenoxy)benzamide derivatives, which have been challenging to prepare using previously reported methods.

## 1. Introduction

**Figure 1:**
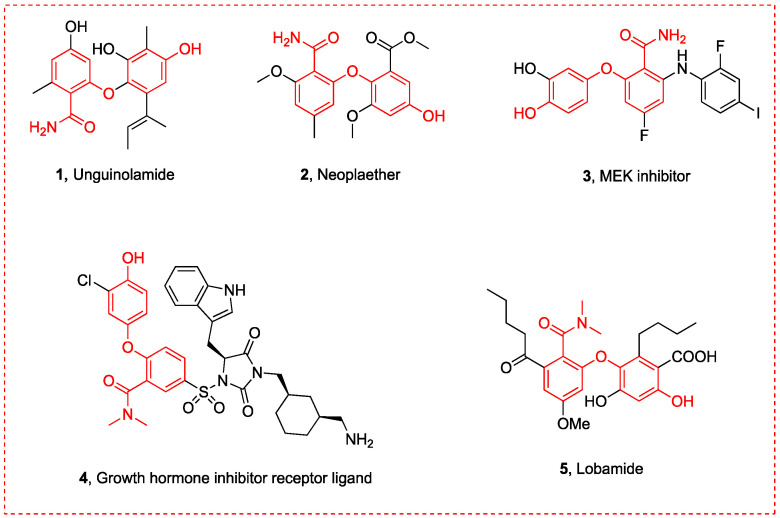
Reported bioactive compounds featuring 2-(4-hydroxyphenoxy)benzamide scaffold.

A 2-(4-hydroxyphenoxy)benzamide scaffold is commonly present in various bioactive compounds, exhibiting a wide range of properties [1,2,3], including antibacterial and antitumor effects (Figure 1). Unguinolamide (**1**), a semi-synthetic amide compound, demonstrates broad antibacterial activity against Gram-positive bacteria [4]. Neoplaether (**2**), an active substance derived from fungal cultures, exhibits remarkable cytotoxic activity against nasopharyngeal epithelial tumors and antifungal activity against Candida albicans [5]. Compound **3** is a selective MEK inhibitor, known for its high antitumor activity [6,7], while compound **4** functions as a ligand used for growth hormone receptor inhibition [8]. Lobamide (**5**) acts as a protein tyrosine inhibitor and shows potential therapeutic effects for diabetes and obesity [9]. Given the significant biological activity of 2-(4-hydroxyphenoxy)benzamide derivatives [1,2,3], developing an effective synthetic method is essential. However, no such method has been reported to date. Consequently, the development of new synthetic approaches for the 2-(4-hydroxyphenoxy)benzamide derivative has recently garnered significant attention in the fields of organic and medicinal chemistry.

**Figure 2:**
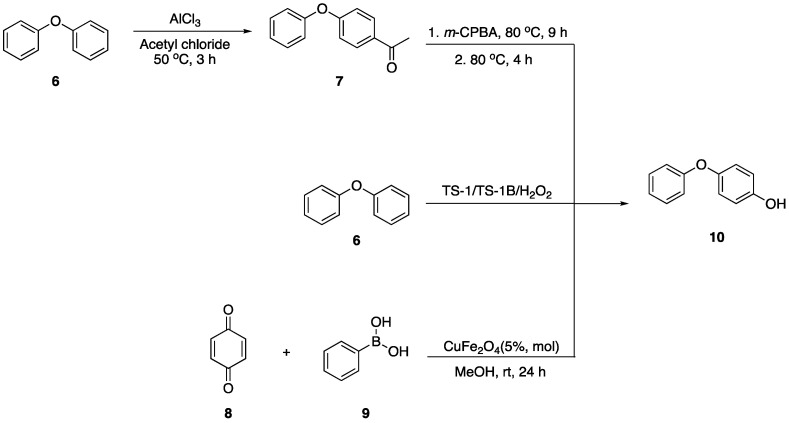
The reported synthetic method of 4-phenoxyphenol.

To develop new methods for synthesizing the 2-(4-hydroxyphenoxy)benzamide derivatives, we initially investigated the preparation of the 4-phenoxyphenol scaffold (**10**), see Figure 2 below. The literature outlines the utilization of diphenyl ether (**6**) as a starting material. Initially, diphenyl ether is converted to 4-phenoxyacetophenone (**7**) via a Friedel–Crafts acylation reaction. This intermediate then undergoes a Baeyer–Villiger rearrangement in the presence of a peracid, forming an ester. The subsequent hydrolysis of the ester produces 4-phenoxyphenol (**8**) [10]. Bianchi et al. reported a catalytic method utilizing titanium silicate TS-1 or TS-1B with hydrogen peroxide to oxidize diphenyl ether to 4-phenoxyphenol, achieving yields of 78–82% [11]. Wang et al. proposed an approach that employs diphenyl ether compounds as raw materials in conjunction with boronic esters, organic bases, brominating reagents, and oxygen [12]. This approach facilitates the synthesis of 4-hydroxyphenol compounds under 390 nm LED light irradiation for 12–72 h, yielding moderate yields. Song et al. employed substituted arylboronic acids and p-benzoquinone derivatives as raw materials to selectively produce a range of 4-phenoxyphenol compounds with varying substituents via a copper catalyst process [13].

Introducing a phenolic hydroxyl group onto a benzene ring entails several significant challenges. Firstly, the selectivity of substitution reactions can yield multiple products, complicating the isolation and purification of the target compound. Additionally, these reactions often require harsh conditions—such as elevated temperatures or specific catalysts. The relatively low electrophilicity of benzene further complicates the process, necessitating the use of stronger electrophiles to achieve satisfactory reactivity. Furthermore, the synthetic pathway typically involves multiple steps, including protection and subsequent transformation, which adds to the overall complexity of the synthesis. Together, these factors render the introduction of a phenolic hydroxyl group onto a benzene ring a particularly challenging endeavor in the realm of synthetic organic chemistry.

**Figure 3:**
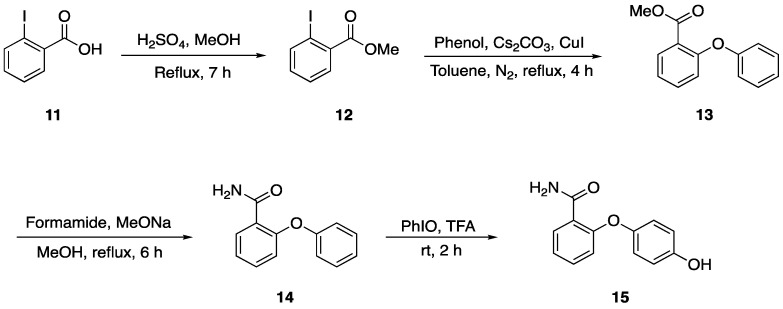
A new synthetic method of 2-(4-hydroxyphenoxy) benzamide (**15**).

Our group has been conducting studies on hypervalent iodine [14,15,16], and in view of our long-standing interests in developing new strategies for synthesizing the 2-(4-hydroxyphenoxy)benzamide derivatives, we present a new synthetic method that combines esterification, Ullmann coupling, ammonification, and oxidation reactions. The detailed synthesis pathway is depicted in Figure 3, where compound **12** is generated through the esterification of ortho-iodobenzoic acid (**11**) [17]. This is followed by the formation of intermediate **13** via the Ullmann reaction [18]. An amination reaction then yields compound **14** [19], and the final target product **15** is obtained from intermediate **14** through an oxidation process. This method is environmentally friendly, free from heavy metals, and enables the efficient synthesis of the 2-(4-hydroxyphenoxy)benzamide derivatives with high yields.

## 2. Results and Discussion

**Figure 4:**
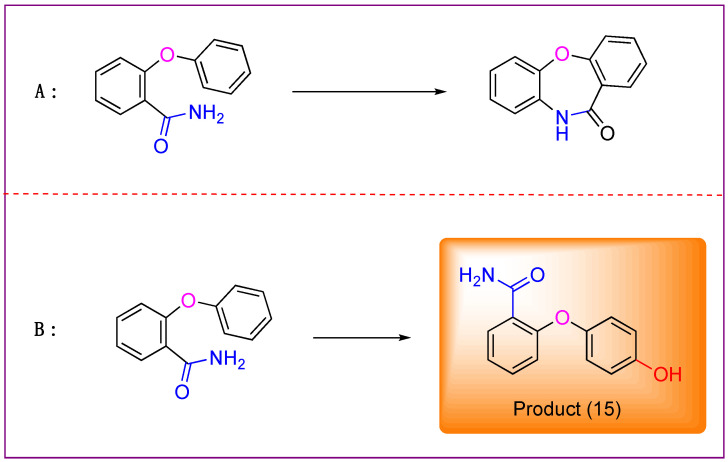
(**A**) The expected product based on our previous work, which failed; (**B**) The newly discovered product from our previous work.

Drawing on our group’s previous experience in synthesizing dibenzodiazepinones using hypervalent iodine reagents (unpublished), we initially aimed to synthesize the target dibenzoxadiazepine derivatives through a Hofmann rearrangement mediated by hypervalent iodine reagents, followed by the electrophilic cyclization of o-aryloxybenzamide compounds as substrates. However, to our surprise, the predominant product was identified as 2-(4-hydroxyphenoxy) benzamide, see Figure 4 below. These unexpected results prompted a comprehensive investigation into the reaction mechanism and outcomes.

Our previous study commenced with the screening of 2-phenoxybenzamide (**14**) as a model substrate to assess the feasibility of an oxidation reaction for the synthesis of dibenzoxadiazepine derivatives. Initially, the reaction was conducted in an ice bath using PhIO (iodobenzene diacetate) as the oxidant, KOH as the base, and MeOH as the solvent. However, this attempt predominantly yielded (2-phenoxyphenyl) methyl formate. To optimize the reaction, we tested various solvents. Despite experimenting with several commonly used solvents, the results remained unsatisfactory. When tetrahydrofuran (THF) was utilized as the solvent, the resulting product was identified as 4-(2-carbamoylphenoxy)-*N*-(2-phenoxyphenyl)benzamide. When PIDA (phenyl iodinate) was utilized as the oxidant, THF as the solvent, and KOH as the base, the results remained suboptimal. Drawing insights from the literature, we investigated the effects of fluorinated solvents on hypervalent iodine-mediated reactions. We tested trifluoroethanol (TFE), hexafluoroisopropanol (HFIP), and trifluoroacetic acid (TFA). Remarkably, the use of TFA as the solvent facilitated the formation of a new compound, making a breakthrough in our synthetic efforts.

**Figure 5:**
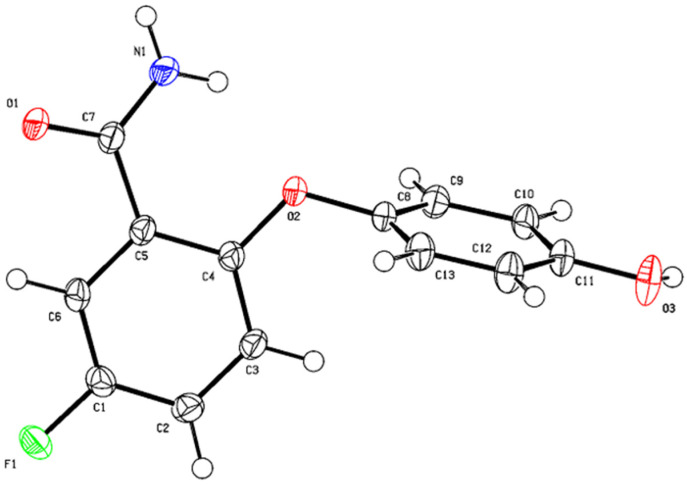
Single-crystal of 5-fluoro-2-phenoxybenzamide.

Subsequently, we performed single-crystal growth experiments on various oxidation products. Notably, the single crystals of the oxidation product derived from 5-fluoro-2-phenoxybenzamide were successfully obtained. Further, the single-crystal diffraction analysis of these crystals provided definitive structural elucidation of the product, validating our synthetic pathway and contributing to a deeper understanding of the reaction mechanism. The diffraction data are presented in Figure 5 (diffraction conditions: a Rigaku Saturn 944+ instrument, with the crystal maintained at a temperature of 113.15 K for data collection).

We conducted an optimization study by systematically varying parameters such as the substrate-to-reagent ratio, temperature, and hypervalent reagents, which led to the identification of the optimal reaction conditions. The optimization of oxidation conditions is summarized in Table 1. Initially, we conducted a blank experiment (entry 1) in the absence of a hypervalent iodine reagent, with the reaction temperature increasing from room temperature to reflux. However, the reaction did not proceed under these conditions. We subsequently varied the ratios of the starting materials. As presented in entries 2–5 of Table 1, at a substrate–PhIO ratio of 1:1, the reaction was incomplete, consistently leaving a small amount of unreacted starting materials. Adjusting the ratio to 1:1.2 or 1:1.5 resulted in increased reaction times but without significant improvement in yield. Nevertheless, at a ratio of 1:2.5, the formation of by-products increased markedly, likely due to the high concentration of hypervalent iodine, which substantially decreased the conversion rate of the target product, yielding only 28.2%. The highest yield of 72.9% was achieved at a substrate–PhIO ratio of 1:2. As illustrated in Table 1 (entry 4, 6, and 7), reaction temperature influenced the reaction time and yield. As the temperature decreased, the reaction time increased. Meanwhile, the highest yield was obtained at room temperature, where the reaction rate was optimal. Furthermore, increasing the temperature significantly shortened the reaction time but also led to greatly increased by-product formation, notably reducing the yield.

Alternative hypervalent iodine reagents were also evaluated (Table 1, entry 8–10). PIDA and PIFA (2,3-difluoroacetoxyiodobenzene) produced the target product but with low yields. Koser’s reagent yielded a mixture of products, preventing the isolation of the target product. Among solvents, attempts to use protonic solvents other than TFA yielded poor reaction outcomes (Table 1, entry 11–13). Only the solvent condition of HFIP, in combination with PIFA as the hypervalent iodine reagent, led to a moderate yield of 58.2% (Table 1, entry 14). However, polar nonprotic solvents such as THF, chloroform, 1,4-dioxane, and acetonitrile, in conjunction with the aforementioned hypervalent iodine reagents, failed to yield the target product. Based on these findings, the optimal reaction conditions were determined to be TFA as the solvent and PhIO as the oxidant at a substrate–oxidant ratio of 1:2 at room temperature.

Following the successful synthesis of various substituted 2-(hydroxyphenoxy) benzamide compounds, we aimed to broaden the substrate range by employing substituted primary and secondary amines. These amines were used in amine–ester exchange reaction with ortho-phenoxybenzoic acid methyl ester to produce new substrates. Using the optimized oxidation reaction conditions established in our previous screening, we evaluated the applicability of this method to an expanded substrate range.

A series of substituted 2-(hydroxyphenoxy) benzamide derivatives were synthesized under the optimal conditions (Table 2, entry 5) to investigate the scope and generality of the proposed new synthetic method. As shown in Table 2, substrates with various substituents (R^1^, R^2^) on the benzene ring produced yields ranging from moderate to excellent, and the reaction time was about 2 h. Notably, electron-withdrawing groups (R^1^) generally resulted in slightly higher yields than electron-donating groups (R^1^) (Table 3, entries 1–5). Substituents at position R^2^, regardless of whether they were electron donating or electron withdrawing, consistently enhanced yields (Table 2, entries 1–4). When R^1^ was hydrogen and R^2^ was an electron-donating group, yields were slightly higher (Table 2, entries 1–4). For R^1^ = CH_3_, yields were comparable when R^2^ was either hydrogen or methyl (Table 2, entries 5–6). When R^1^ was chlorine, the yield was higher for R^2^ = methyl than that for R^2^ = phenyl (Table 2, entries 7–8). For R^1^ = fluorine, the best yield was observed with R^2^ = hydrogen, followed by R^2^ = methyl and R^2^ = phenyl (Table 2, entries 9–11).

To further investigate the scope and generality of the proposed new synthetic method, a series of substituted amines were synthesized under the optimal conditions (Table 3). The yield and reaction time varied with different substituents. The introduction of substituents on the amines resulted in a decrease in the oxidation yields (entries 1–7).

We further investigated a range of substrates while maintaining the core structure of diphenyl ether skeleton (Table 4). However, the oxidation of methyl 2-phenoxybenzoate did not proceed (entry 1). Similarly, no desired oxidation products were obtained when using other substrates, including dophenyl ether (entry 2), 1,4-diphenoxybenzene (entry 3), 4-phenoxybenzophenone (entry 4), dibenzofuran (entry 6), and 2-iodobenzamide (entry 7). Notably, when oxazanthrene was used as the substrate, oxidation led to the formation of oxazanthrenone in a yield of 76.8% (entry 5). We also investigated the oxidation of 2-(4-methylphenoxy)benzamide (entry 8) as the substrates; however, the reaction also did not proceed, which indicated that no reaction occurred when a group was present in the para position relative to the ether bond (entry 8). In summary, the amide structure and the diphenyl ether framework are essential for the oxidation reaction.

**Figure 6:**
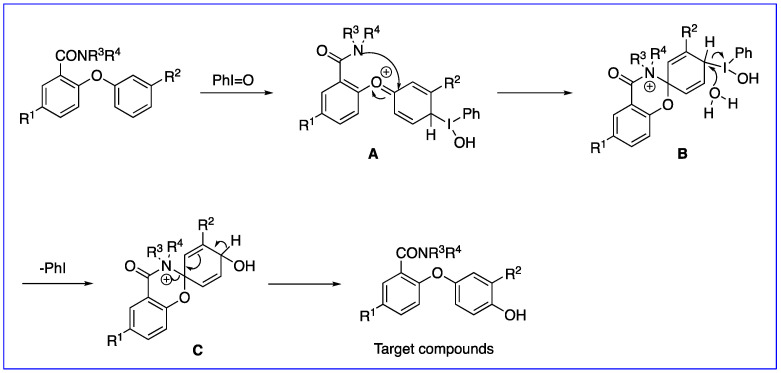
The proposed mechanism.

We propose a plausible mechanism for the PhIO-mediated oxidation reaction (Figure 6). The substrate undergoes oxidation in the presence of PhI=O, leading to the formation of the oxonium intermediate A. The six-membered intermediate B is produced by an intramolecular nuclephilic attack from the amide’s nitrogen. Subsequently, intermediate B undergoes elimination, releasing iodobenzene and forming intermediate C. The target product is ultimately generated through deprotonation, restoring the amide group to its original state.

## 3. Experimental Section

^1^H-NMR and ^13^C-NMR spectra were recorded on a 500 MHz instrument (125 MHz for ^13^C NMR, Bruker Bioscience, Billerica, MA, USA) at 25 °C. Chemical shift values were reported in ppm, and with tetramethylsilane (TMS) as the internal reference, set at 0.00 ppm. The multiplicity of the signals is denoted as follows: s (singlet); d (doublet), t (triplet), q (quadruplet), qui (quintuplet), m (multiplet), dd (doublet of doublets), and dt (doublet of triplets). Coupling constants (J) are given in hertz (Hz). High-resolution mass spectra (HRMS) data were acquired on a Q-TOF microspectrometer (Thermo Fisher Scientific, Waltham, MA, USA). Melting points were determined using a standard micro-melting point apparatus (SGWX-4, Shenzhen, China) and were reported without correction. Flash chromatography was performed using silica gel (200–300 mesh) as the stationary phase, with a mobile phase consisting of a mixture of methanol (MeOH), ethyl acetate (EA), and petroleum ether (PE). Thin-layer chromatography (TLC) was carried out on glass-backed plates pre-coated with silica gel 60 GF254, developed with standard visualization agents, and the spots were visualized under ultraviolet (UV) light. All reagents and solvents were purchased from commercial sources and were used without further purification. Various substituted 2-(methyl(phenyl)amino)benzoic acids and 2-(methyl(phenyl)amino)benzamide were synthesized in-house. All ^1^H NMR, ^13^C NMR, and HR-MS spectra are available in Supplementary Materials.

### 3.1. Preparation of Methyl 2-Iodobenzoate *(**12**)*

The intermediate methyl 2-iodobenzoate (**12**) was prepared according to the literature [20]. To the solution of 2-iodobenzoic acid (15 g, 0.06 mol) in methanol (200 mL) was added concentrated sulfuric acid (9 mL), and the reaction mixture was stirred at room temperature under a nitrogen atmosphere for 7 h. The mixture was then cooled to room temperature, and part of the organic solvent was removed by evaporation. Acetate ester (80 mL) was added, and the organic phase was sequentially washed with 10% aqueous Na_2_CO_3_ (30 mL × 3), 1 M hydrochloric (30 mL × 3), and water (15 mL × 3). The organic phase was dried over anhydrous MgSO_4_ and concentrated to yield methyl 2-iodobenzoate (**12**), which was used directly for the next step without further purification (15.26 g, 96.3%).

### 3.2. Preparation of Methyl 2-Phenoxybenzoate *(**13**)*

To the solution of methyl 2-iodobenzoate (12, 4.0 g, 15.26 mmol) and phenol (1.72 g, 18.32 mmol) in toluene (40 mL), Cs_2_CO_3_ (7.46 g, 22.90 mmol) and copper(I) iodine (2.91 g, 15.26 mmol) were added; the reaction mixture was stirred at reflux under nitrogen atmosphere for 4 h. The mixture was then cooled to room temperature, filtered through celite, and washed an with acetate ester (15 mL × 3). The organic phase was sequentially washed with saturated saline (15 mL × 3) and water (15 mL × 3), dried over anhydrous MgSO_4_, filtered, and concentrated to yield the crude product, which was purified by flash chromatography (PE:EA = 50:1) to obtain methyl 2-phenoxybenzoate (13, 2.90 g, 83.3%). ^1^H NMR (500 MHz, DMSO-*d*_6_) δ 7.86 (dd, *J* = 7.8, 1.8 Hz, 1H), 7.61 (ddd, *J* = 8.2, 7.4, 1.8 Hz, 1H), 7.42–7.34 (m, 2H), 7.30 (td, *J* = 7.6, 1.1 Hz, 1H), 7.13 (tt, *J* = 7.3, 1.1 Hz, 1H), 7.05 (dd, *J* = 8.3, 1.1 Hz, 1H), 6.98–6.91 (m, 2H), 3.73 (s, 3H). ^13^C NMR (126 MHz, DMSO-*d*_6_) δ 165.90, 157.72, 155.54, 134.52, 131.85, 130.44, 124.50, 123.63, 123.56, 121.40, 118.25, 52.56.

### 3.3. General Procedure for Preparation of Intermediates ***14a**–**14r***

To the solution of methyl 2-phenoxybenzoate (**13**, 1.0 g, 4.38 mmol) and varied amines (1.0 mL) in methanol (40 mL), sodium methoxide (0.47 g, 8.70 mmol) was added. The reaction solution was stirred at reflux under a nitrogen atmosphere for 6 h. The mixture was then cooled to room temperature, and water (35 mL) was added. The precipitate solid was filtered, washed with water (10 mL × 3), and dried to yield the desired product as a white solid.

2-Phenoxybenzamide (**14a**): 91.3%; m.p. 122–126 °C; ^1^H NMR (500 MHz, DMSO-*d*_6_) δ 7.76 (dd, *J =* 7.7, 1.8 Hz, 1H), 7.65 (s, 1H), 7.57 (s, 1H), 7.50–7.38 (m, 3H), 7.23 (td, *J* = 7.5, 1.1 Hz, 1H), 7.18 (t, *J* = 7.4 Hz, 1H), 7.08–7.03 (m, 2H), 6.90 (dd, *J* = 8.2, 1.1 Hz, 1H); ^13^C NMR (126 MHz, DMSO-*d*_6_) δ 167.11, 156.84, 154.37, 132.40, 130.81, 130.51, 127.96, 124.20, 124.03, 119.48, 119.25.

2-(m-Tolyloxy)benzamide (**14b**): 84.0%; m.p. 100–102 °C; ^1^H NMR (500 MHz, DMSO-*d*_6_) δ 7.76 (dd, *J* = 7.7, 1.8 Hz, 1H), 7.61 (s, 1H), 7.56 (s, 1H), 7.46 (ddd, *J* = 8.7, 7.4, 1.8 Hz, 1H), 7.29 (t, *J* = 7.8 Hz, 1H), 7.22 (td, *J* = 7.5, 1.1 Hz, 1H), 6.99 (d, *J* = 7.5 Hz, 1H), 6.92–6.86 (m, 2H), 6.83 (dd, *J* = 8.1, 2.5 Hz, 1H), 2.31 (s, 3H). ^13^C NMR (126 MHz, DMSO-*d*_6_) δ 167.05, 156.78, 154.46, 140.23, 132.41, 130.82, 130.22, 127.78, 124.94, 123.93, 119.76, 119.47, 116.28, 21.43.

2-(3-Bromophenoxy)benzamide (**14c**): 90.8%; m.p. 105–107 °C; ^1^H NMR (500 MHz, DMSO-*d*_6_) δ 7.72 (dd, *J* = 7.7, 1.8 Hz, 1H), 7.68 (s, 1H), 7.55–7.46 (m, 2H), 7.40–7.33 (m, 2H), 7.29 (td, *J* = 7.5, 1.1 Hz, 1H), 7.24–7.20 (m, 1H), 7.06–6.97 (m, 2H). ^13^C NMR (126 MHz, DMSO-*d*_6_) δ 167.13, 158.31, 153.19, 132.43, 132.13, 130.67, 128.95, 126.69, 124.91, 122.60, 121.56, 120.46, 117.77.

2-(3-Chlorophenoxy)benzamide (**14d**): 74.5%; m.p. 93–95 °C; ^1^H NMR (500 MHz, DMSO-*d*_6_) δ 7.72 (dd, *J* = 7.7, 1.8 Hz, 1H), 7.68 (s, 1H), 7.55–7.47 (m, 2H), 7.42 (t, *J* = 8.1 Hz, 1H), 7.30 (td, *J* = 7.5, 1.1 Hz, 1H), 7.21 (ddd, *J* = 8.0, 2.0, 0.9 Hz, 1H), 7.09 (t, *J* = 2.2 Hz, 1H), 7.02 (dd, *J* = 8.2, 1.1 Hz, 1H), 6.98 (ddd, *J* = 8.3, 2.4, 0.9 Hz, 1H). ^13^C NMR (126 MHz, DMSO-*d*_6_) δ 167.13, 158.30, 153.17, 134.36, 132.42, 131.82, 130.67, 128.97, 124.92, 123.78, 120.49, 118.74, 117.36.

5-Methyl-2-phenoxybenzamide (**14e**): 72.2%; m.p. 99–102 °C; ^1^H NMR (500 MHz, DMSO-*d*_6_) δ 7.60–7.55 (m, 2H), 7.51 (s, 1H), 7.43–7.34 (m, 2H), 7.28 (ddd, *J* = 8.4, 2.4, 0.8 Hz, 1H), 7.14 (tt, *J* = 7.4, 1.1 Hz, 1H), 7.03–6.97 (m, 2H), 6.84 (d, *J* = 8.3 Hz, 1H), 2.34 (s, 3H). ^13^C NMR (126 MHz, DMSO-*d*_6_) δ 167.06, 157.39, 151.91, 133.42, 132.88, 131.04, 130.41, 127.84, 123.79, 120.10, 118.67, 20.67.

5-Methyl-2-(m-tolyloxy)benzamide (**14f**): 88.3%; m.p. 100–103 °C; ^1^H NMR (500 MHz, DMSO-*d*_6_) δ 7.58 (d, *J* = 2.3 Hz, 1H), 7.55 (s, 1H), 7.52 (s, 1H), 7.30–7.21 (m, 2H), 6.95 (d, *J* = 7.5 Hz, 1H), 6.86–6.81 (m, 2H), 6.79 (dd, *J* = 8.1, 2.5 Hz, 1H), 2.33 (s, 3H), 2.29 (s, 3H). ^13^C NMR (126 MHz, DMSO-*d*_6_) δ 167.02, 157.33, 152.00, 140.11, 133.33, 132.92, 131.06, 130.13, 127.65, 124.54, 120.11, 119.16, 115.72, 21.44, 20.67.

5-Chloro-2-(m-tolyloxy)benzamide (**14g**): 84.8%; m.p. 126–128 °C; ^1^H NMR (500 MHz, DMSO-*d*_6_) δ 7.75–7.67 (m, 3H), 7.50 (dd, *J* = 8.8, 2.8 Hz, 1H), 7.30 (t, *J* = 7.8 Hz, 1H), 7.01 (ddt, *J* = 7.6, 1.7, 0.9 Hz, 1H), 6.94–6.88 (m, 2H), 6.86 (dd, *J* = 8.1, 2.5 Hz, 1H), 2.31 (s, 3H). ^13^C NMR (126 MHz, DMSO-*d*_6_) δ 165.79, 156.48, 153.41, 140.37, 131.98, 130.30, 130.03, 129.55, 127.67, 125.30, 121.26, 119.92, 116.46, 21.41.

2-([1,1′-Biphenyl]-3-yloxy)-5-chlorobenzamide (**14h**): 55.2%; m.p. 130–133 °C; ^1^H NMR (500 MHz, DMSO-*d*_6_) δ 7.82 (s, 1H), 7.72 (s, 2H), 7.67 (d, *J* = 7.7 Hz, 2H), 7.49 (q, *J* = 8.4, 7.6 Hz, 5H), 7.39 (d, *J* = 6.5 Hz, 2H), 7.06 (d, *J* = 6.5 Hz, 1H), 7.01 (d, *J* = 8.8 Hz, 1H). ^13^C NMR (126 MHz, DMSO-*d*_6_) δ 165.90, 157.14, 153.21, 142.76, 139.75, 131.99, 131.11, 130.04, 129.89, 129.49, 128.39, 127.87, 127.27, 122.88, 121.42, 118.32, 117.63.

5-Fluoro-2-phenoxybenzamide (**14i**): 76.0%; m.p. 122–124 °C; ^1^H NMR (500 MHz, DMSO-*d*_6_) δ 7.73 (s, 1H), 7.68 (s, 1H), 7.52 (dd, *J* = 9.0, 3.3 Hz, 1H), 7.44–7.37 (m, 2H), 7.33 (ddd, *J* = 9.0, 7.9, 3.3 Hz, 1H), 7.19–7.12 (m, 1H), 7.05–7.02 (m, 1H), 7.02–6.97 (m, 2H). ^13^C NMR (126 MHz, DMSO-*d*_6_) δ 165.84, 159.25, 157.32, 150.22, 150.20, 130.49, 130.02, 124.04, 122.22, 122.15, 119.15, 118.97, 118.68, 116.91, 116.71.

5-Fluoro-2-(m-tolyloxy)benzamide (**14j**): 84.2%; m.p. 124–127 °C; ^1^H NMR (500 MHz, DMSO-*d*_6_) δ 7.69 (d, *J* = 11.5 Hz, 2H), 7.52 (dd, *J* = 9.0, 3.3 Hz, 1H), 7.36–7.22 (m, 2H), 7.02–6.91 (m, 2H), 6.86 (t, *J* = 2.1 Hz, 1H), 6.80 (dd, *J* = 8.2, 2.5 Hz, 1H), 2.30 (s, 3H). ^13^C NMR (126 MHz, DMSO-*d*_6_) δ 165.80, 159.20, 157.28, 157.25, 150.32, 150.30, 140.23, 130.20, 129.83, 129.77, 124.80, 122.18, 122.12, 119.20, 119.17, 118.99, 116.88, 116.69, 115.72, 21.42.

2-([1,1′-Biphenyl]-3-yloxy)-5-fluorobenzamide (**14k**): 53.1%; m.p. 164–166 °C; ^1^H NMR (500 MHz, DMSO-*d*_6_) δ 7.79 (s, 1H), 7.70 (s, 1H), 7.68–7.62 (m, 2H), 7.53 (dd, *J* = 8.9, 3.2 Hz, 1H), 7.51–7.45 (m, 4H), 7.42–7.31 (m, 3H), 7.09 (dd, *J* = 9.0, 4.5 Hz, 1H), 7.03–6.98 (m, 1H). ^13^C NMR (126 MHz, DMSO-*d*_6_) δ 165.93, 159.32, 157.90, 157.41, 150.08, 142.67, 139.86, 131.00, 130.16, 129.49, 128.36, 127.25, 122.39, 119.17, 118.98, 117.54, 116.92, 116.72.

*N*-Butyl-2-phenoxybenzamide (**14l**): 85.6%; m.p. 158–160 °C; ^1^H NMR (500 MHz, DMSO-*d*_6_) δ 8.17 (t, *J* = 5.9 Hz, 1H), 7.66 (dd, *J* = 7.6, 1.8 Hz, 1H), 7.50–7.43 (m, 1H), 7.43–7.35 (m, 2H), 7.24 (td, *J* = 7.5, 1.1 Hz, 1H), 7.15 (tt, *J* = 7.4, 1.1 Hz, 1H), 7.05–6.98 (m, 2H), 6.94 (dd, *J* = 8.2, 1.1 Hz, 1H), 3.20 (q, *J* = 6.6 Hz, 2H), 1.42–1.36 (m, 2H), 1.28–1.20 (m, 2H), 0.83 (t, *J* = 7.3 Hz, 3H). ^13^C NMR (126 MHz, DMSO-*d*_6_) δ 165.49, 157.12, 153.86, 131.98, 130.46, 130.40, 129.02, 124.19, 123.94, 119.83, 118.86, 39.12, 31.60, 19.97, 14.16.

*N*-Methyl-2-phenoxy-*N*-phenylbenzamide (**14m**): 85.6%; m.p. 161–163 °C; ^1^H NMR (500 MHz, DMSO-*d*_6_) δ 7.38 (t, *J* = 7.7 Hz, 3H), 7.25–7.14 (m, 7H), 7.00 (t, *J* = 7.6 Hz, 1H), 6.78 (d, *J* = 5.0 Hz, 2H), 6.55 (d, *J* = 8.4 Hz, 1H), 3.36 (s, 3H). ^13^C NMR (126 MHz, DMSO-*d*_6_) δ 167.50, 156.23, 152.90, 143.94, 130.72, 130.42, 129.81, 129.25, 127.38, 127.12, 124.34, 123.17, 119.60, 117.42, 37.15.

*N*-Cyclohexyl-2-phenoxybenzamide (**14n**): 50.0%; m.p. 97–99 °C; ^1^H NMR (500 MHz, DMSO-d6) δ 8.00 (d, *J* = 8.0 Hz, 1H), 7.64 (dd, *J* = 7.6, 1.8 Hz, 1H), 7.47 (td, *J* = 7.7, 1.8 Hz, 1H), 7.43–7.35 (m, 2H), 7.25 (td, *J* = 7.5, 1.1 Hz, 1H), 7.17–7.10 (m, 1H), 7.03–6.98 (m, 2H), 6.97 (dd, *J* = 8.2, 1.1 Hz, 1H), 3.71–3.64 (m, 1H), 1.71–1.61 (m, 4H), 1.53 (dt, *J* = 13.2, 3.8 Hz, 1H), 1.31–1.05 (m, 5H). ^13^C NMR (126 MHz, DMSO-*d*_6_) δ 164.61, 157.24, 153.62, 131.98, 130.45, 130.41, 129.30, 124.36, 123.82, 120.18, 118.53, 48.28, 32.58, 25.67, 24.89.

(2-Phenoxyphenyl)(pyrrolidin-1-yl)methanone (**14o**): 88.6%; m.p. 94–97 °C; ^1^H NMR (500 MHz, DMSO-*d*_6_) δ 7.44–7.35 (m, 4H), 7.21 (td, *J* = 7.4, 1.1 Hz, 1H), 7.16 (tt, *J* = 7.4, 1.1 Hz, 1H), 7.04–6.98 (m, 2H), 6.94 (dd, *J* = 8.3, 1.0 Hz, 1H), 3.36 (t, *J* = 6.8 Hz, 2H), 3.25 (t, *J* = 6.5 Hz, 2H), 1.87–1.72 (m, 4H). ^13^C NMR (126 MHz, DMSO-*d*_6_) δ 165.89, 156.85, 152.90, 130.96, 130.46, 130.37, 128.79, 124.21, 124.17, 119.22, 47.91, 45.63, 25.93, 24.46.

(2-Phenoxyphenyl)(piperidin-1-yl)methanone (**14p**): 52.4%; m.p. 91–93 °C; ^1^H NMR (500 MHz, DMSO-*d*_6_) δ 7.40 (ddd, *J* = 10.3, 5.7, 2.1 Hz, 3H), 7.34 (dd, *J* = 7.6, 1.7 Hz, 1H), 7.22 (td, *J* = 7.4, 1.1 Hz, 1H), 7.16 (t, *J* = 7.4 Hz, 1H), 7.04–6.98 (m, 2H), 6.91 (dd, *J* = 8.3, 1.0 Hz, 1H), 3.54 (td, *J* = 7.6, 5.0 Hz, 2H), 3.22 (t, *J* = 5.5 Hz, 2H), 1.61–1.40 (m, 6H). ^13^C NMR (126 MHz, DMSO-d6) δ 165.89, 156.85, 152.75, 130.77, 130.52, 129.34, 128.73, 124.27, 124.14, 119.10, 118.89, 47.89, 42.18, 26.41, 25.77, 24.46.

*N*-Butyl-5-methyl-2-(m-tolyloxy)benzamide (**14q**): 62.4%; m.p. 89–92 °C; ^1^H NMR (500 MHz, DMSO-*d*_6_) δ 8.13–8.06 (m, 1H), 7.46 (d, *J* = 2.5 Hz, 1H), 7.29–7.20 (m, 2H), 6.93 (d, *J* = 7.5 Hz, 1H), 6.86 (d, *J* = 8.4 Hz, 1H), 6.79 (t, *J* = 2.1 Hz, 1H), 6.74 (dd, *J* = 8.2, 2.5 Hz, 1H), 3.17 (q, *J* = 6.9 Hz, 2H), 2.33 (s, 3H), 2.28 (s, 3H), 1.39–1.33 (m, 2H), 1.25–1.18 (m, 2H), 0.82 (t, *J* = 7.3 Hz, 3H). ^13^C NMR (126 MHz, DMSO-*d*_6_) δ 165.46, 157.60, 151.45, 139.94, 133.49, 132.44, 130.68, 130.01, 128.79, 124.26, 120.42, 118.80, 115.37, 39.09, 31.62, 21.45, 20.67, 19.97, 14.16.

*N*-Cyclohexyl-5-fluoro-2-phenoxybenzamide (**14r**): 45.1%; m.p. 103–105 °C; ^1^H NMR (500 MHz, DMSO-*d*_6_) δ 8.09 (d, *J* = 7.9 Hz, 1H), 7.45–7.30 (m, 4H), 7.12 (tt, *J* = 7.3, 1.1 Hz, 1H), 7.06 (dd, *J* = 9.0, 4.5 Hz, 1H), 7.00–6.93 (m, 2H), 3.68–3.61 (m, 1H), 1.70–1.59 (m, 4H), 1.53 (dt, *J* = 13.0, 3.7 Hz, 1H), 1.30–1.04 (m, 5H). ^13^C NMR (126 MHz, DMSO-*d*_6_) δ 163.37, 159.47, 157.68, 157.55, 149.46, 149.44, 131.33, 131.28, 130.39, 123.66, 122.89, 122.82, 118.69, 118.51, 117.96, 116.70, 116.50, 48.40, 32.46, 25.62, 24.85.

### 3.4. General Procedure Preparation of Product ***15a**–**15r***

To the solution of substituted 2-phenoxybenzaide (**14a**–**14r**, 1.0 mmol) in TFA (10 mL) was added KOH (0.11 g, 2.0 mmol) and iodobenzene (0.33 g, 1.5 mmol). The reaction solution was stirred at room temperature for 4 h. 10% aqueous Na_2_CO_3_ (30 mL) was added, then was extracted by acetate ester (15 mL × 3). The organic phase was sequentially washed with saturated saline (15 mL × 3), and dried over anhydrous MgSO_4_, filtered, and concentrated to yield the crude product, which was purified by flash chromatography (THF:EA = 1:2) to obtain the products **15a**–**15r**.

2-(4-Hydroxyphenoxy) benzamide (**15a**): 72.9%; m.p. 155–159 °C; ^1^H NMR (500 MHz, DMSO-*d*_6_) δ 9.41 (s, 1H), 7.76 (dd, *J* = 7.7, 1.8 Hz, 1H), 7.63 (s, 1H), 7.60–7.57 (m, 10H), 7.39 (ddd, *J* = 8.4, 7.3, 1.8 Hz, 1H), 7.13 (td, *J* = 7.5, 1.1 Hz, 1H), 6.99–6.92 (m, 2H), 6.85–6.78 (m, 2H), 6.73 (dd, *J* = 8.3, 1.1 Hz, 1H); ^13^C NMR (126 MHz, DMSO-*d*_6_) δ 167.06, 156.40, 154.69, 147.82, 132.38, 130.92, 126.03, 122.71, 121.71, 117.17, 116.76; HRMS [M + H]^+^ (C_13_H_12_NO_3_): Calculated: 230.0739; Found: 230.0796.

2-(4-Hydroxy-3-methylphenoxy)benzamide (**15b**): 75.2%; m.p. 198–200 °C; ^1^H NMR (500 MHz, DMSO-*d*_6_) δ 9.30 (s, 1H), 7.77 (dd, *J* = 7.7, 1.8 Hz, 1H), 7.59 (d, *J* = 12.5 Hz, 2H), 7.39 (ddd, *J* = 8.6, 7.3, 1.9 Hz, 1H), 7.12 (td, *J* = 7.5, 1.1 Hz, 1H), 6.88 (d, *J* = 2.9 Hz, 1H), 6.82 (d, *J* = 8.6 Hz, 1H), 6.80–6.71 (m, 2H), 2.13 (s, 3H). ^13^C NMR (126 MHz, DMSO-*d*_6_) δ 167.01, 156.53, 152.76, 147.53, 132.42, 130.94, 126.06, 125.80, 122.78, 122.62, 118.67, 117.23, 115.83, 16.55. HRMS [M + H]^+^ (C_14_H_14_NO_3_): Calculated: 244.0895; Found: 244.0969.

2-(3-Bromo-4-hydroxyphenoxy)benzamide (**15c**): 54.8%; m.p. 213–216 °C; ^1^H NMR (500 MHz, DMSO-*d*_6_) δ 10.20 (s, 1H), 7.72 (dd, *J* = 7.7, 1.8 Hz, 1H), 7.65 (s, 1H), 7.57 (s, 1H), 7.42 (ddd, *J* = 8.3, 7.3, 1.8 Hz, 1H), 7.29 (d, *J* = 2.5 Hz, 1H), 7.17 (td, *J* = 7.5, 1.1 Hz, 1H), 7.03–6.95 (m, 2H), 6.81 (dd, *J* = 8.3, 1.0 Hz, 1H). ^13^C NMR (126 MHz, DMSO-*d*_6_) δ 167.16, 155.49, 151.44, 148.59, 132.34, 130.77, 126.98, 124.61, 123.37, 120.71, 117.88, 117.26, 109.79. HRMS [M + H]^+^ (C_13_H_11_BrNO_3_): Calculated: 307.9844; Found: 307.9912.

2-(3-Chloro-4-hydroxyphenoxy)benzamide (**15d**): 54.4%; m.p. 210–214 °C; ^1^H NMR (500 MHz, DMSO-*d*_6_) δ 10.12 (s, 1H), 7.72 (dd, *J* = 7.7, 1.8 Hz, 1H), 7.64 (s, 1H), 7.56 (s, 1H), 7.42 (ddd, *J* = 8.4, 7.3, 1.8 Hz, 1H), 7.21–7.14 (m, 2H), 7.01 (d, *J* = 8.9 Hz, 1H), 6.93 (dd, *J* = 8.8, 2.9 Hz, 1H), 6.82 (dd, *J* = 8.3, 1.1 Hz, 1H); ^13^C NMR (126 MHz, DMSO-*d*_6_) δ 167.15, 155.44, 150.39, 148.43, 132.33, 130.77, 127.01, 123.40, 121.79, 120.49, 120.06, 117.94, 117.66. HRMS [M + H]^+^ (C_13_H_11_ClNO_3_): Calculated: 264.0349; Found: 264.0420.

2-(4-Hydroxyphenoxy)-5-methylbenzamide (**15e**): 60.0%; m.p. 160–163 °C; ^1^H NMR (500 MHz, DMSO-*d*_6_) δ 9.37 (s, 1H), 7.61–7.52 (m, 3H), 7.21 (dd, *J* = 8.5, 2.4 Hz, 1H), 6.95–6.88 (m, 2H), 6.83–6.76 (m, 2H), 6.67 (d, *J* = 8.4 Hz, 1H), 2.30 (s, 3H); ^13^C NMR (126 MHz, DMSO-*d*_6_) δ 167.03, 154.41, 154.09, 148.43, 132.87, 131.95, 131.14, 125.82, 121.21, 117.80, 116.68, 20.55. HRMS [M + H]^+^ (C_13_H_14_NO_3_): Calculated: 244.0895; Found: 244.0966.

2-(4-Hydroxy-3-methylphenoxy)-5-methylbenzamide (**15f**): 55.7%; m.p. 192–195 °C; ^1^H NMR (500 MHz, DMSO-*d*_6_) δ 9.26 (s, 1H), 7.59 (d, *J* = 2.3 Hz, 1H), 7.55 (d, *J* = 9.4 Hz, 2H), 7.20 (dd, *J* = 8.4, 2.4 Hz, 1H), 6.83 (d, *J* = 2.9 Hz, 1H), 6.79 (d, *J* = 8.6 Hz, 1H), 6.73 (dd, *J* = 8.6, 3.0 Hz, 1H), 6.67 (d, *J* = 8.4 Hz, 1H), 2.29 (s, 3H), 2.12 (s, 3H). ^13^C NMR (126 MHz, DMSO-*d*_6_) δ 166.98, 154.21, 152.48, 148.14, 132.92, 131.85, 131.15, 125.95, 125.60, 122.30, 118.17, 117.86, 115.76, 20.55, 16.54. HRMS [M + H]^+^ (C_14_H_14_NO_3_): Calculated: 258.1052; Found: 258.1121.

5-Chloro-2-(4-hydroxy-3-methylphenoxy)benzamide (**15g**): 68.2%; m.p. 210–212 °C; ^1^H NMR (500 MHz, DMSO-*d*_6_) δ 9.35 (s, 1H), 7.71 (q, *J* = 3.5 Hz, 3H), 7.43 (dd, *J* = 8.9, 2.8 Hz, 1H), 6.90 (d, *J* = 2.8 Hz, 1H), 6.86–6.77 (m, 2H), 6.75 (d, *J* = 8.8 Hz, 1H), 2.13 (s, 3H). ^13^C NMR (126 MHz, DMSO-*d*_6_) δ 165.75, 155.39, 153.00, 147.31, 131.94, 130.07, 127.62, 126.39, 126.18, 122.82, 119.08, 118.75, 115.87, 16.53. HRMS [M + H]^+^ (C_14_H_13_ClNO_3_): Calculated: 278.0506; Found: 278.0578.

5-Chloro-2-((6-hydroxy-[1,1′-biphenyl]-3-yl)oxy)benzamide (**15h**): 56.9%; m.p. 200–202 °C; ^1^H NMR (500 MHz, DMSO-*d*_6_) δ 9.65 (s, 1H), 7.86–7.64 (m, 3H), 7.58 (d, *J* = 7.1 Hz, 2H), 7.47–7.38 (m, 3H), 7.32 (t, *J* = 7.3 Hz, 1H), 7.09 (d, *J* = 2.8 Hz, 1H), 7.00 (qd, *J* = 8.7, 2.8 Hz, 2H), 6.87 (d, *J* = 8.9 Hz, 1H). ^13^C NMR (126 MHz, DMSO-*d*_6_) δ 165.87, 155.03, 151.81, 148.08, 138.18, 131.93, 130.03, 129.54, 129.33, 128.50, 128.15, 127.43, 126.62, 122.31, 120.58, 119.35, 117.64. HRMS [M + H]^+^ (C_14_H_15_ClNO_3_): Calculated: 340.0662; Found: 340.0727.

5-Fluoro-2-(4-hydroxyphenoxy)benzamide (**15i**): 89.5%; m.p. 193–195 °C; ^1^H NMR (500 MHz, DMSO-*d*_6_) δ 9.41 (s, 1H), 7.72 (s, 2H), 7.51 (dd, *J* = 9.0, 3.3 Hz, 1H), 7.26 (ddd, *J* = 9.1, 7.8, 3.3 Hz, 1H), 6.97–6.90 (m, 2H), 6.80 (dt, *J* = 9.0, 2.3 Hz, 3H). ^13^C NMR (126 MHz, DMSO-*d*_6_) δ 165.80, 158.45, 156.55, 154.60, 152.36, 148.39, 127.93, 127.88, 121.24, 119.72, 119.65, 119.06, 118.87, 116.87, 116.76, 116.67. HRMS [M + H]^+^ (C_13_H_11_FNO_3_): Calculated: 248.0645; Found: 248.0713.

5-Fluoro-2-(4-hydroxy-3-methylphenoxy)benzamide (**15j**): 75.6%; m.p. 221–223 °C; ^1^H NMR (500 MHz, DMSO-*d*_6_) δ 9.29 (s, 1H), 7.70 (d, *J* = 12.4 Hz, 2H), 7.52 (dd, *J* = 9.1, 3.3 Hz, 1H), 7.26 (td, *J* = 8.4, 3.3 Hz, 1H), 6.86 (d, *J* = 2.9 Hz, 1H), 6.84–6.78 (m, 2H), 6.75 (dd, *J* = 8.7, 2.9 Hz, 1H), 2.12 (s, 3H). ^13^C NMR (126 MHz, DMSO-*d*_6_) δ 165.75, 165.69, 158.40, 156.50, 152.68, 152.49, 148.10, 127.63, 126.08, 122.33, 119.74, 119.68, 119.12, 118.93, 118.20, 116.85, 116.65, 115.82, 16.53. HRMS [M + H]^+^ (C_14_H_13_FNO_3_): Calculated: 262.0801; Found: 262.0870.

5-Fluoro-2-((6-hydroxy-[1,1′-biphenyl]-3-yl)oxy)benzamide (**15k**): 61.2%; m.p. 196–198 °C; ^1^H NMR (500 MHz, DMSO-*d*_6_) δ 9.59 (s, 1H), 7.75 (d, *J* = 11.2 Hz, 2H), 7.60–7.55 (m, 2H), 7.51 (dd, *J* = 9.0, 3.3 Hz, 1H), 7.41 (t, *J* = 7.7 Hz, 2H), 7.35–7.25 (m, 2H), 7.05 (d, *J* = 2.9 Hz, 1H), 6.99 (d, *J* = 8.7 Hz, 1H), 6.96–6.91 (m, 2H). ^13^C NMR (126 MHz, DMSO-*d*_6_) δ 165.89, 158.56, 156.65, 152.07, 151.46, 148.87, 138.26, 129.53, 129.24, 128.50, 127.39, 121.74, 119.99, 119.07, 118.88, 117.57, 116.85, 116.66. HRMS [M + H]^+^ (C_19_H_15_FNO_3_): Calculated: 324.0958; Found: 324.1016.

*N*-Butyl-2-(4-hydroxyphenoxy)benzamide (**15l**): 66.7%; m.p. 141–144 °C; ^1^H NMR (500 MHz, DMSO-*d*_6_) δ 9.39 (s, 1H), 8.16 (t, *J* = 5.7 Hz, 1H), 7.66 (dd, *J* = 7.7, 1.8 Hz, 1H), 7.38 (ddd, *J* = 8.3, 7.3, 1.8 Hz, 1H), 7.13 (td, *J* = 7.5, 1.1 Hz, 1H), 6.97–6.90 (m, 2H), 6.84–6.78 (m, 2H), 6.76 (dd, *J* = 8.3, 1.1 Hz, 1H), 3.26 (q, *J* = 6.6 Hz, 2H), 1.49–1.43(m, 2H), 1.34–1.26 (m, 2H), 0.87 (t, *J* = 7.3 Hz, 3H). ^13^C NMR (126 MHz, DMSO-*d*_6_) δ 165.55, 155.93, 154.56, 148.13, 131.90, 130.50, 127.07, 122.83, 121.44, 117.42, 116.69, 39.18, 31.65, 20.03, 14.17. HRMS [M + H]^+^ (C_17_H_20_NO_3_): Calculated: 286.1365; Found: 286.1436.

2-(4-Hydroxyphenoxy)-*N*-methyl-*N*-phenylbenzamide (**15m**): 45.1%; m.p. 168–170 °C; ^1^H NMR (500 MHz, DMSO-*d*_6_) δ 9.39 (s, 1H), 7.36–7.08 (m, 7H), 6.92 (d, *J* = 7.6 Hz, 1H), 6.76 (d, *J* = 8.3 Hz, 2H), 6.59 (d, *J* = 7.9 Hz, 2H), 6.37 (d, *J* = 8.4 Hz, 1H), 3.38 (s, 3H). ^13^C NMR (126 MHz, DMSO-*d*_6_) δ 167.78, 154.59, 154.46, 147.42, 144.04, 130.54, 129.60, 129.20, 128.09, 127.36, 127.04, 121.99, 121.68, 116.66, 115.35, 37.14. HRMS [M + H]^+^ (C_20_H_18_NO_3_): Calculated: 320.1208; Found: 320.1278.

*N*-Cyclohexyl-2-(4-hydroxyphenoxy)benzamide (**15n**): 55.6%; m.p. 168–170 °C; ^1^H NMR (500 MHz, DMSO-*d*_6_) δ 9.37 (s, 1H), 8.00 (d, *J* = 7.9 Hz, 1H), 7.65 (dd, *J* = 7.7, 1.8 Hz, 1H), 7.39 (ddd, *J* = 8.6, 7.4, 1.8 Hz, 1H), 7.14 (td, *J* = 7.5, 1.1 Hz, 1H), 6.95–6.91 (m, 2H), 6.82–6.76 (m, 3H), 3.79–3.71 (m, 1H), 1.80–1.76(m, 2H), 1.68–1.64(m, 2H), 1.58–1.50 (m, 1H), 1.33–1.23 (m, 4H), 1.19–1.13 (m, 1H). ^13^C NMR (126 MHz, DMSO-*d*_6_) δ 164.67, 155.76, 154.49, 148.28, 131.91, 130.47, 127.31, 123.00, 121.16, 117.76, 116.71, 48.24, 32.66, 25.68, 24.84. HRMS [M + H]^+^ (C_19_H_22_NO_3_): Calculated: 312.1521; Found: 312.1578.

(2-(4-Hydroxyphenoxy)phenyl)(pyrrolidin-1-yl)methanone (**15o**): 58.5%; m.p. 205–207 °C; ^1^H NMR (500 MHz, DMSO-*d*_6_) δ 9.38 (s, 1H), 7.37–7.28 (m, 2H), 7.11 (td, *J* = 7.5, 1.0 Hz, 1H), 6.92–6.85 (m, 2H), 6.82–6.73 (m, 3H), 3.42 (t, *J* = 6.8 Hz, 2H), 3.26 (t, *J* = 6.5 Hz, 2H), 1.88–1.76 (m, 4H). ^13^C NMR (126 MHz, DMSO-*d*_6_) δ 166.24, 154.53, 148.09, 130.71, 129.23, 128.58, 122.97, 121.48, 116.99, 116.72, 47.84, 45.64, 25.95, 24.51. HRMS [M + H]^+^ (C_17_H_18_NO_3_): Calculated: 284.1208; Found: 284.1275.

(2-(4-Hydroxyphenoxy)phenyl)(piperidin-1-yl)methanone (**15p**): 20.7%; m.p. 174–176 °C; ^1^H NMR (500 MHz, DMSO-*d*_6_) δ 9.39 (s, 1H), 7.32 (ddd, *J* = 8.6, 7.4, 1.8 Hz, 1H), 7.28 (dd, *J* = 7.6, 1.8 Hz, 1H), 7.11 (td, *J* = 7.4, 1.0 Hz, 1H), 6.92–6.85 (m, 2H), 6.83–6.76 (m, 2H), 6.72 (dd, *J* = 8.3, 1.0 Hz, 1H), 3.58 (m, 2H), 3.23 (q, *J* = 6.3 Hz, 2H), 1.59 (m, 2H), 1.55–1.38 (m, 4H). ^13^C NMR (126 MHz, DMSO-*d*_6_) δ 166.20, 154.54, 154.47, 148.05, 130.53, 128.55, 128.06, 122.99, 121.43, 116.78, 116.55, 47.85, 42.18, 26.49, 25.80, 24.50. HRMS [M + H]^+^ (C_18_H_20_NO_3_): Calculated: 298.1416; Found: 298.1426.

*N*-Butyl-2-(4-hydroxy-3-methylphenoxy)-5-methylbenzamide (**15q**): 50.5%; m.p. 124–126 °C; ^1^H NMR (500 MHz, DMSO-*d*_6_) δ 9.22 (s, 1H), 8.09 (t, *J* = 5.7 Hz, 1H), 7.48 (d, *J* = 2.3 Hz, 1H), 7.19 (dd, *J* = 8.5, 2.4 Hz, 1H), 6.82–6.75 (m, 2H), 6.73–6.66 (m, 2H), 3.24 (q, *J* = 6.8 Hz, 2H), 2.29 (s, 3H), 2.11 (s, 3H), 1.47–1.41 (m, 2H), 1.32–1.24 (m, 2H), 0.86 (t, *J* = 7.3 Hz, 3H). ^13^C NMR (126 MHz, DMSO-*d*_6_) δ 165.49, 153.69, 152.33, 148.45, 132.39, 131.98, 130.74, 126.72, 125.83, 122.04, 118.12, 117.89, 115.69, 39.16, 31.67, 20.55, 20.03, 16.56, 14.17. HRMS [M + H]^+^ (C_19_H_24_NO_3_): Calculated: 314.1729; Found: 314.1751.

*N*-Cyclohexyl-5-fluoro-2-(4-hydroxyphenoxy)benzamide (**15r**): 42.3%; m.p. 178–180 °C; ^1^H NMR (500 MHz, DMSO-*d*_6_) δ 9.36 (s, 1H), 8.09 (d, *J* = 7.9 Hz, 1H), 7.41 (dd, *J* = 8.9, 3.3 Hz, 1H), 7.26 (ddd, *J* = 9.1, 7.9, 3.3 Hz, 1H), 6.93–6.88 (m, 2H), 6.86 (dd, *J* = 9.1, 4.5 Hz, 1H), 6.81–6.75 (m, 2H), 3.78–3.67 (m, 1H), 1.80–1.72 (m, 2H), 1.68–1.63 (m, 2H), 1.54 (dt, *J* = 8.8, 3.8 Hz, 1H), 1.34–1.19 (m, 4H), 1.18–1.08 (m, 1H). ^13^C NMR (126 MHz, DMSO-*d*_6_) δ 163.42, 158.66, 156.75, 154.37, 151.67, 151.65, 148.89, 129.26, 129.20, 120.61, 120.39, 120.32, 118.56, 118.37, 116.69, 116.58, 116.39, 48.38, 32.55, 25.65, 24.81. HRMS [M + H]^+^ (C_19_H_21_FNO_3_): Calculated: 330.1427; Found: 330.1493.

## 4. Conclusions

We report the synthesis and characterization of 2-(4-hydroxyphenoxy)benzamide compounds, presenting a robust methodology for their preparation. The phenolic derivatives were synthesized by employing TFA as the solvent and hypervalent iodine as the oxidant at room temperature. This method effectively yielded target compounds with diverse substituent groups, including alkyl, halogen, and phenyl groups, with yields ranging from 20.7% to 89.5%. In addition, the methodology demonstrated good substrate tolerance, making a valuable contribution to advancing synthetic strategies in organic chemistry. Substrate expansion efforts further validated the versatility of this method for synthesizing phenolic derivatives. Further studies will focus on optimizing reaction conditions and conducting a more comprehensive study of substrate suitability. Further investigations will also delve into the mechanistic aspects of the reaction and explore the potential applications of these compounds in various chemical contexts.

## Figures and Tables

**Table 1:** Optimization of oxidation conditions.

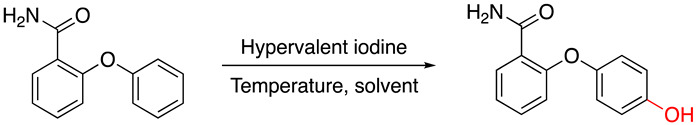						
Entry	Hypervalent Iodine	n(Substrate:Hypervalent Iodine)	Solvent	Temp. (°C)	Time (h)	Yield (%)
1	—	—	TFA	rt–60	—	—
2	PhIO	1:1.2	TFA	rt	5	65.1
3	PhIO	1:1.5	TFA	rt	4	64.7
4	PhIO	1:2	TFA	rt	2	72.9
5	PhIO	1:2.5	TFA	rt	2	28.5
6	PhIO	1:2	TFA	0–5	4	68.1
7	PhIO	1:2	TFA	60	1	56.0
8	PIDA	1:2	TFA	rt	4	25.4
9	PIFA	1:2	TFA	rt	1.5	43.3
10	Koser’s reagent	1:2	TFA	rt	1	0
11	PhIO	1:2	AcOH	rt	—	—
12	PhIO	1:2	TfOH	rt	—	—
13	PhIO	1:2	TFE	rt	—	—
14	PIFA	1:2	HFIP	rt	2	58.2

**Table 2:** PhIO-mediated oxidation reaction for the synthesis of 2-(hydroxyphenoxy) benzamide derivatives ^a^.

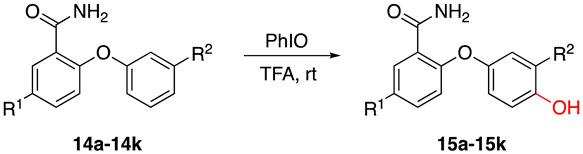					
Entry	Substrate	Product	R1	R2	Yield (%) ^b^
1	**14a**	**15a**	H	H	72.9
2	**14b**	**15b**	H	CH_3_	75.2
3	**14c**	**15c**	H	Br	54.8
4	**14d**	**15d**	H	Cl	54.4
5	**14e**	**15e**	CH_3_	H	60.0
6	**14f**	**15f**	CH_3_	CH_3_	55.7
7	**14g**	**15g**	Cl	CH_3_	68.2
8	**14h**	**15h**	Cl	Ph	56.9
9	**14i**	**15i**	F	H	89.5
10	**14j**	**15j**	F	CH_3_	75.6
11	**14k**	**15k**	F	Ph	61.2

^a^ Reaction conditions: hypervalent iodine (2 eq). ^b^ Isolated yields.

**Table 3:** PhIO-mediated oxidation reaction for the synthesis of 2-(hydroxyphenoxy) benzamide derivatives ^a^.

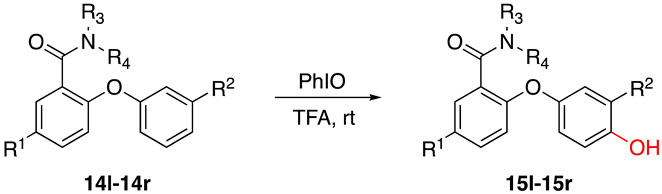								
Entry	Substrate	Product	R^1^	R^2^	R^3^	R^4^	Time (h)	Yield (%) ^b^
1	**14l**	**15l**	H	H	H	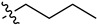	1.0	68.2
2	**14m**	**15m**	H	H	Me	Ph	1.0	45.1
3	**14n**	**15n**	H	H	H	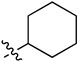	1.0	55.6
4	**14o**	**15o**	H	H	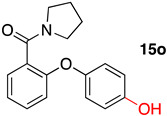		1.5	58.5
5	**14p**	**15p**	H	H	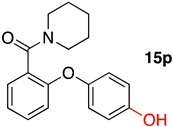		2.0	20.7
6	**14q**	**15q**	CH_3_	CH_3_	H	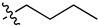	2.0	50.5
7	**14r**	**15r**	F	H	H	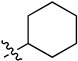	1.0	62.3

^a^ Reaction conditions: hypervalent iodine (1.5 eq). ^b^ Isolated yields.

**Table 4:** PhIO-mediated oxidation reaction for the synthesis of 2-(hydroxyphenoxy) benzamide derivatives.

Entry	Substrate	Product	Yield (%)
1	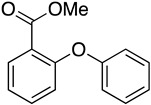	—	—
2	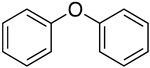	—	—
3	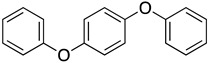	—	—
4	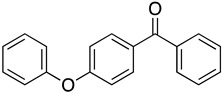	—	—
5	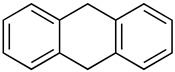	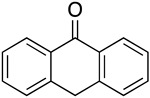	76.8
6	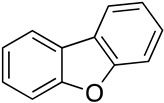	—	—
7	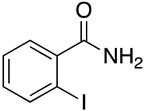	—	—
8	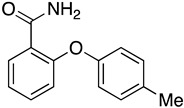	—	—

## Data Availability

Data are contained within the article and Supplementary Materials.

## Supplementary Materials

The following supporting information can be downloaded at: https://www.mdpi.com/article/10.3390/molecules29246048/s1.
