# Supplementary material for: Direct Synthesis of 2-(4-Hydroxyphenoxy)benzamide Derivatives from 2-Aryloxybenzamide via PhIO-Mediated Oxidation Reaction
Source: Molecules. 2024 Dec 22;29(24):6048. doi: 10.3390/molecules29246048 (PMC11679658; doi:10.3390/molecules29246048)
Supplement: Supplementary file 1 [file molecules-29-06048-s001.zip › molecules-3345349-supplementary.pdf]

Table of Contents

1. ^{1}H-NMR, ^{13}C-NMR of compound **14a** S_1-S_2
2. ^{1}H-NMR, ^{13}C-NMR of compound **14b** S_3-S_4
3. ^{1}H-NMR, ^{13}C-NMR of compound **14c** S_5-S_6
4. ^{1}H-NMR, ^{13}C-NMR of compound **14d** S_7-S_8
5. ^{1}H-NMR, ^{13}C-NMR of compound **14e** S_9-S_{10}
6. ^{1}H-NMR, ^{13}C-NMR of compound **14f** S_{11}-S_{12}
7. ^{1}H-NMR, ^{13}C-NMR of compound **14g** S_{13}-S_{14}
8. ^{1}H-NMR, ^{13}C-NMR of compound **14h** S_{15}-S_{16}
9. ^{1}H-NMR, ^{13}C-NMR of compound **14i** S_{17}-S_{18}
10. ^{1}H-NMR, ^{13}C-NMR of compound **14j** S_{19}-S_{20}
11. ^{1}H-NMR, ^{13}C-NMR of compound **14k** S_{21}-S_{22}
12. ^{1}H-NMR, ^{13}C-NMR of compound **14l** S_{23}-S_{24}
13. ^{1}H-NMR, ^{13}C-NMR of compound **14m** S_{25}-S_{26}
14. ^{1}H-NMR, ^{13}C-NMR of compound **14n** S_{27}-S_{28}
15. ^{1}H-NMR, ^{13}C-NMR of compound **14o** S_{29}-S_{30}
16. ^{1}H-NMR, ^{13}C-NMR of compound **14p** S_{31}-S_{32}
17. ^{1}H-NMR, ^{13}C-NMR of compound **14q** S_{33}-S_{34}
18. ^{1}H-NMR, ^{13}C-NMR of compound **14r** S_{35}-S_{36}
19. ^{1}H-NMR, ^{13}C-NMR, HRMS of compound **15a** S_{37}-S_{39}
20. ^{1}H-NMR, ^{13}C-NMR, HRMS of compound **15b** S_{40}-S_{42}
21. ^{1}H-NMR, ^{13}C-NMR, HRMS of compound **15c** S_{43}-S_{45}
22. ^{1}H-NMR, ^{13}C-NMR, HRMS of compound **15d** S_{46}-S_{48}
23. ^{1}H-NMR, ^{13}C-NMR, HRMS of compound **15e** S_{49}-S_{51}
24. ^{1}H-NMR, ^{13}C-NMR, HRMS of compound **15f** S_{52}-S_{54}
25. ^{1}H-NMR, ^{13}C-NMR, HRMS of compound **15g** S_{55}-S_{57}
26. ^{1}H-NMR, ^{13}C-NMR, HRMS of compound **15h** S_{58}-S_{60}
27. ^{1}H-NMR, ^{13}C-NMR, HRMS of compound **15i** S_{61}-S_{63}
28. ^{1}H-NMR, ^{13}C-NMR, HRMS of compound **15j** S_{64}-S_{66}
29. ^{1}H-NMR, ^{13}C-NMR, HRMS of compound **15k** S_{67}-S_{69}
30. ^{1}H-NMR, ^{13}C-NMR, HRMS of compound **15l** S_{70}-S_{72}

31. ^{1}H-NMR, ^{13}C-NMR, HRMS of compound **15m** S_{73}-S_{75}

32. ^{1}H-NMR, ^{13}C-NMR, HRMS of compound **15n** S_{76}-S_{78}

33. ^{1}H-NMR, ^{13}C-NMR, HRMS of compound **15o** S_{79}-S_{81}

34. ^{1}H-NMR, ^{13}C-NMR, HRMS of compound **15p** S_{82}-S_{84}

35. ^{1}H-NMR, ^{13}C-NMR, HRMS of compound **15q** S_{85}-S_{87}

36. ^{1}H-NMR, ^{13}C-NMR, HRMS of compound **15r** S_{88}-S_{90}

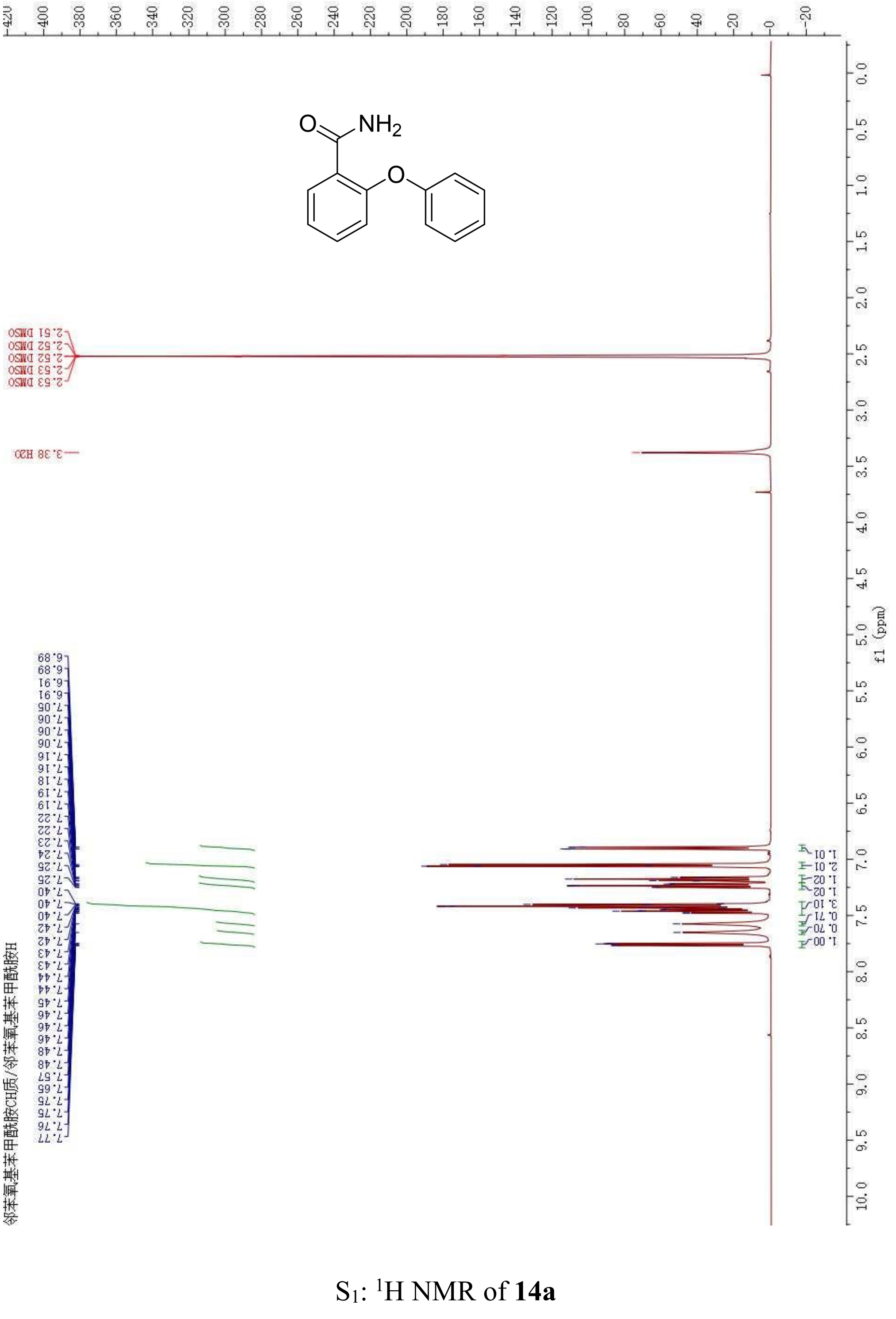

S$_1$: ^{1}H NMR of **14a**

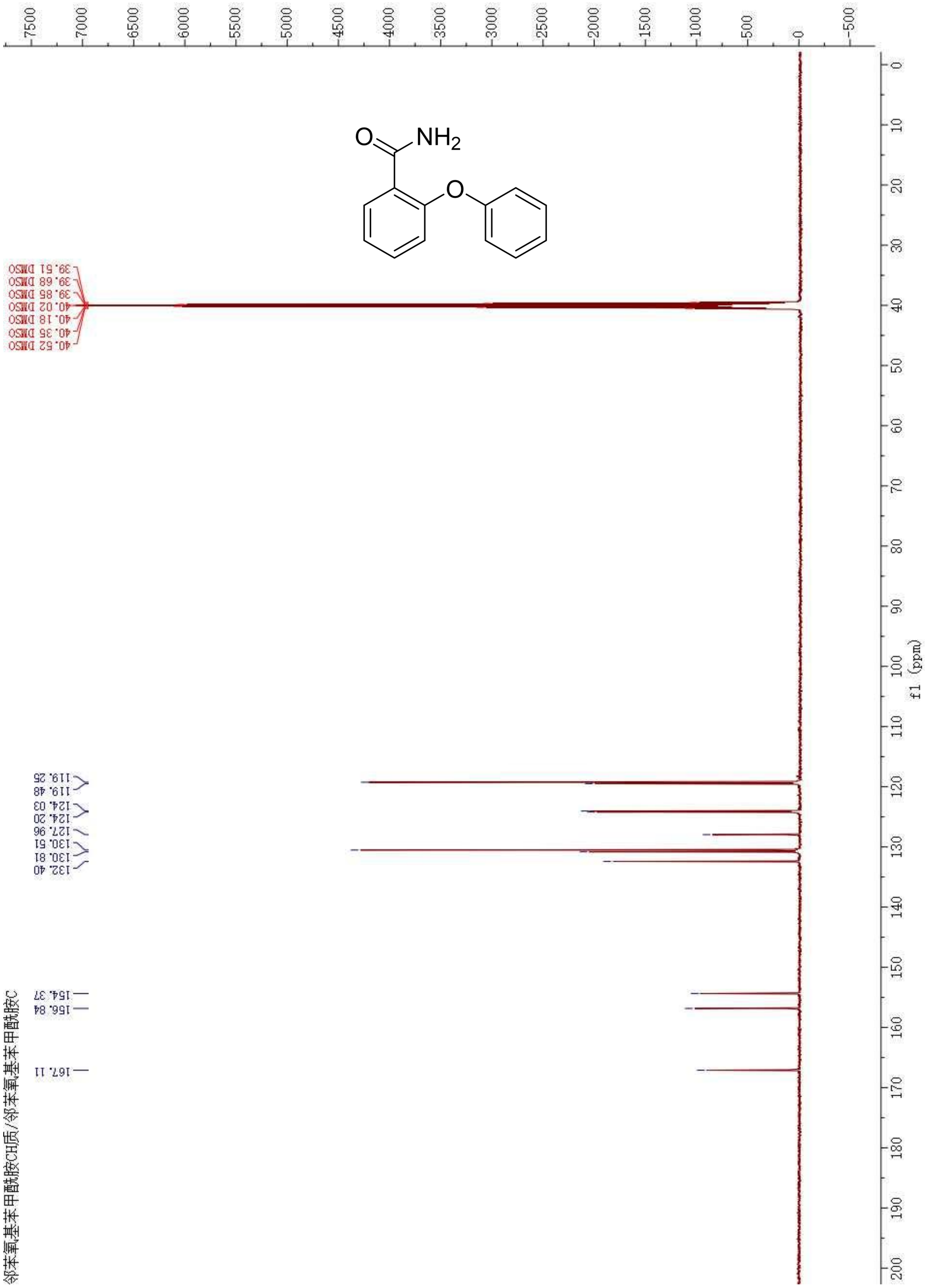

S$_2$: ^{13}C-NMR of **14a**

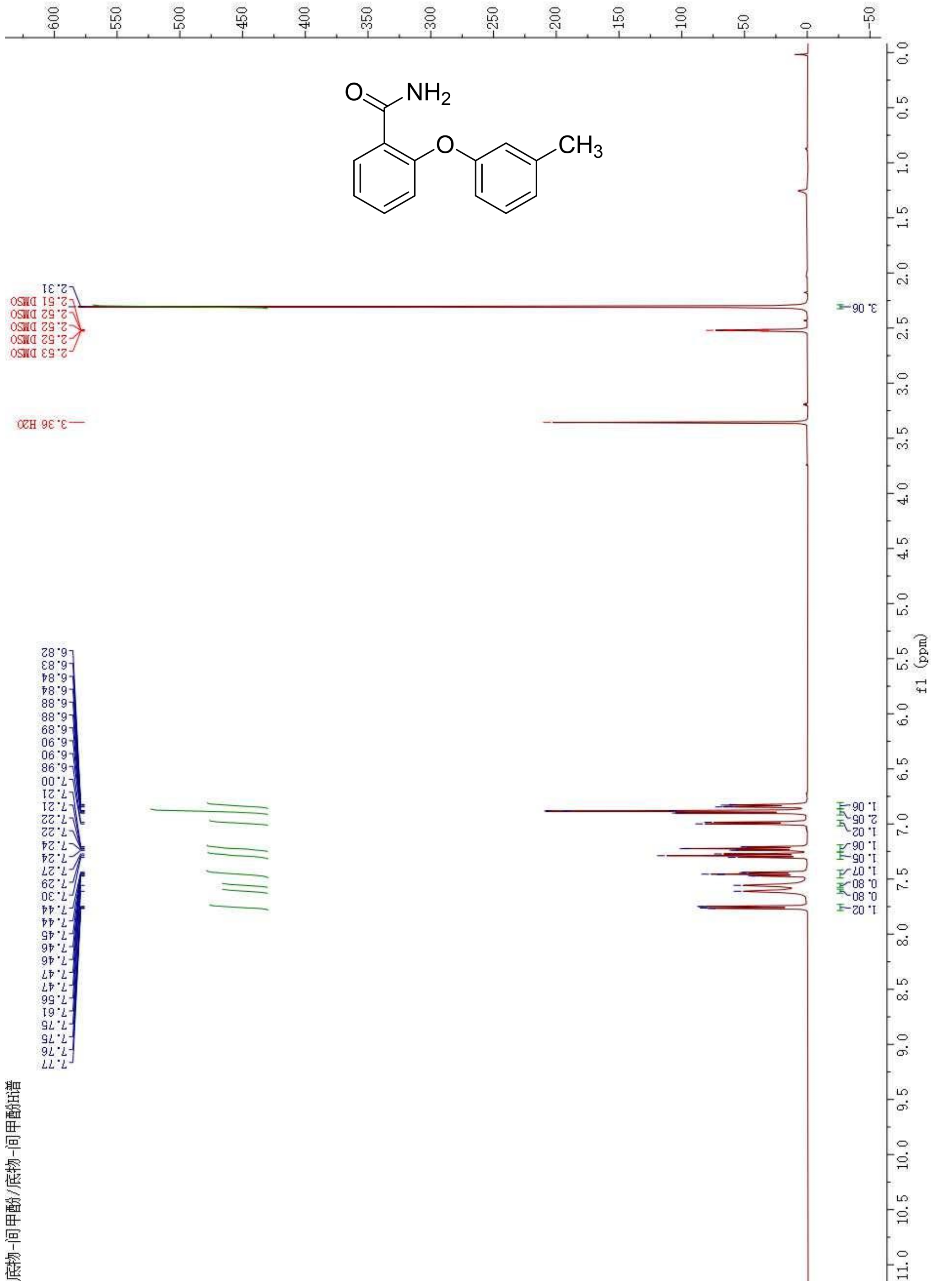

S$_3$: ^{1}H-NMR of **14b**

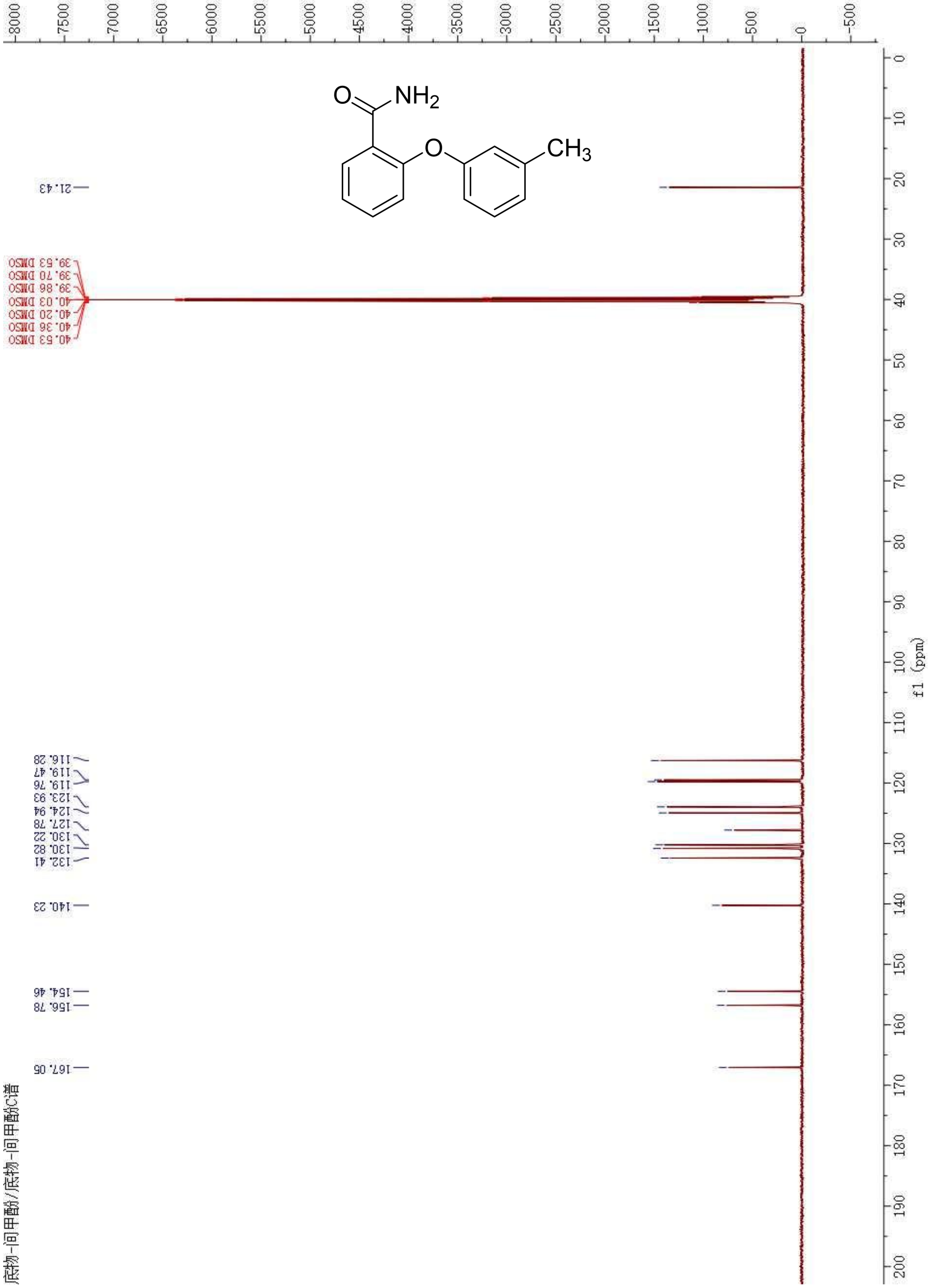

S4: ^{13}C-NMR of **14b**

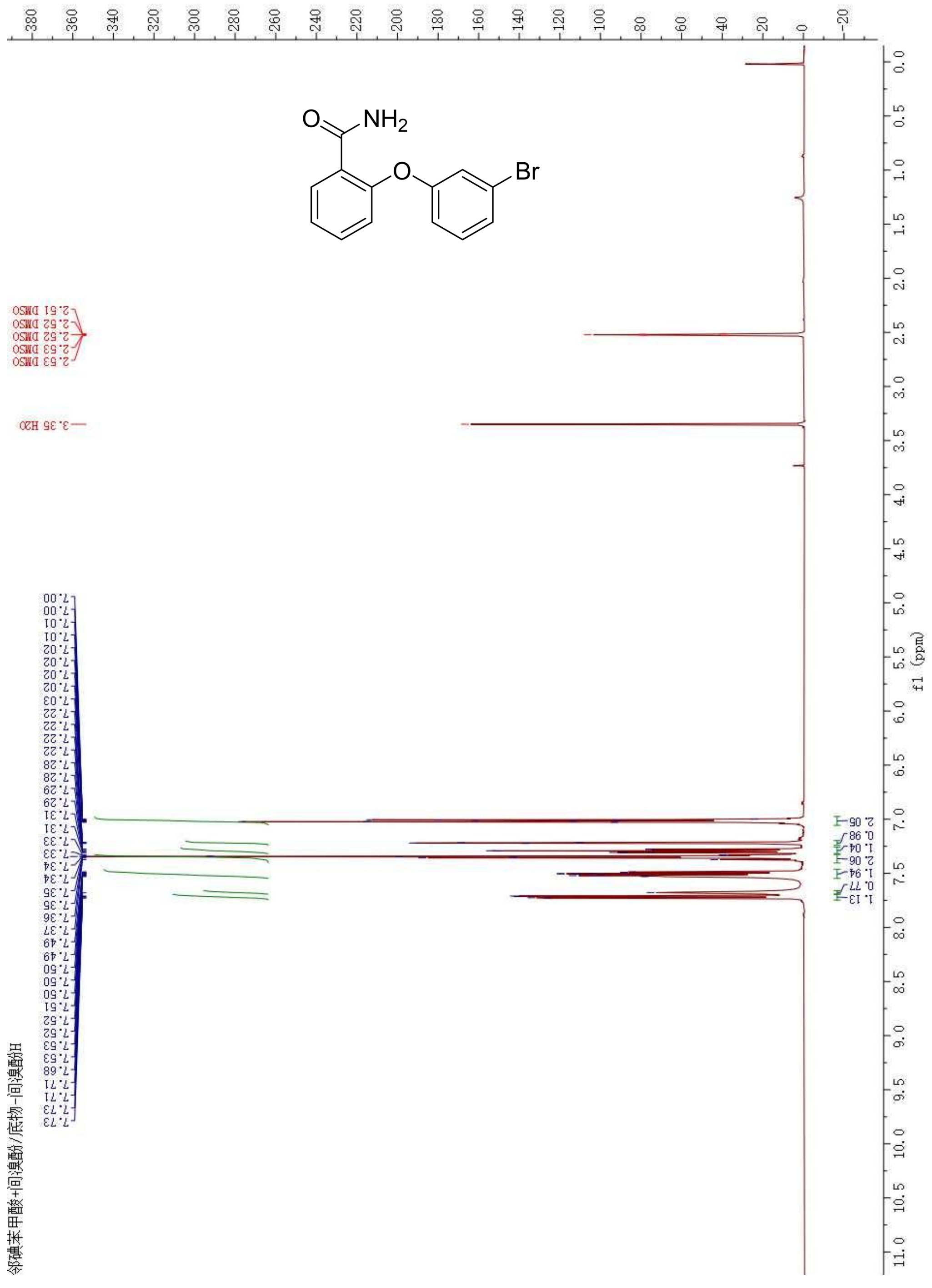

S$_5$: ^{1}H-NMR of **14c**

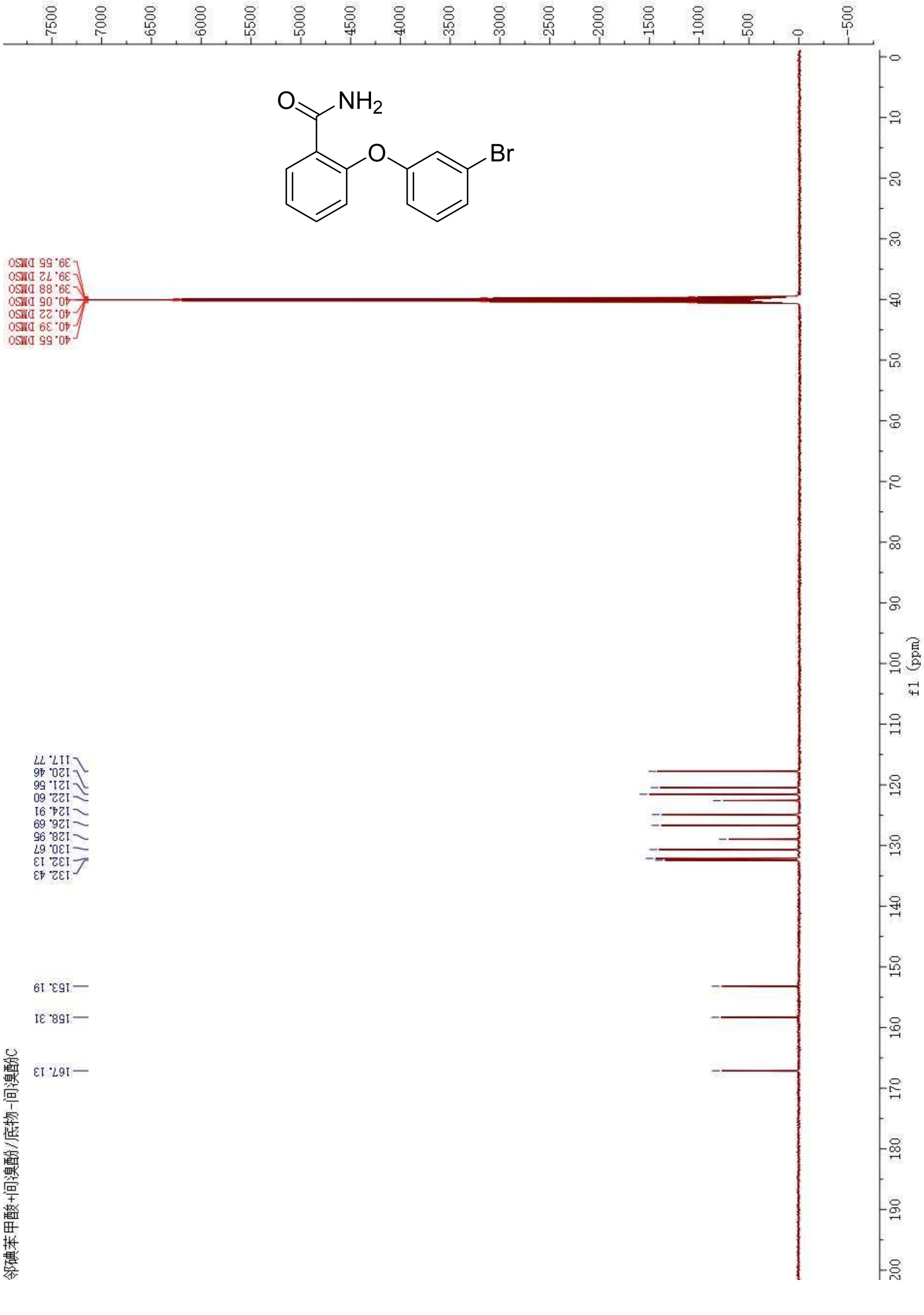

S$_6$: ^{13}C-NMR of **14c**

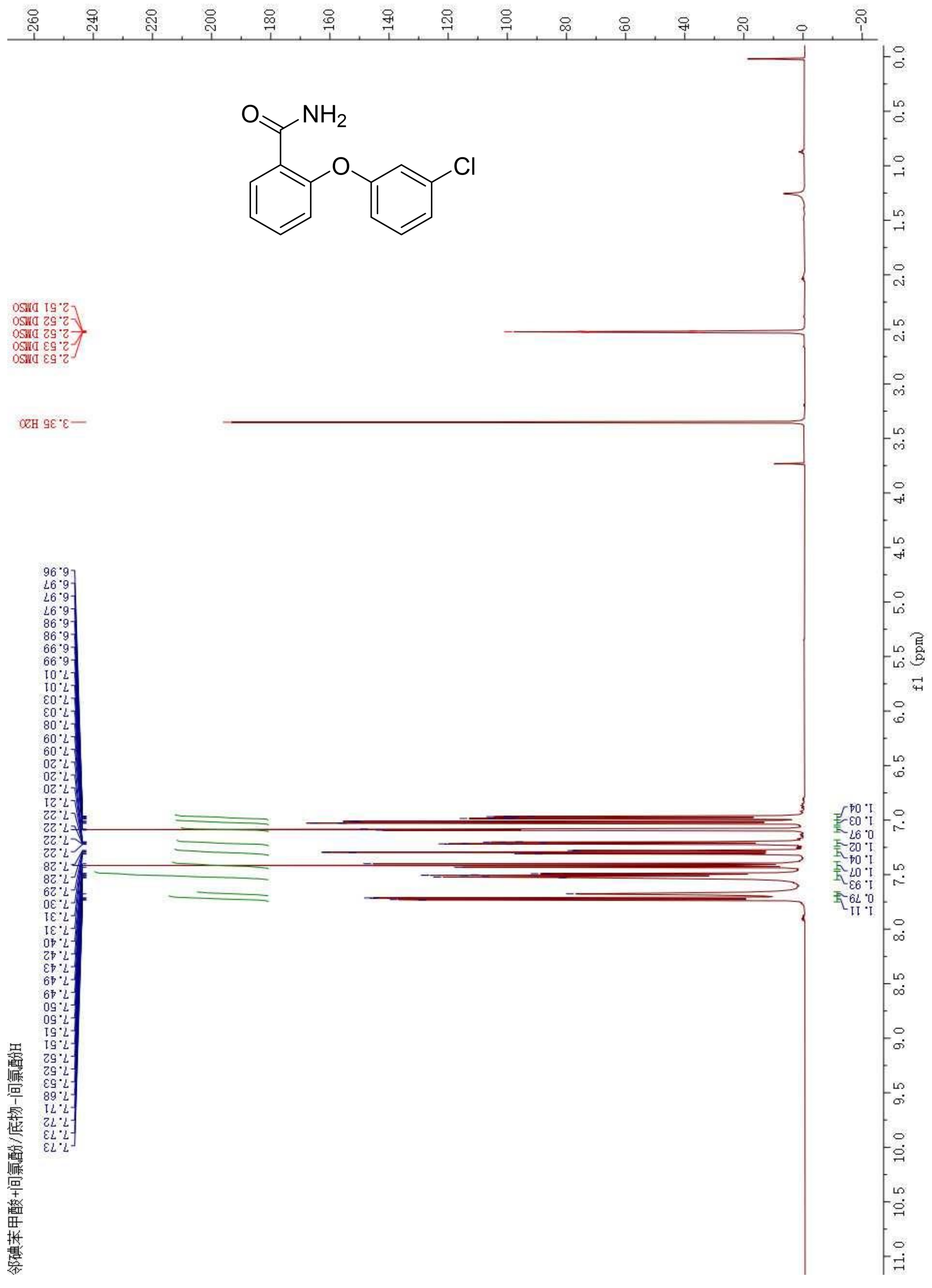

S$_7$: ^{1}H-NMR of **14d**

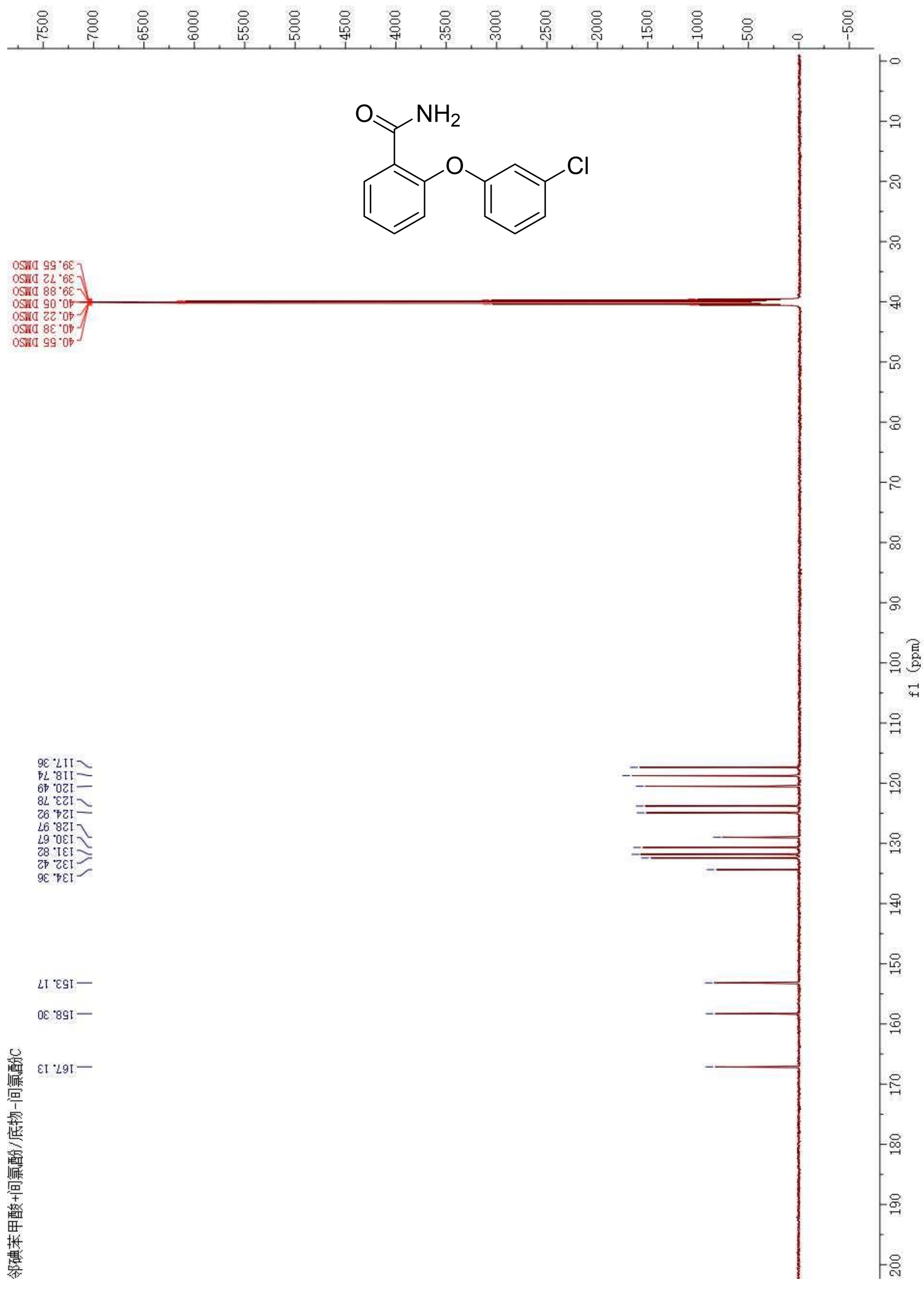

S8: ^{13}C-NMR of **14d**

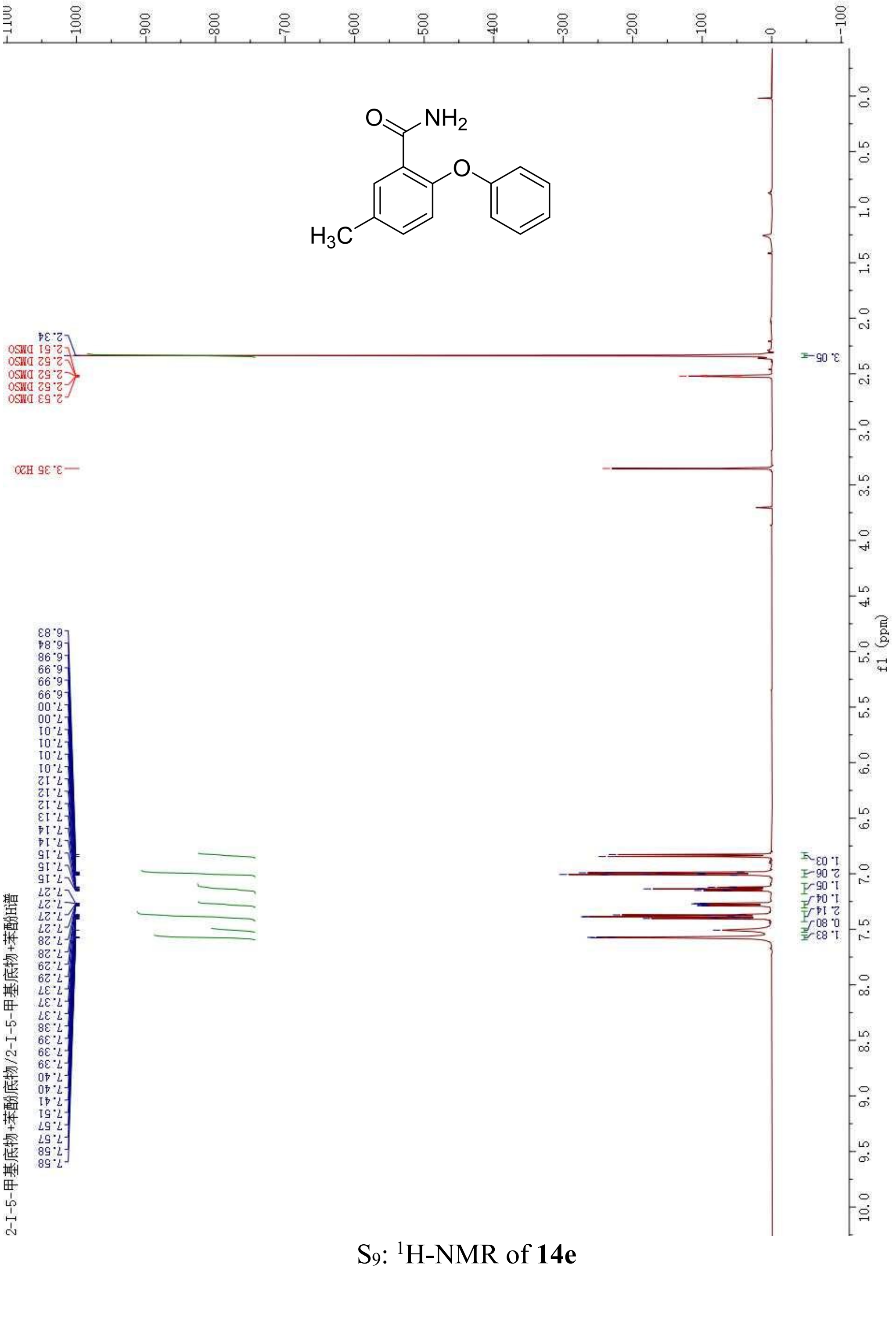

S9: ^{1}H-NMR of **14e**

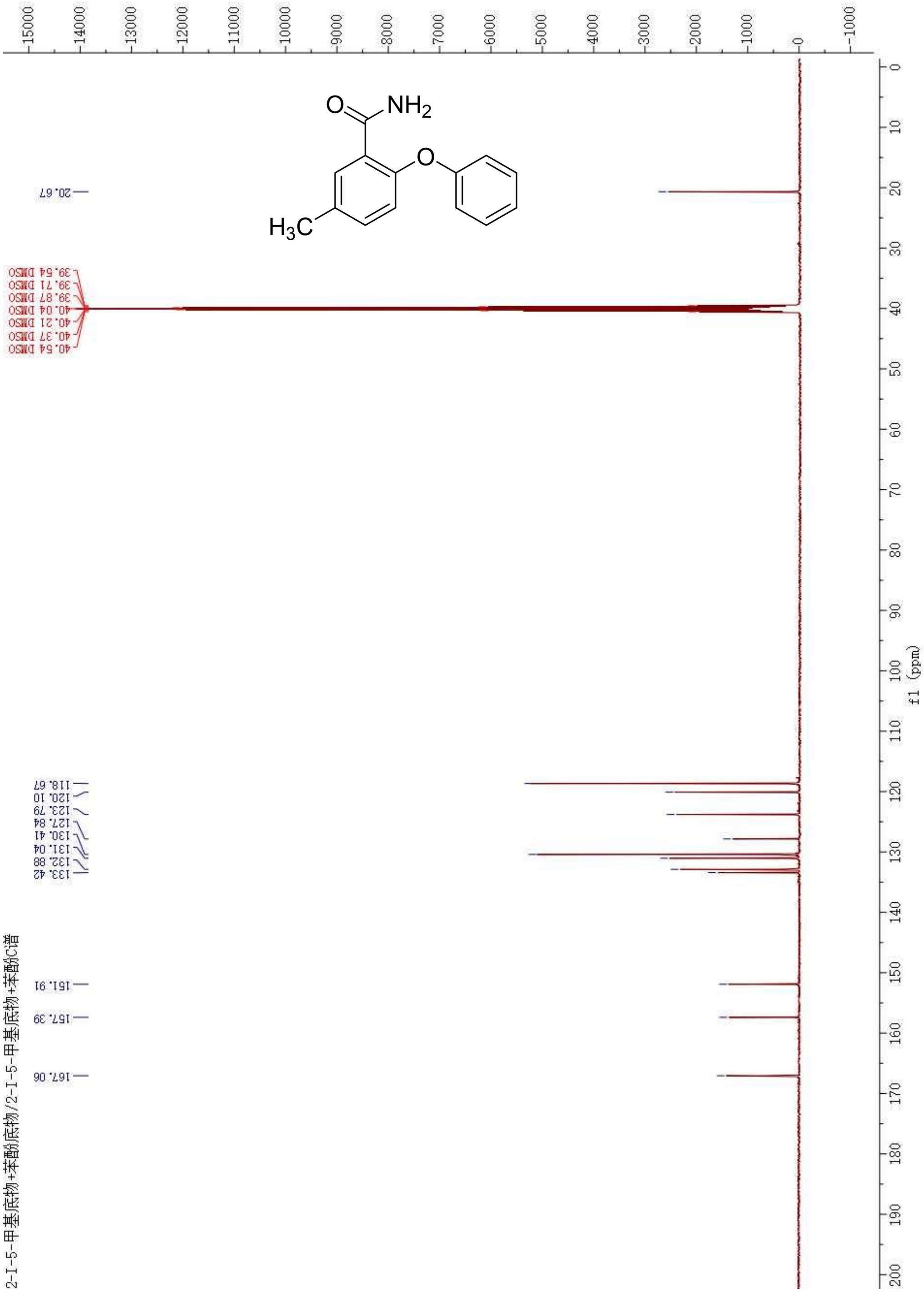

S$_{10}$: ^{13}C-NMR of **14e**

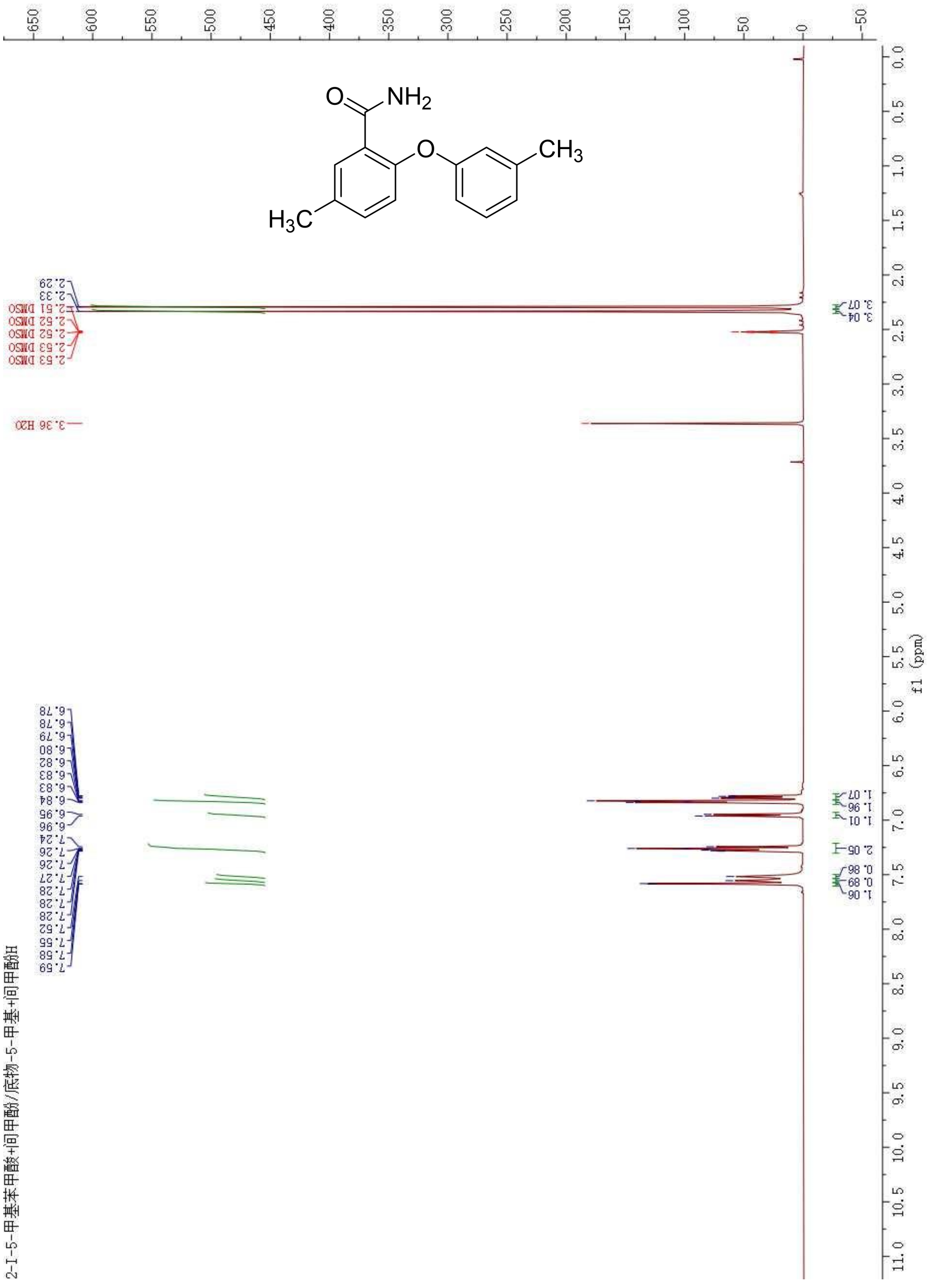

S11: ^{1}H-NMR of **14f**

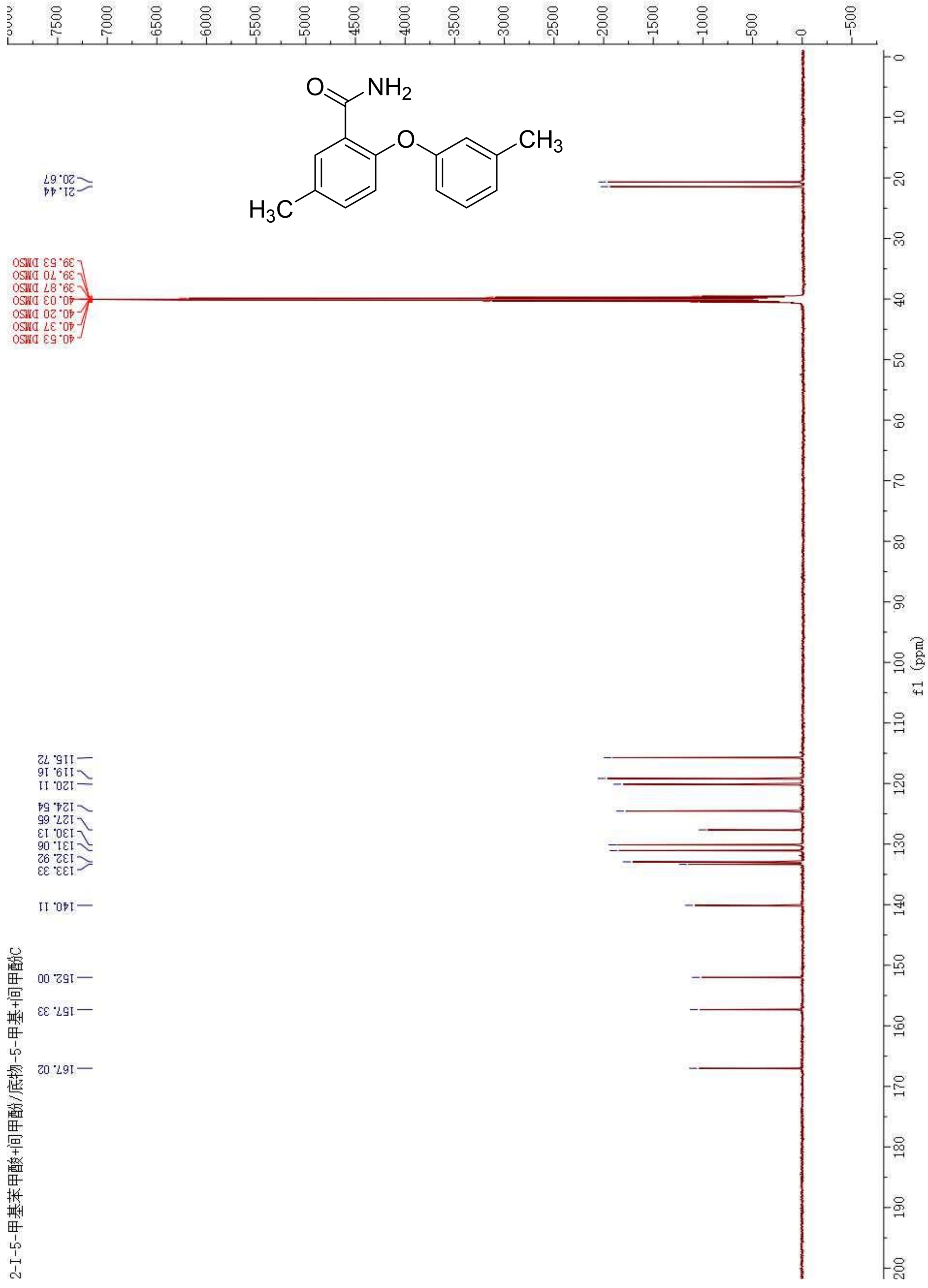

S$_{12}$: ^{13}C-NMR of **14f**

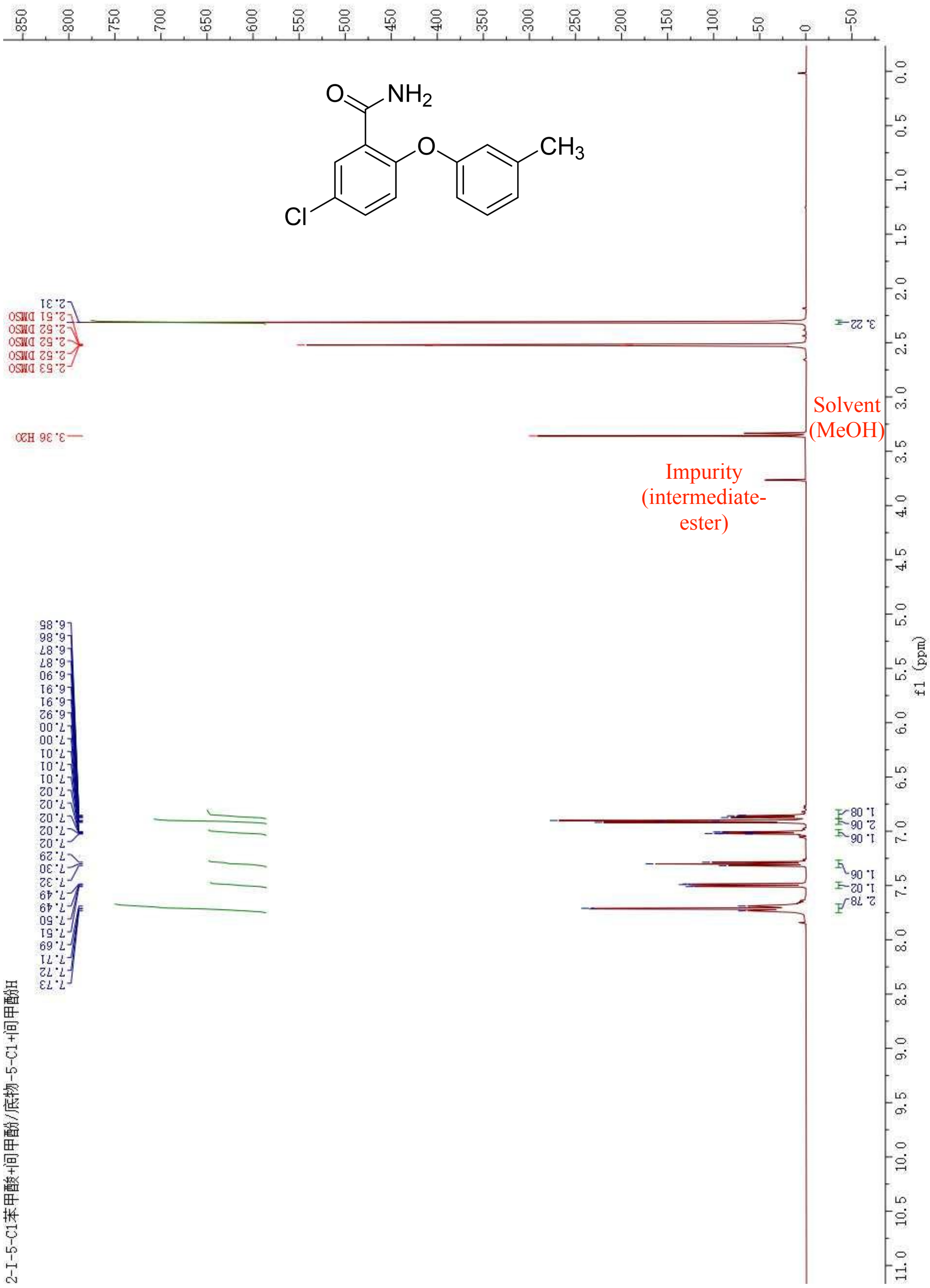

S13: ^{1}H-NMR of **14g**

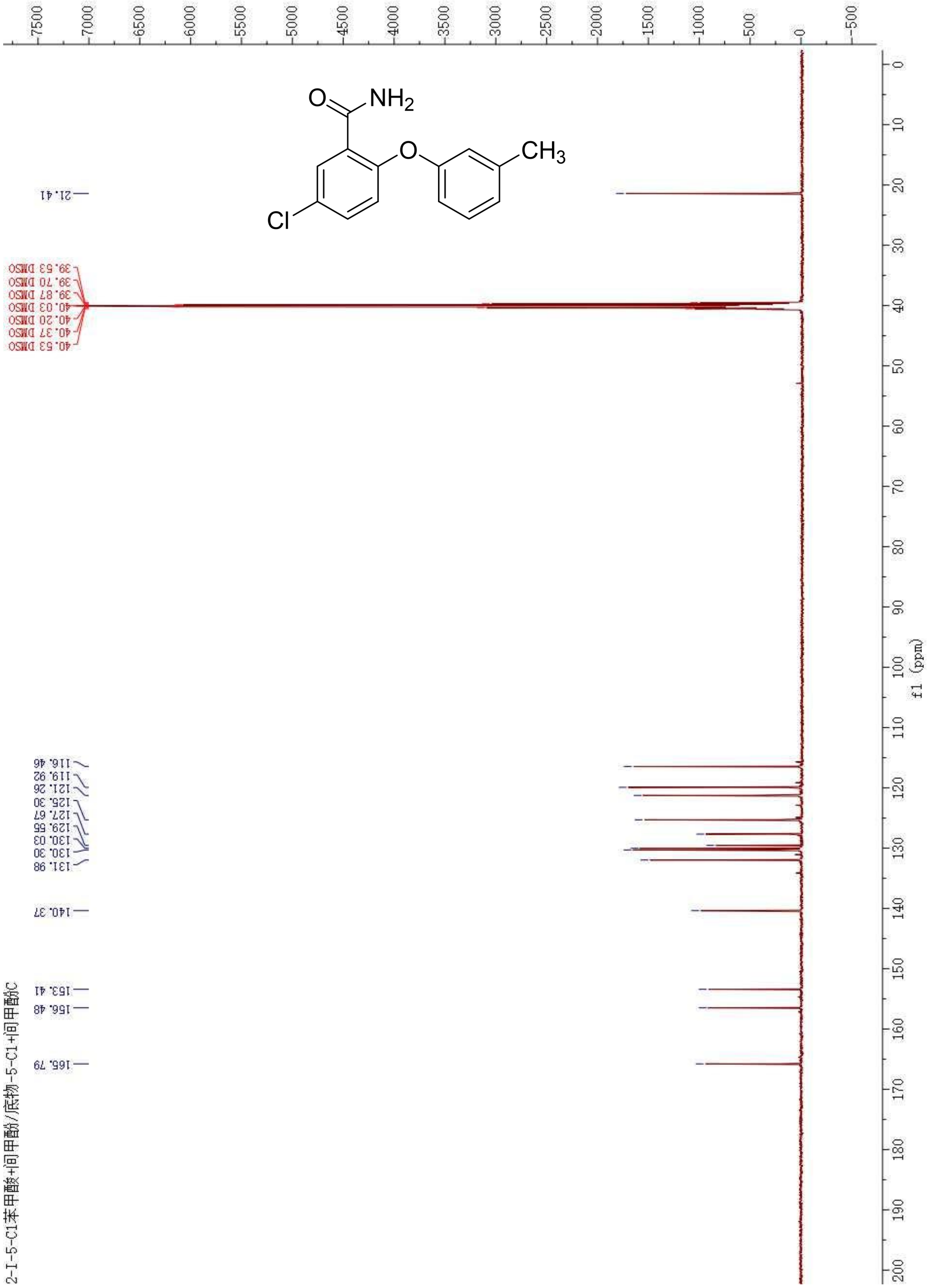

S14: ^{13}C-NMR of **14g**

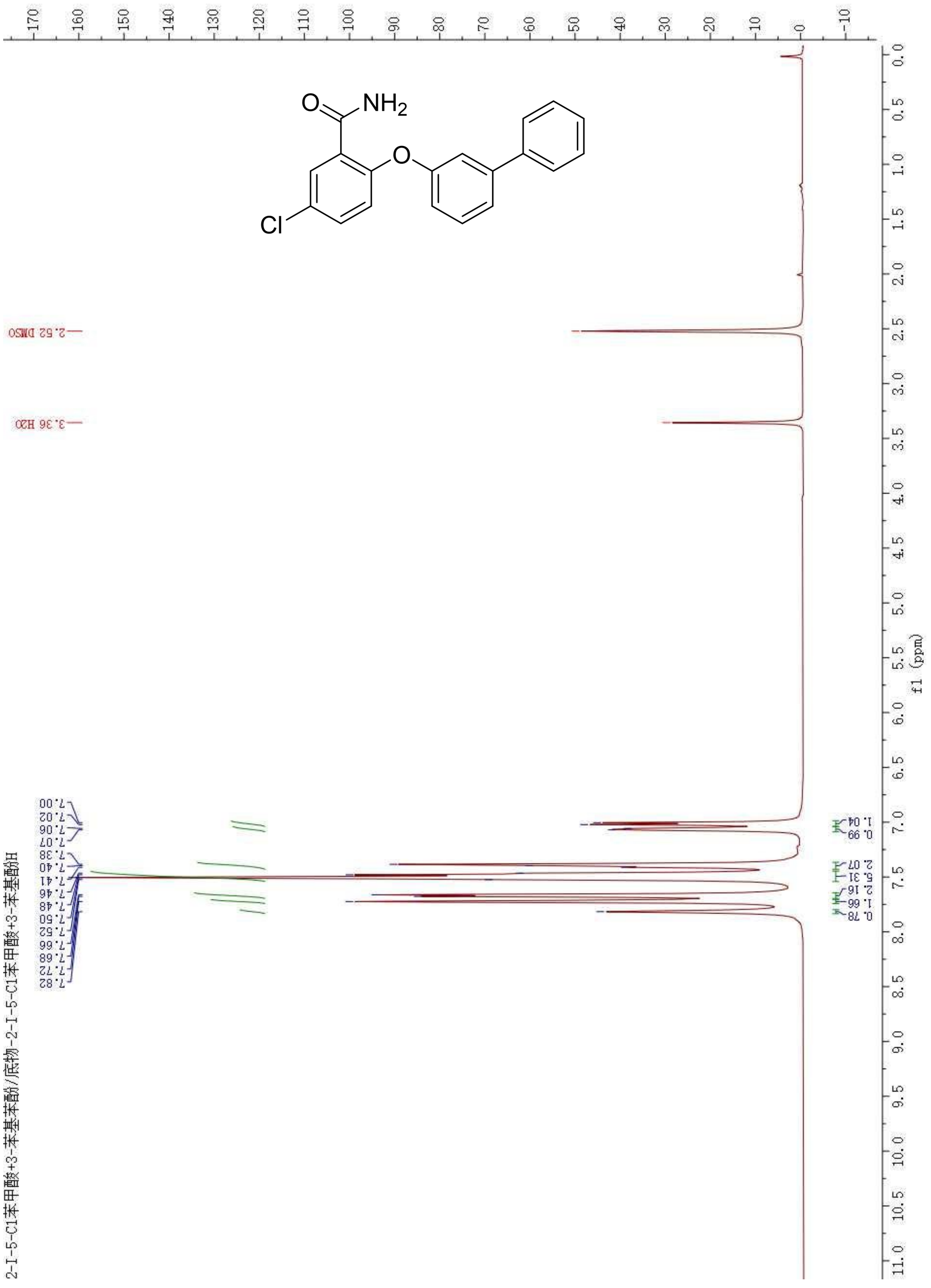

S$_{15}$: ^{1}H-NMR of **14h**

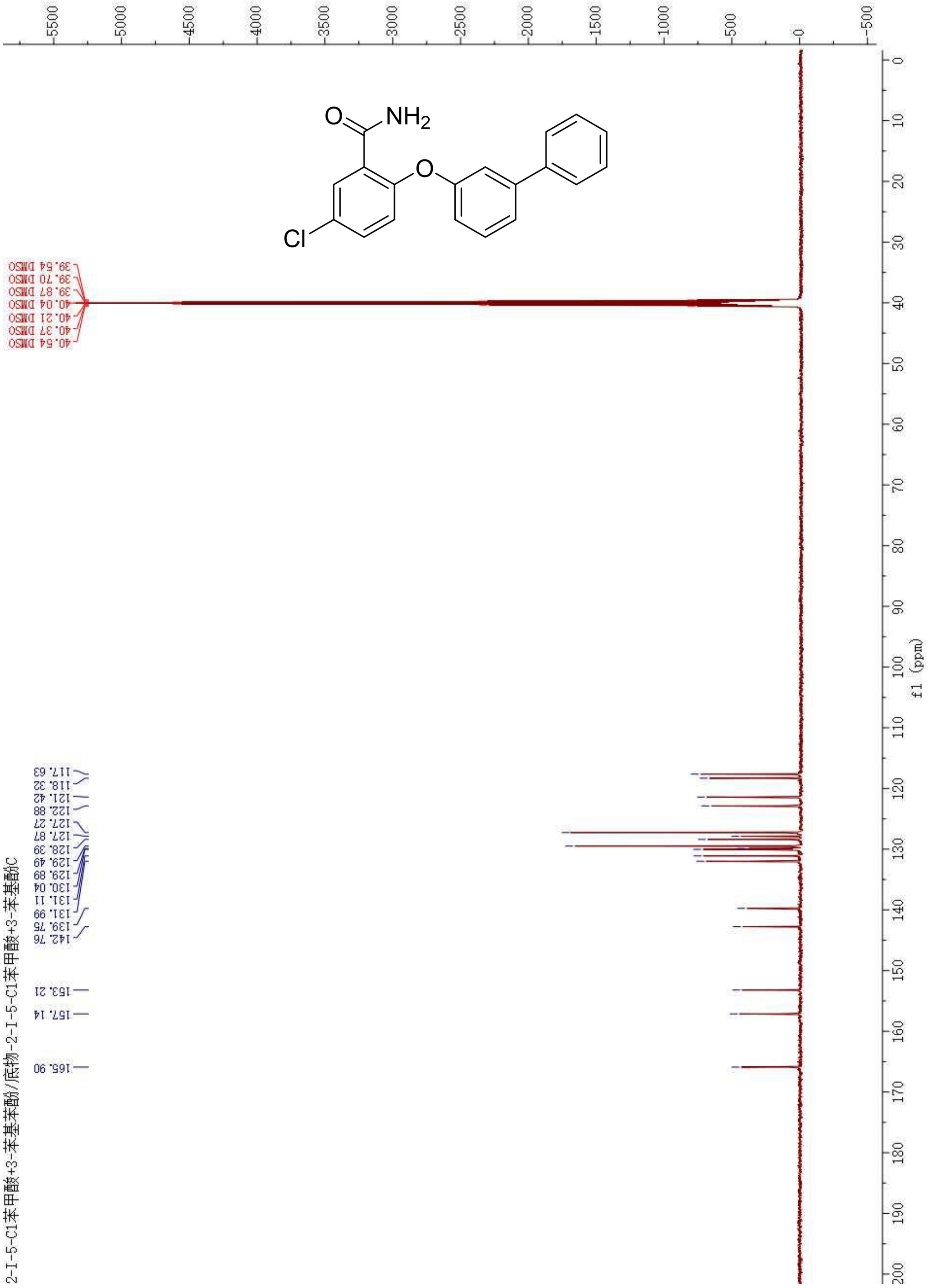

S16: ^{13}C-NMR of **14h**

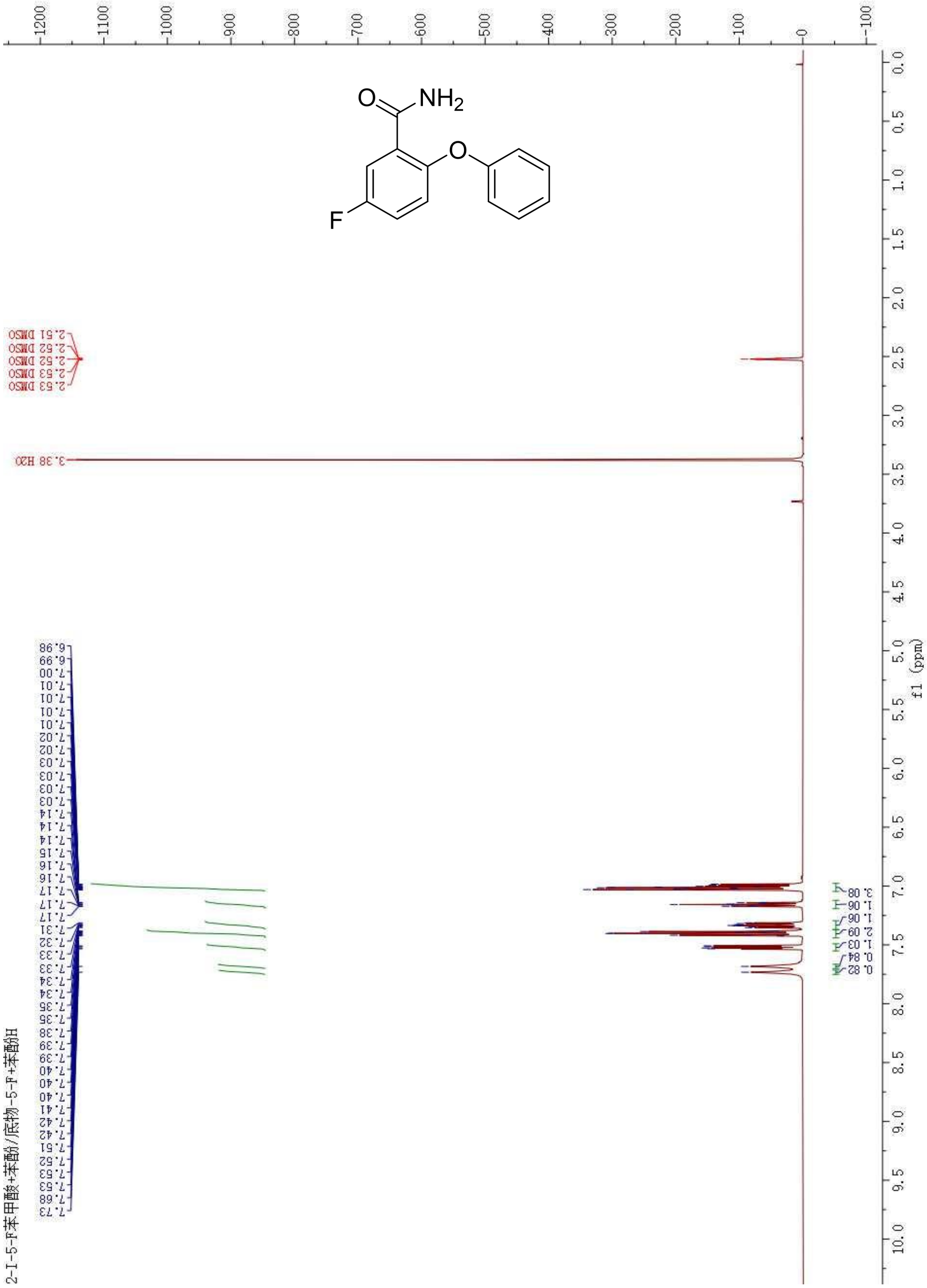

S17: ^{1}H-NMR of **14i**

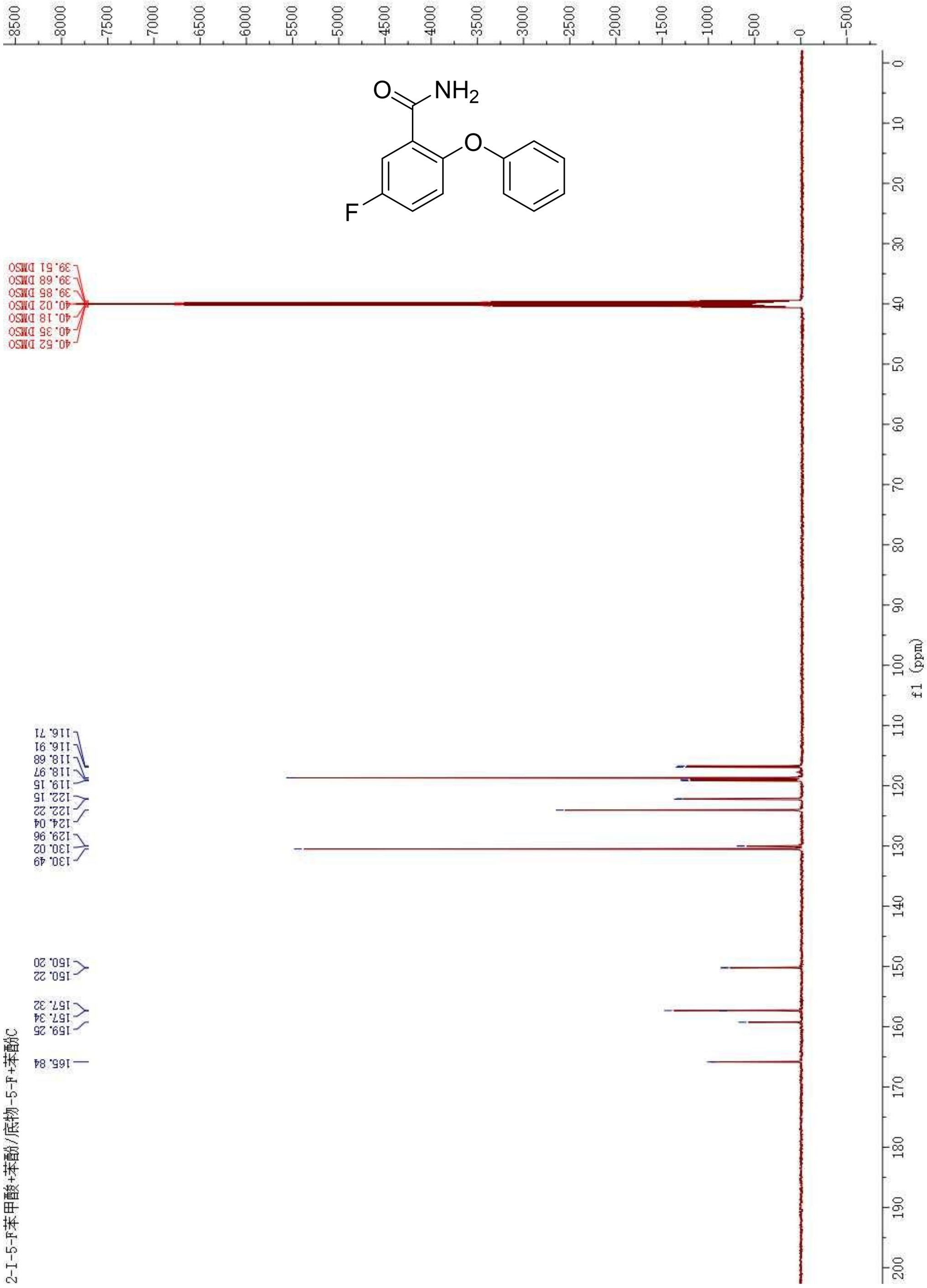

S18: ^{13}C-NMR of **14i**

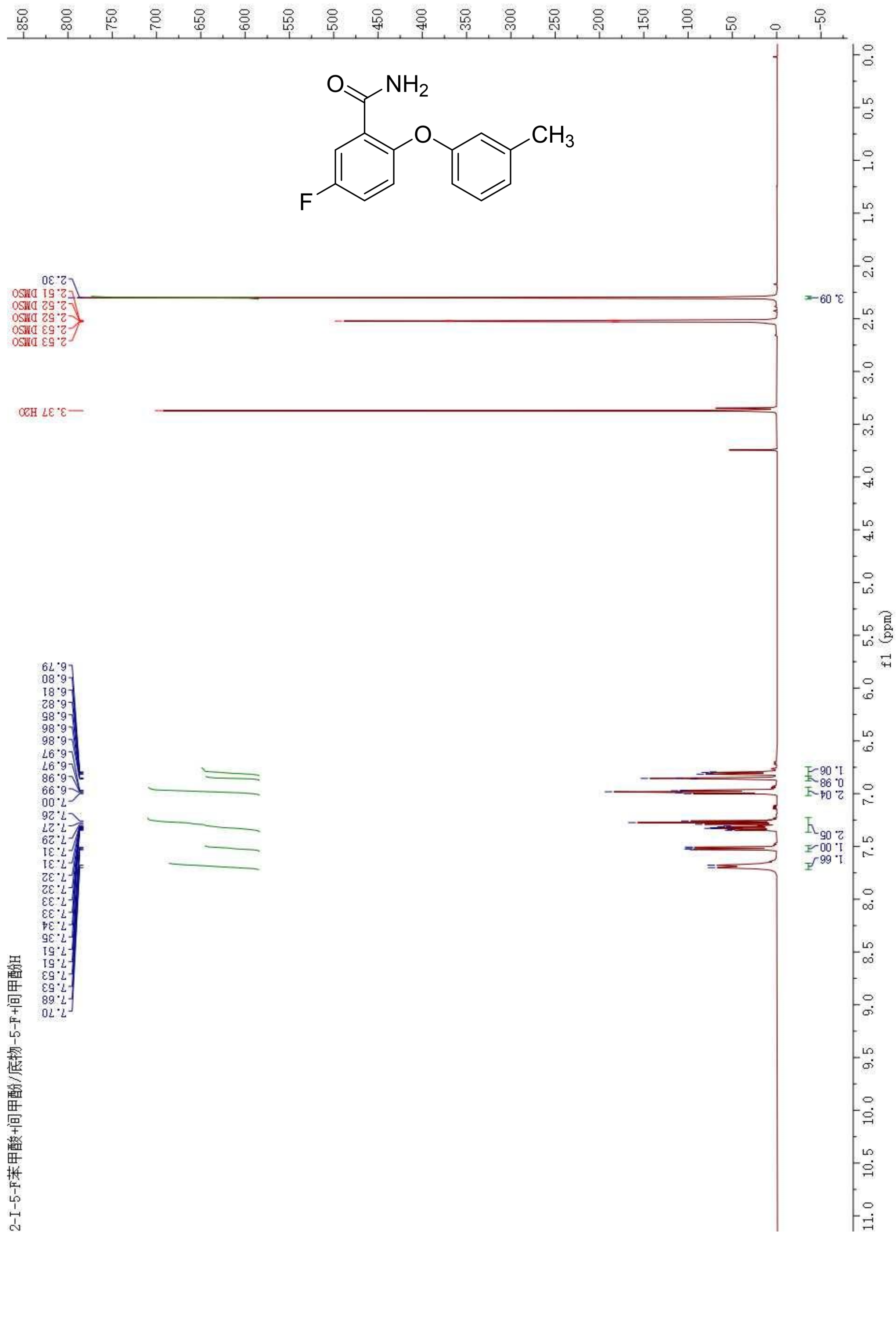

S19: ^{1}H-NMR of **14j**

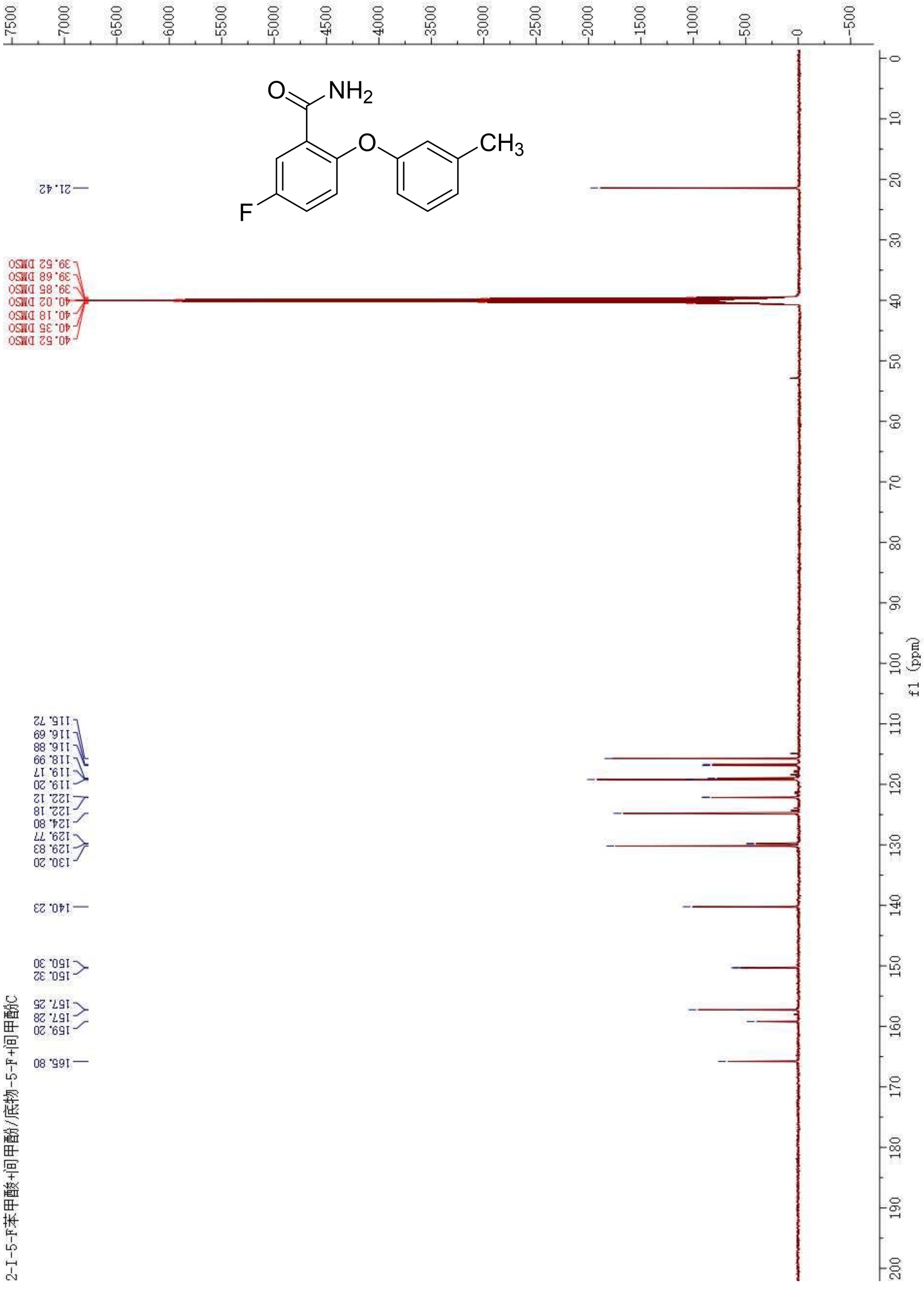

S20: ^{13}C-NMR of **14j**

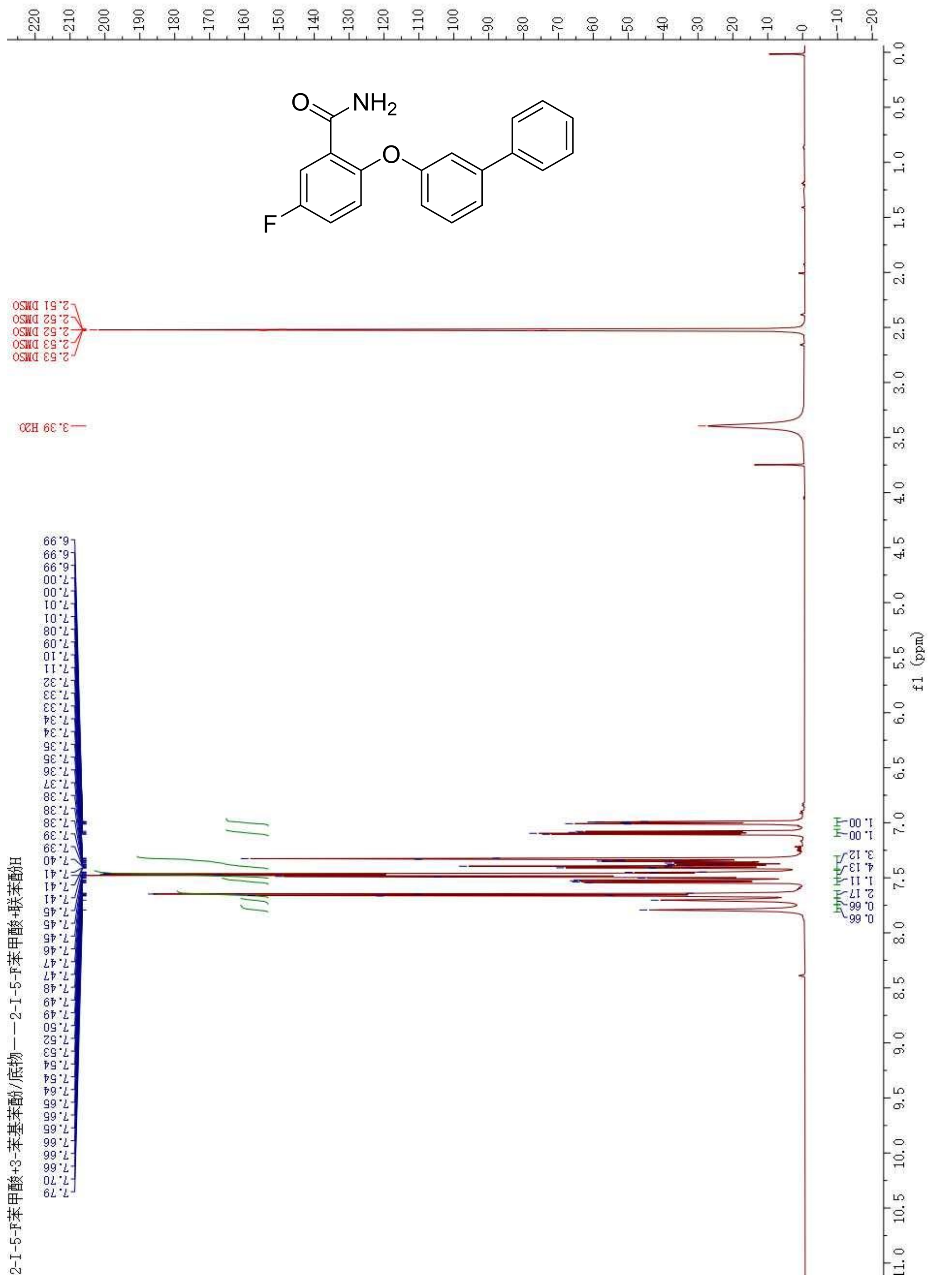

S_{21}: ^{1}H-NMR of **14k**

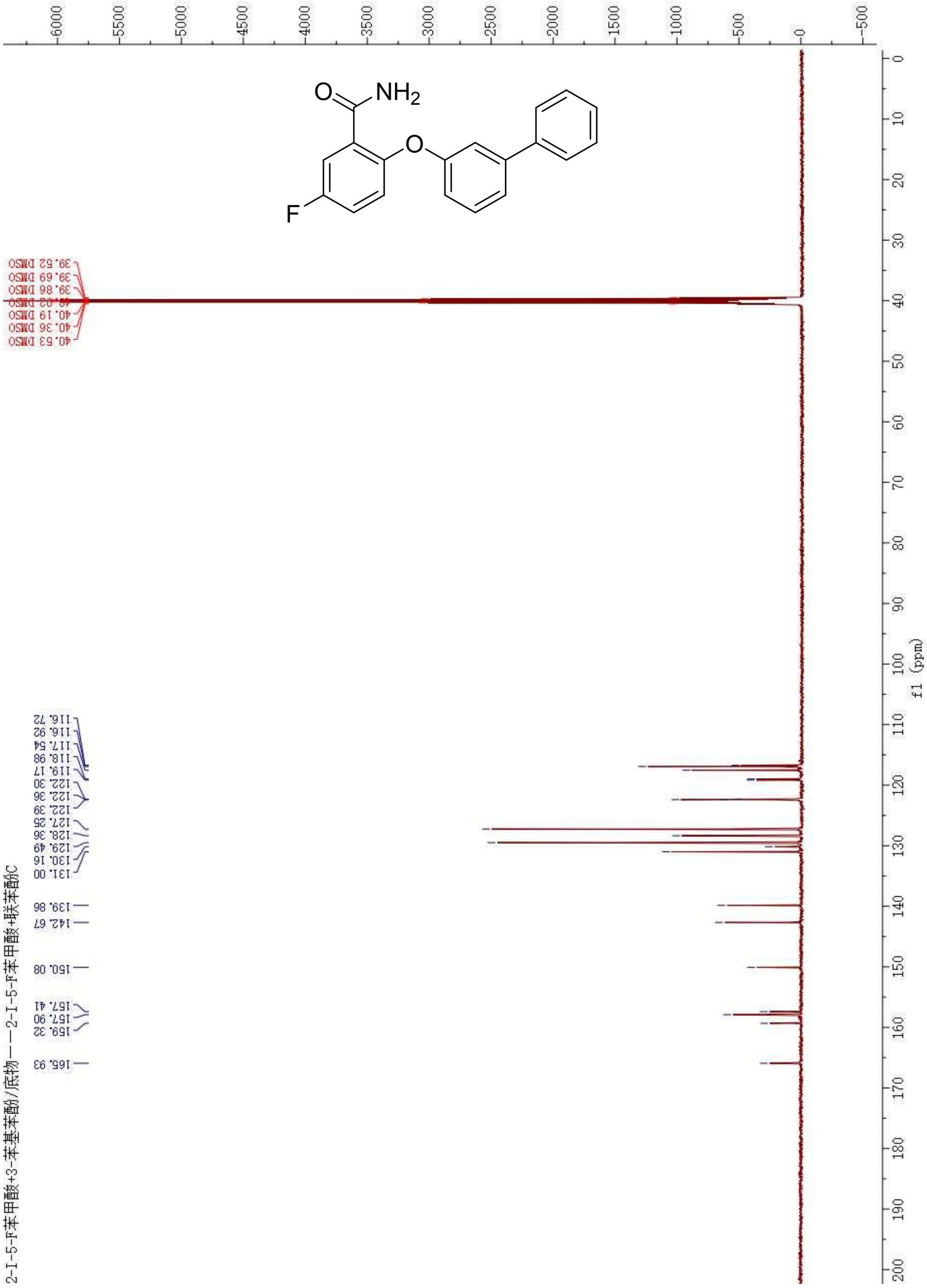

S$_{22}$: ^{13}C-NMR of **14k**

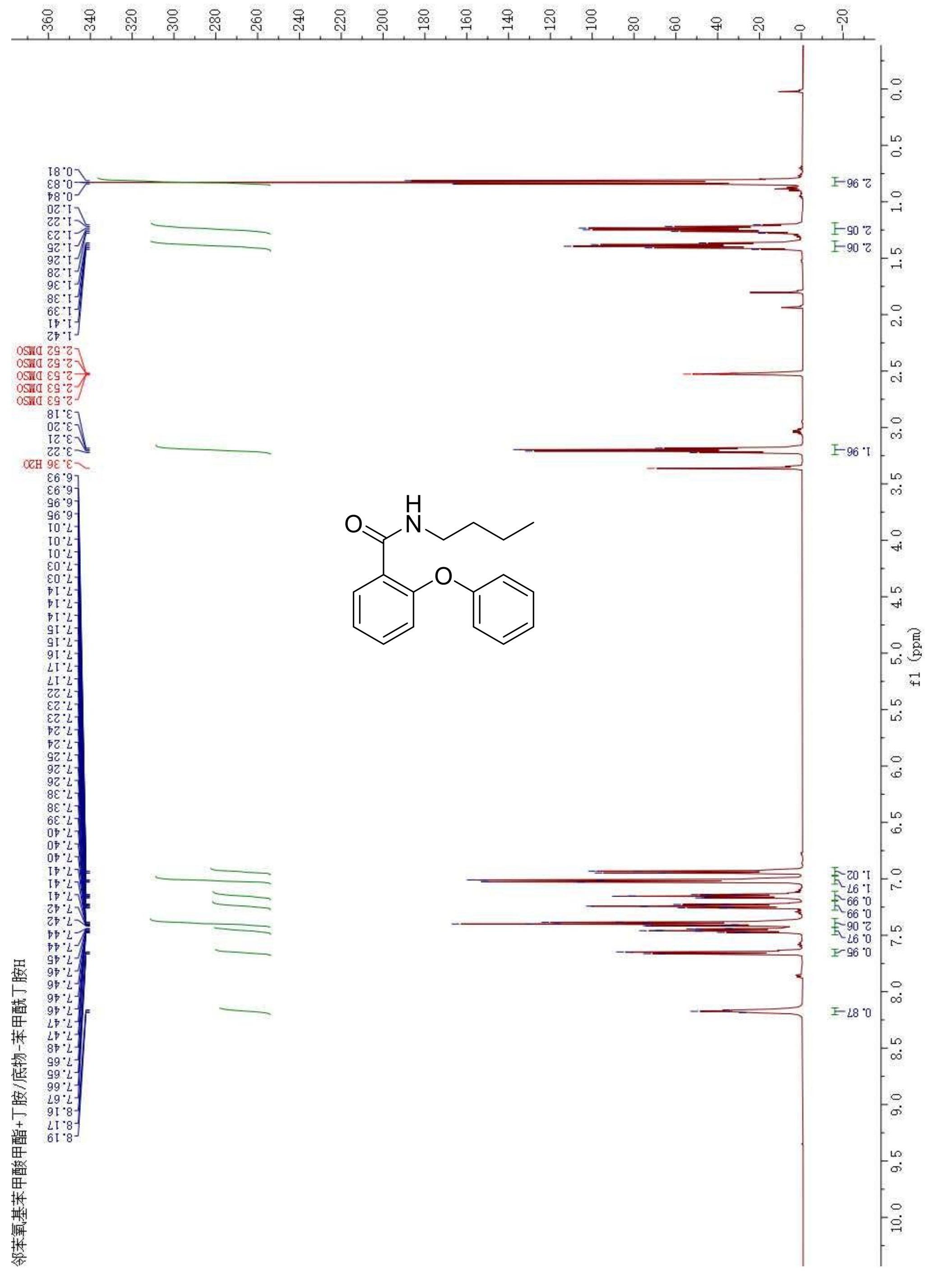

S$_{23}$: ^{1}H-NMR of **14l**

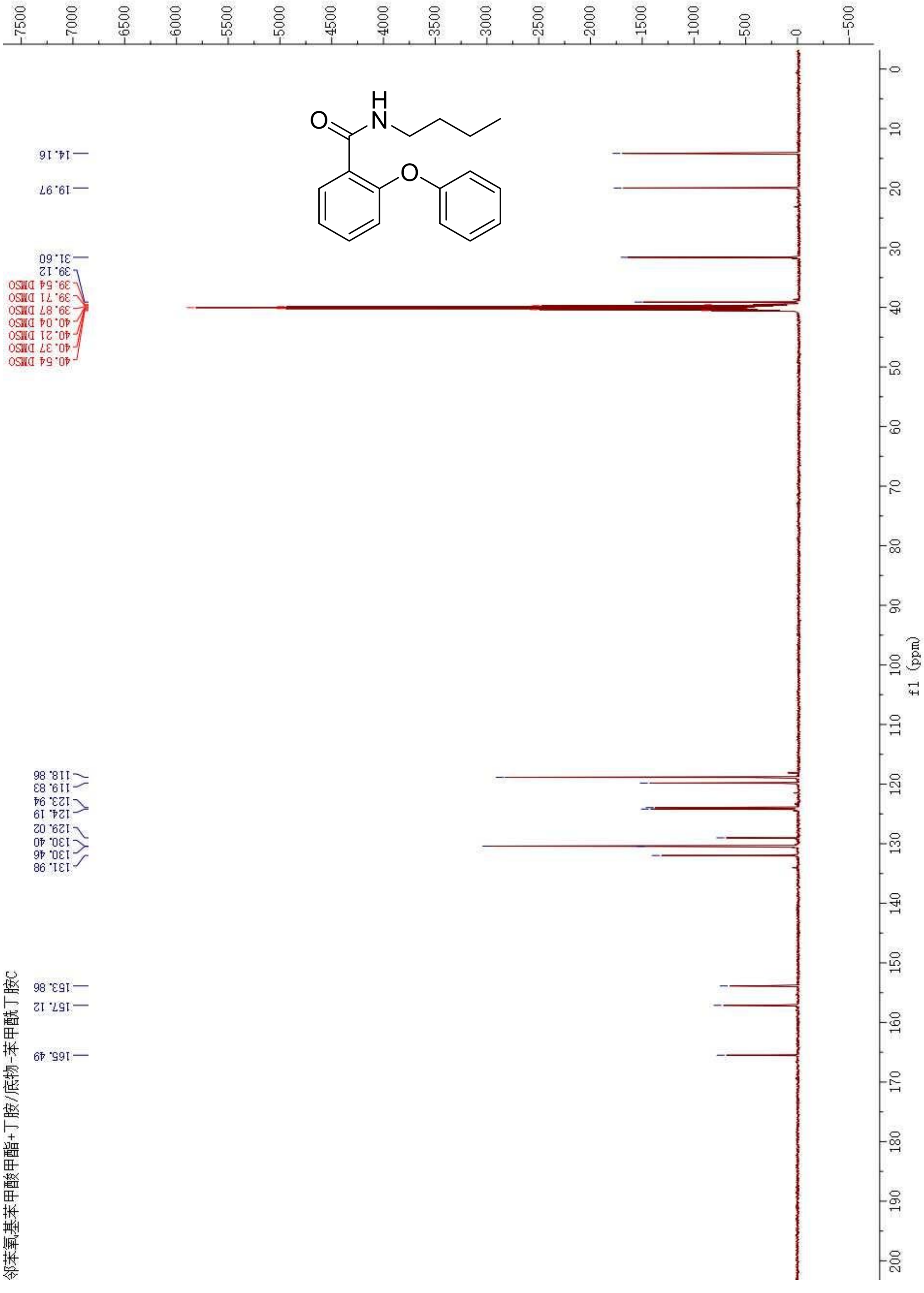

S24: ^{13}C-NMR of **14l**

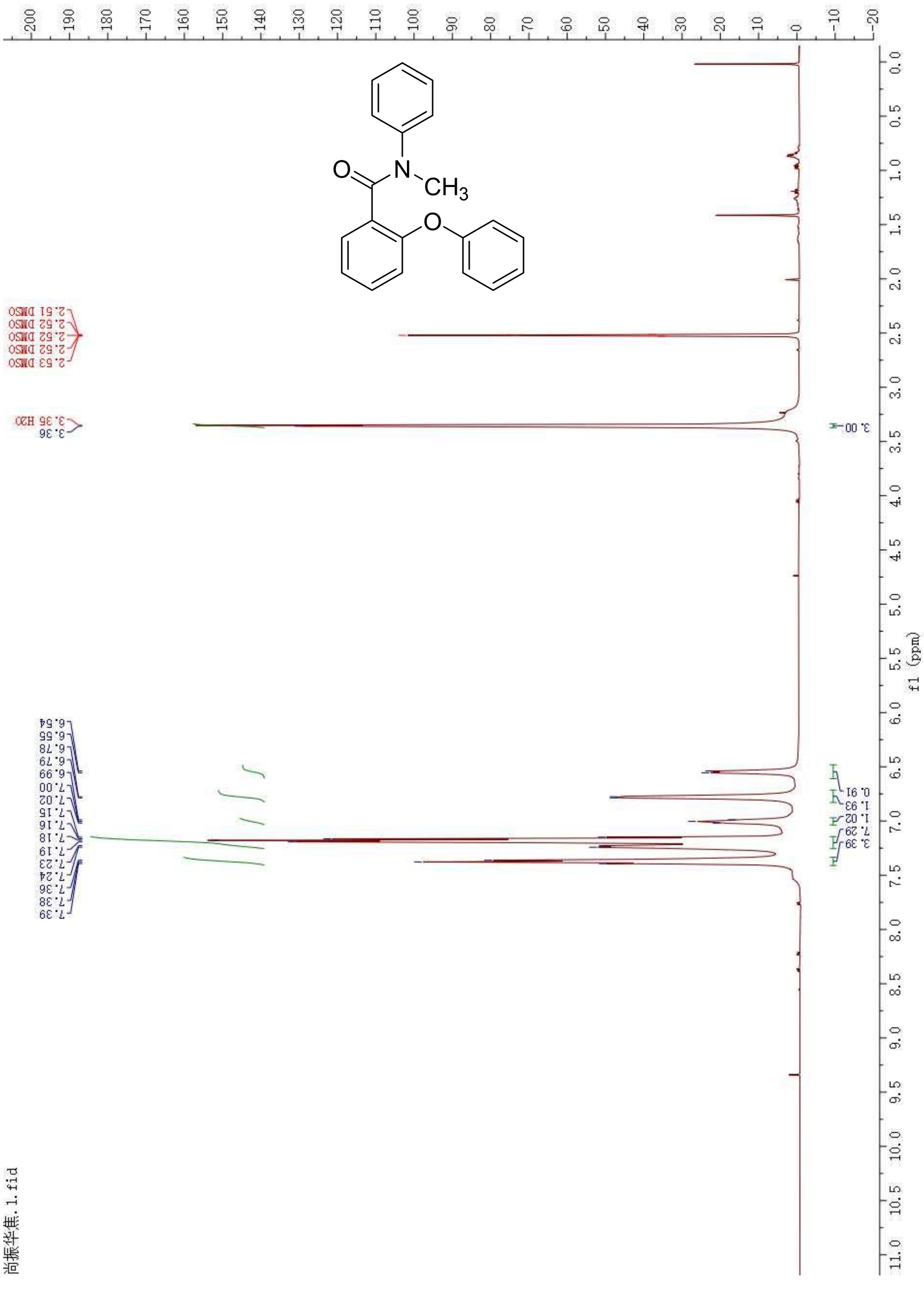

S25: ^{1}H-NMR of **14m**

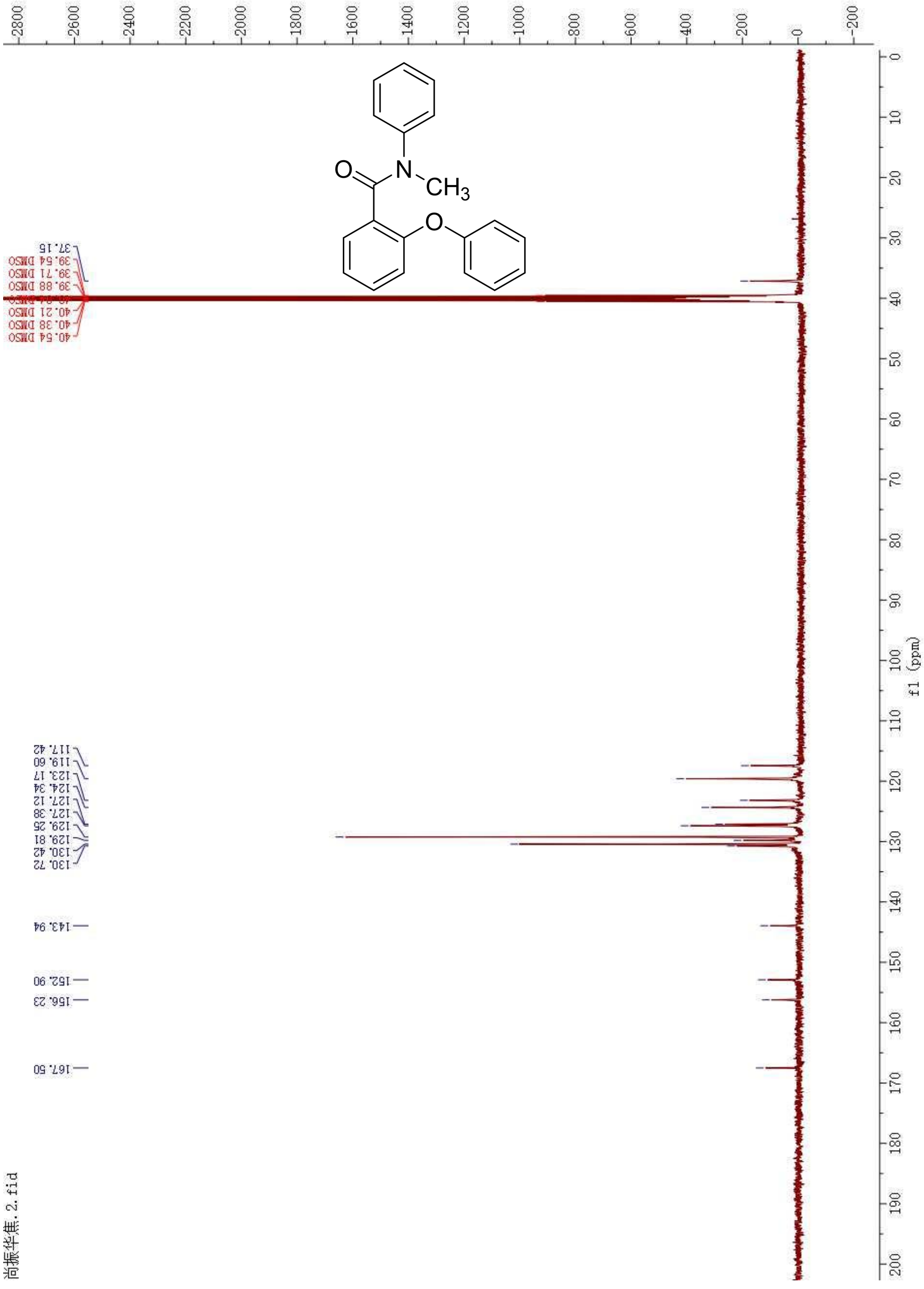

S$_{26}$: ^{13}C-NMR of **14m**

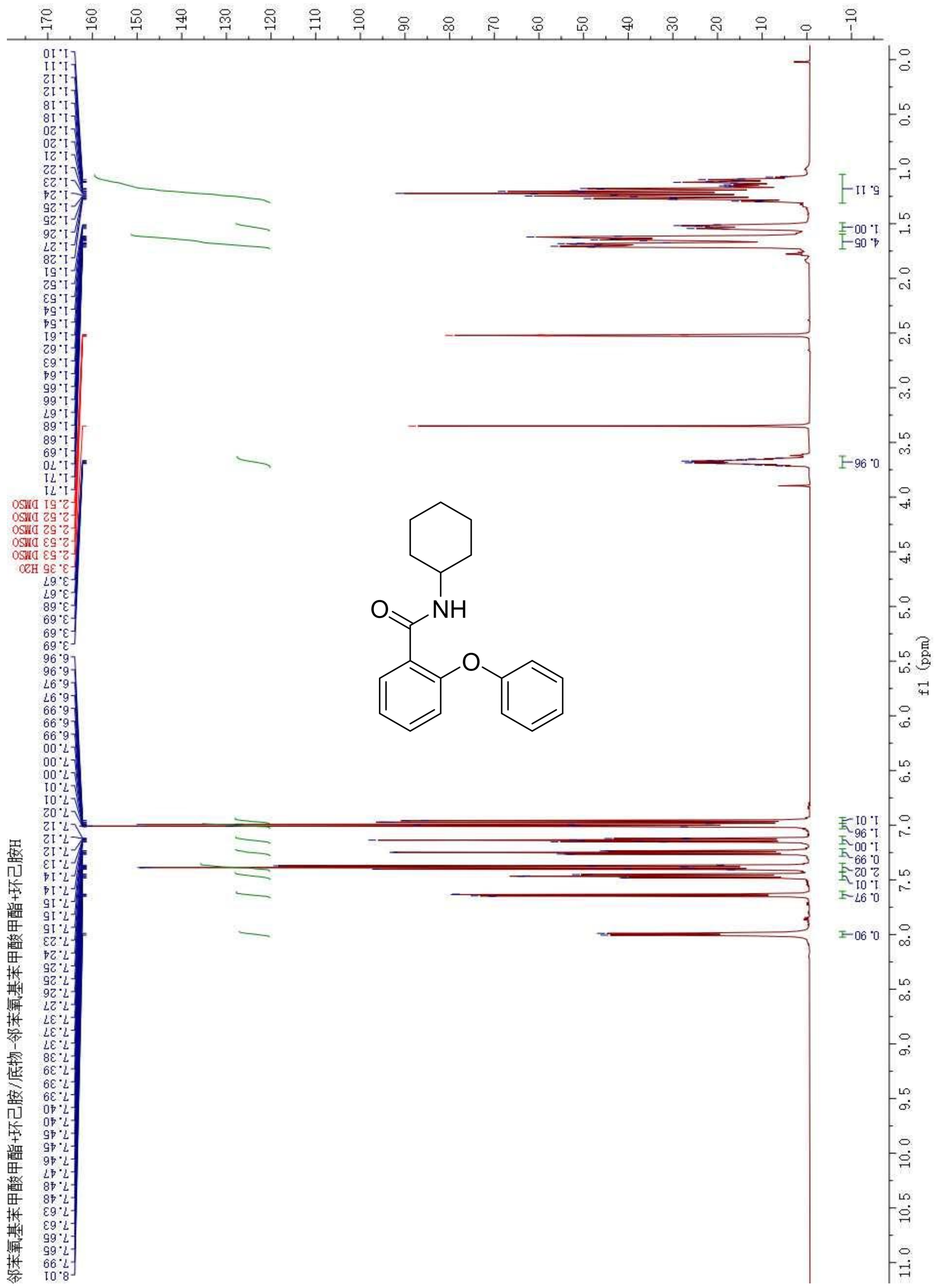

S_{27}: ^{1}H-NMR of **14n**

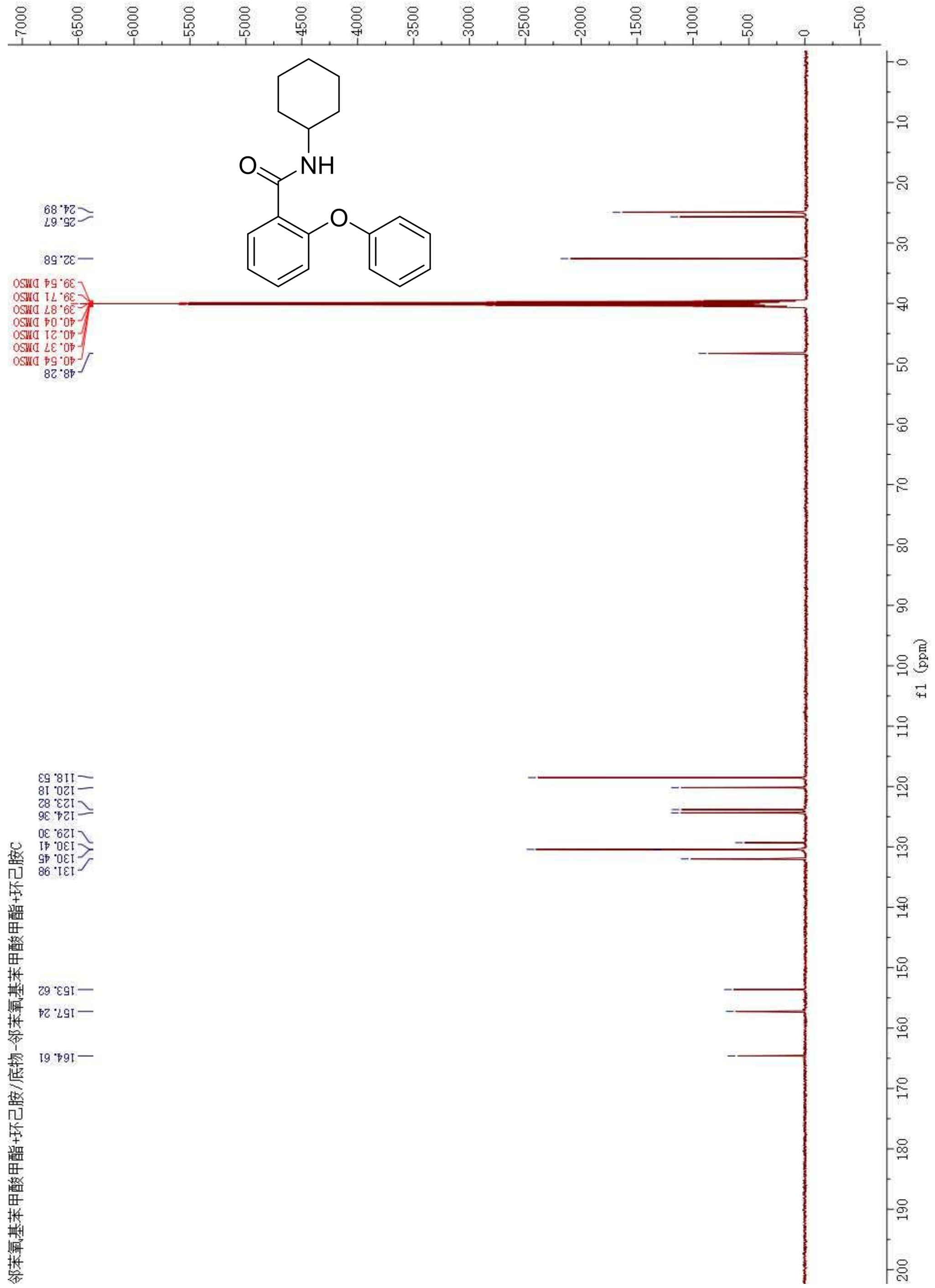

S28: ^{13}C-NMR of **14n**

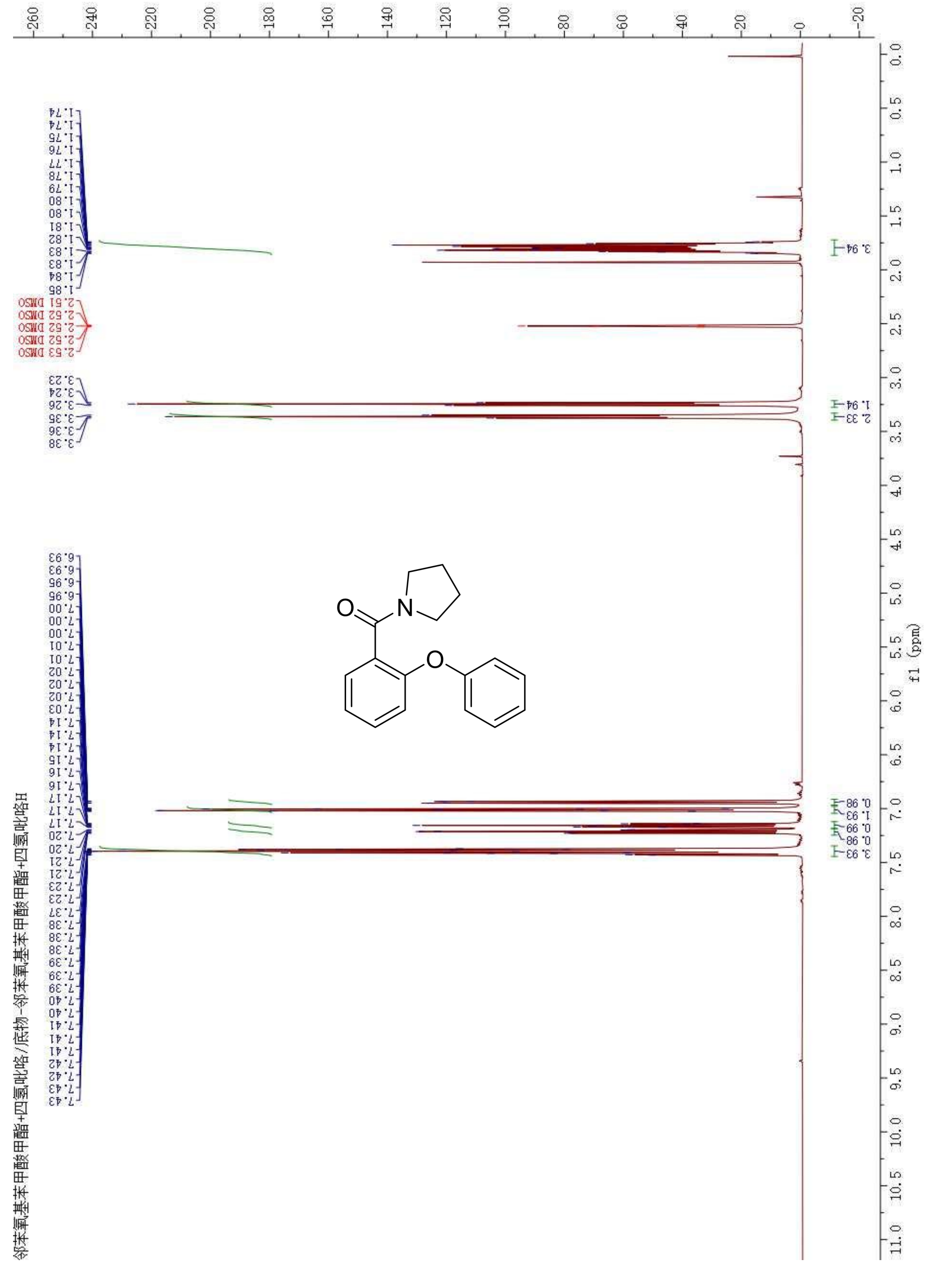

S29: ^{1}H-NMR of **14o**

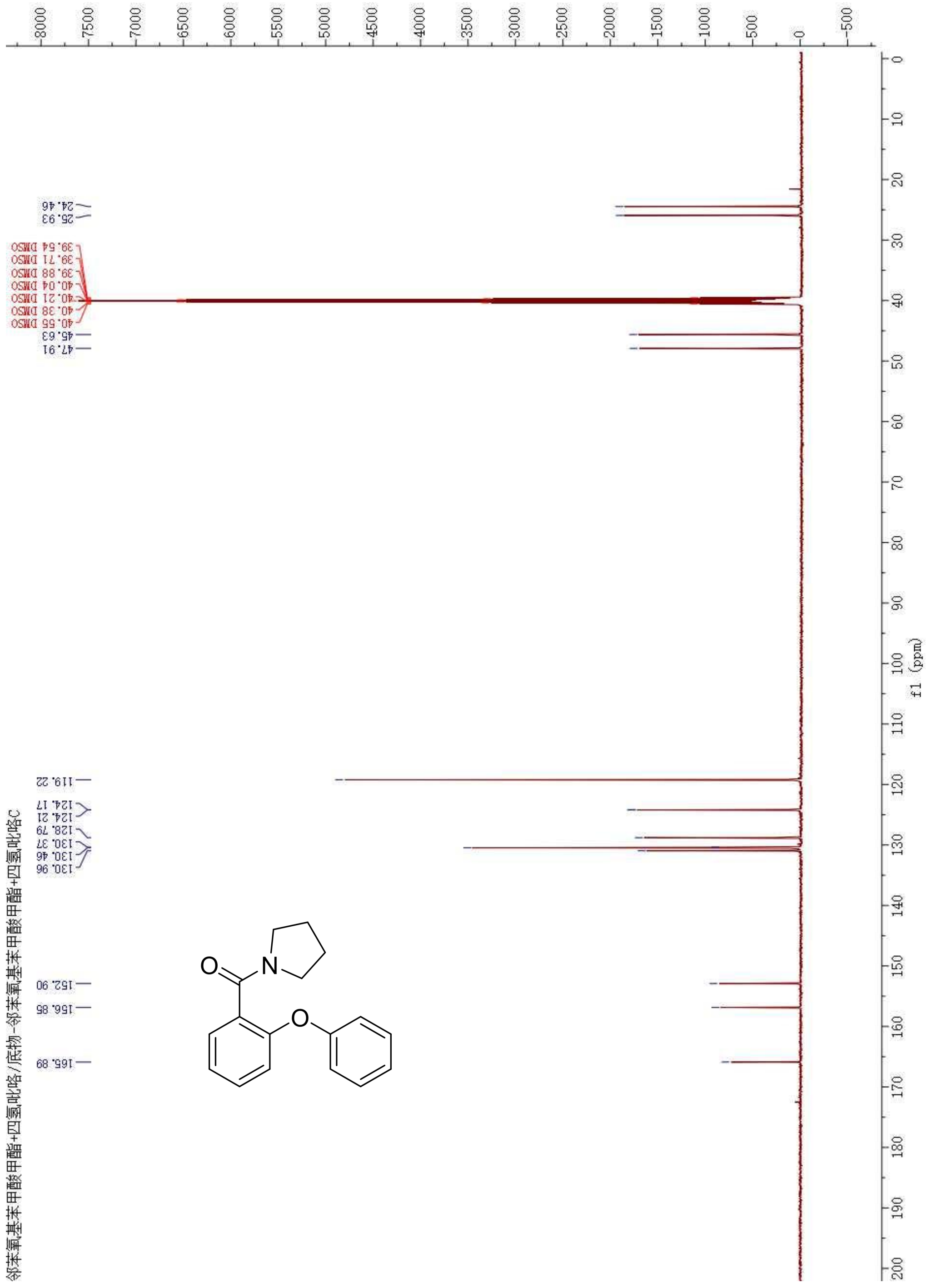

S30: ^{13}C-NMR of **14o**

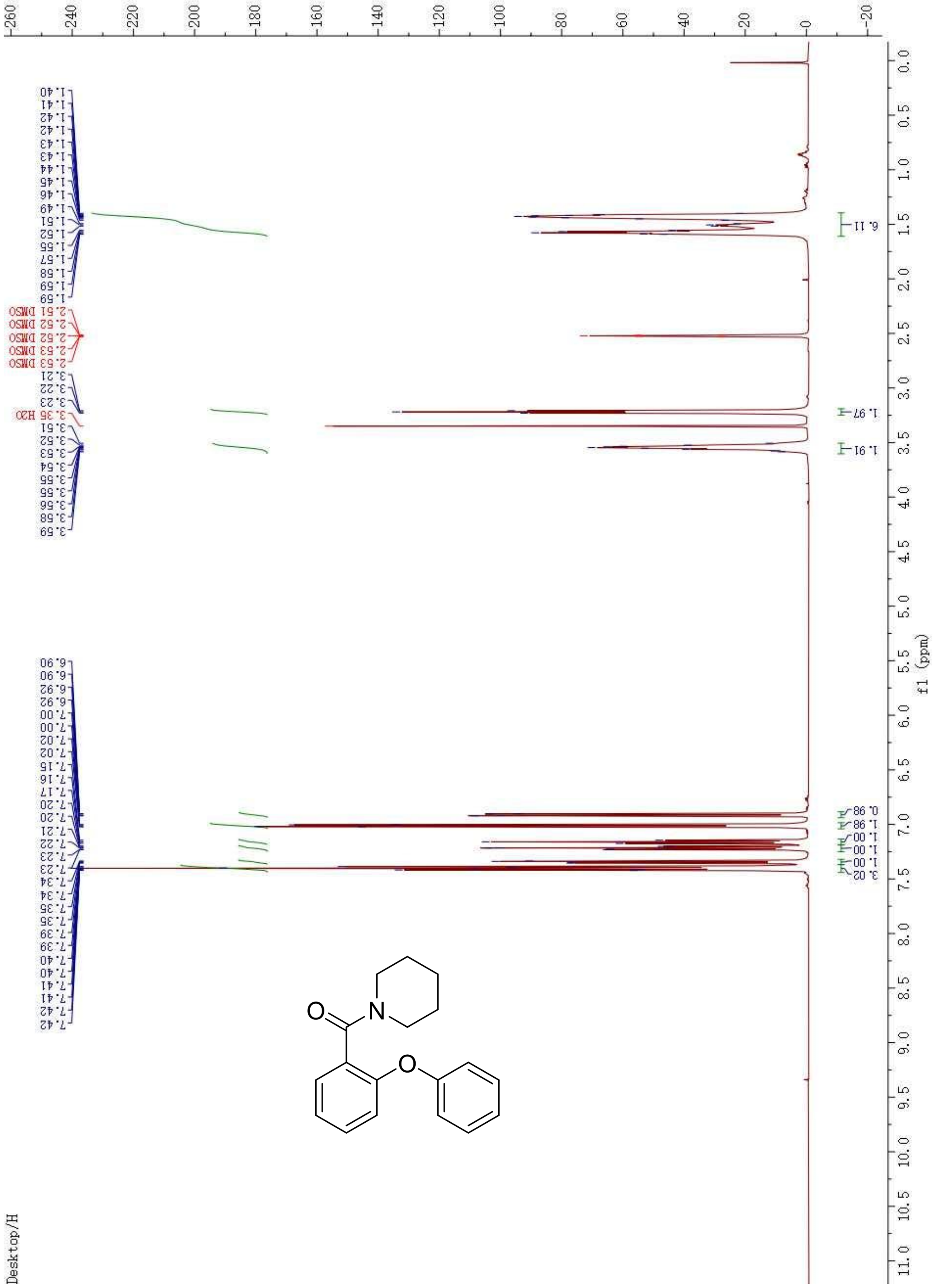

S_{31}: ^{1}H-NMR of **14p**

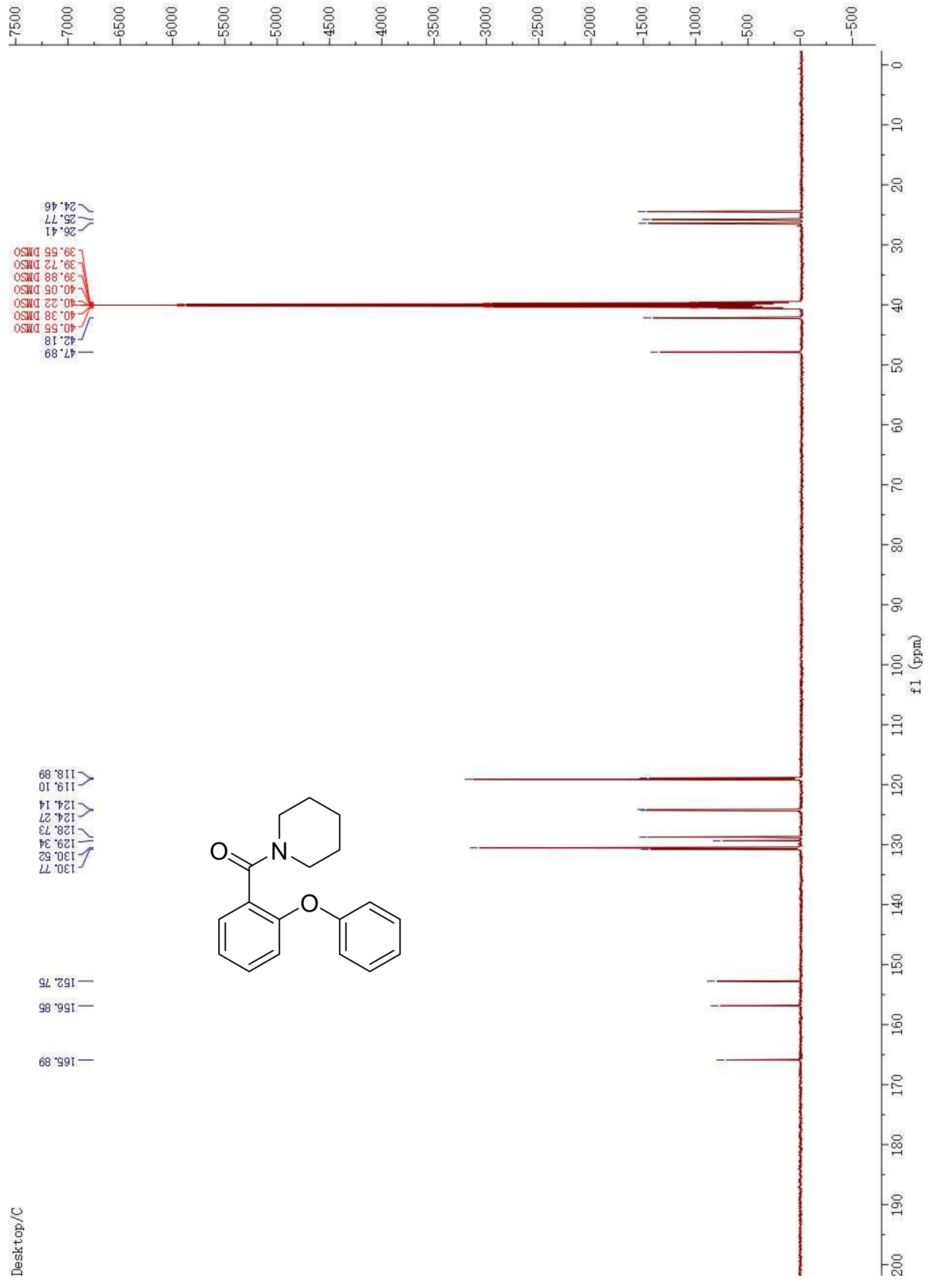

S32: ^{13}C-NMR of **14p**

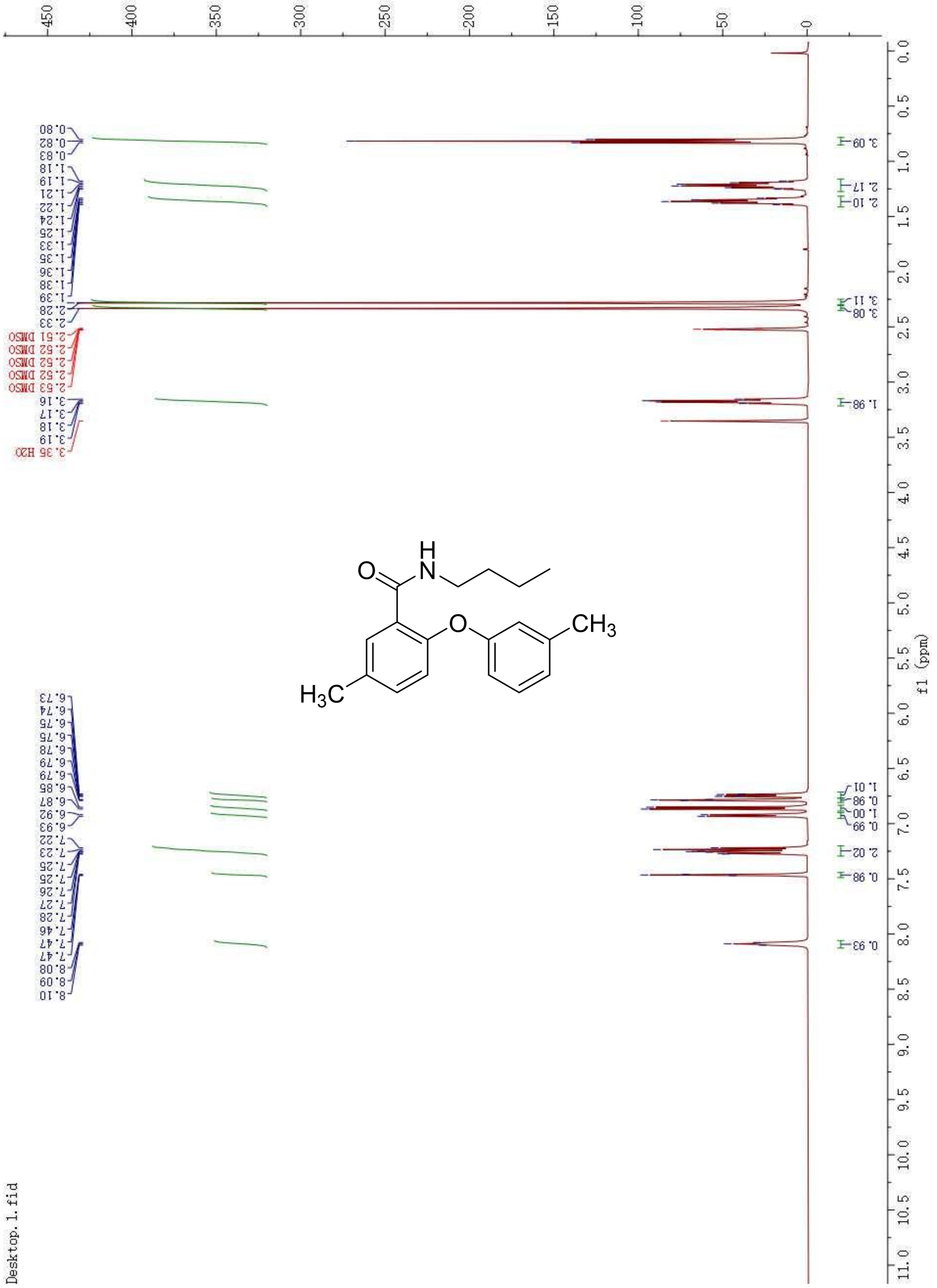

S$_{33}$: ^{1}H-NMR of **14q**

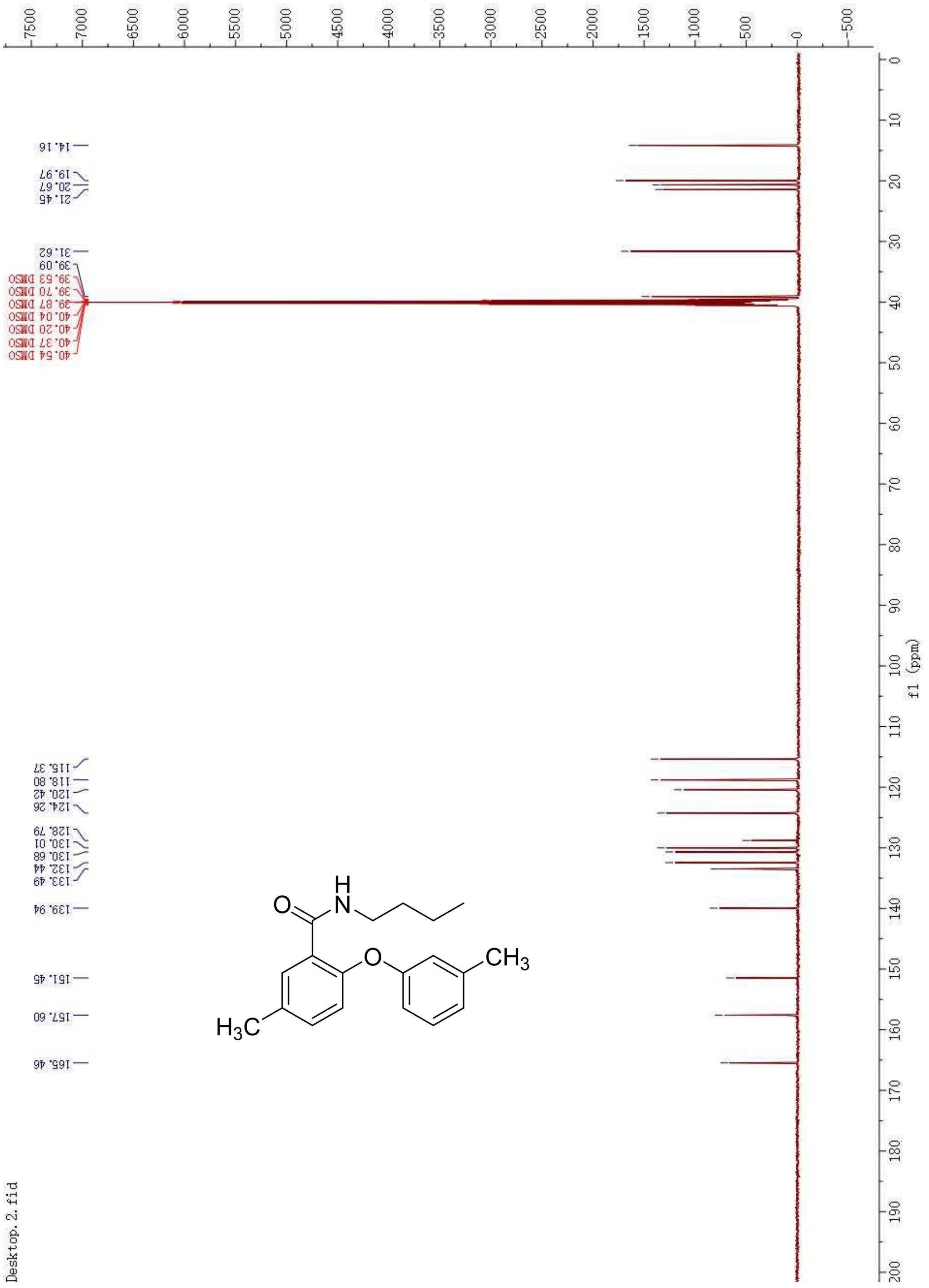

S34: ^{13}C-NMR of **14q**

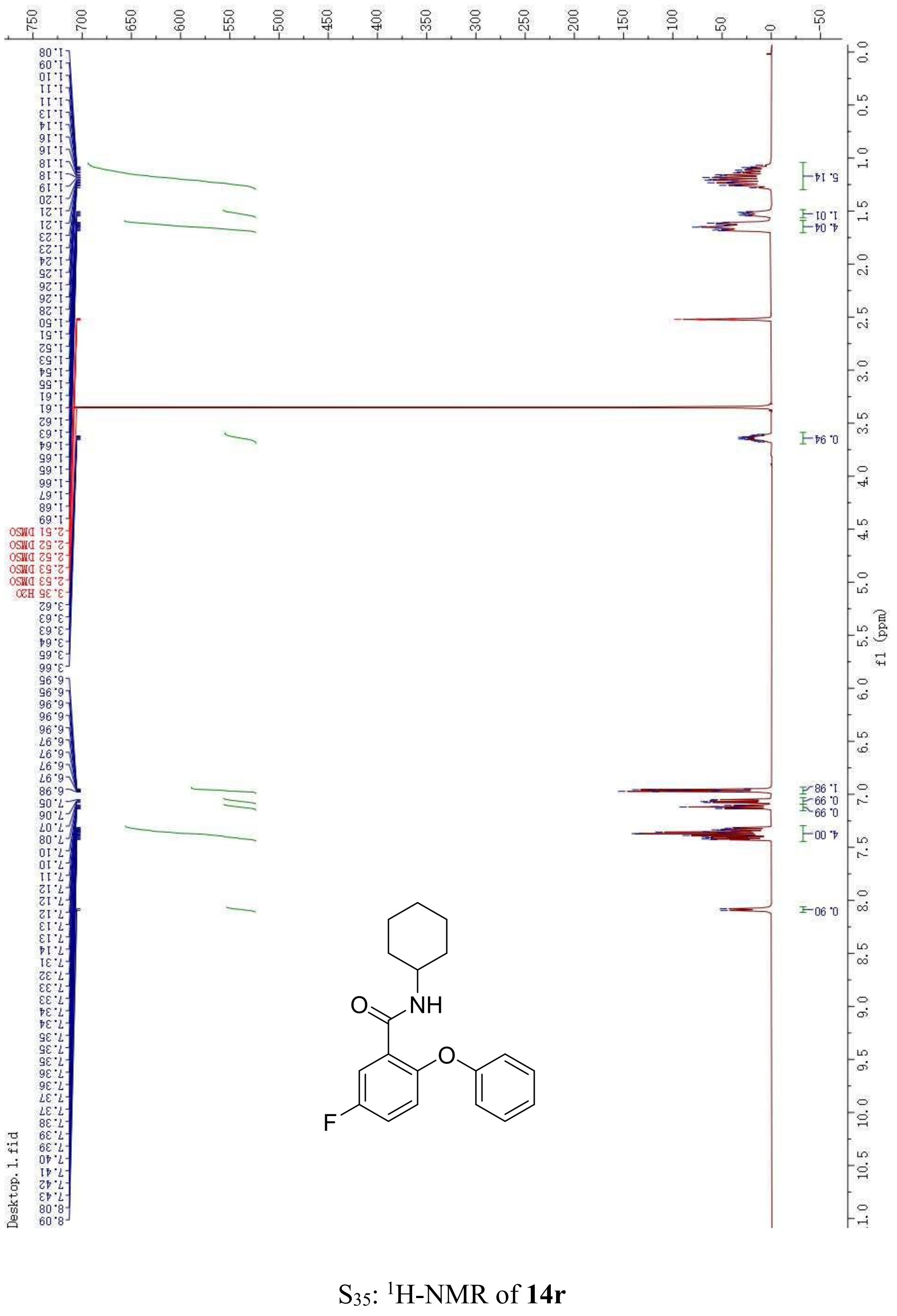

S$_{35}$: ^{1}H-NMR of **14r**

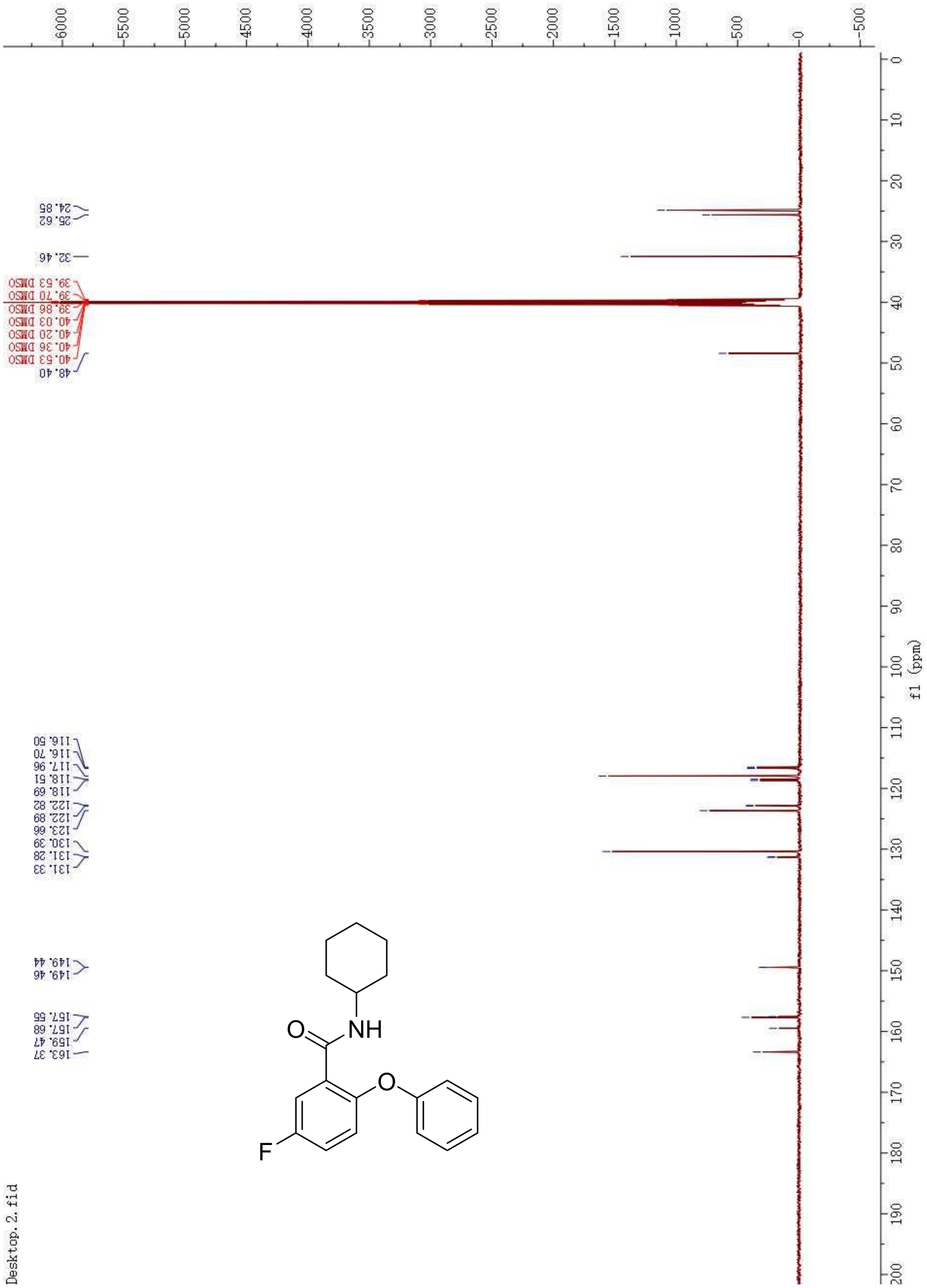

S$_{36}$: ^{13}C-NMR of **14r**

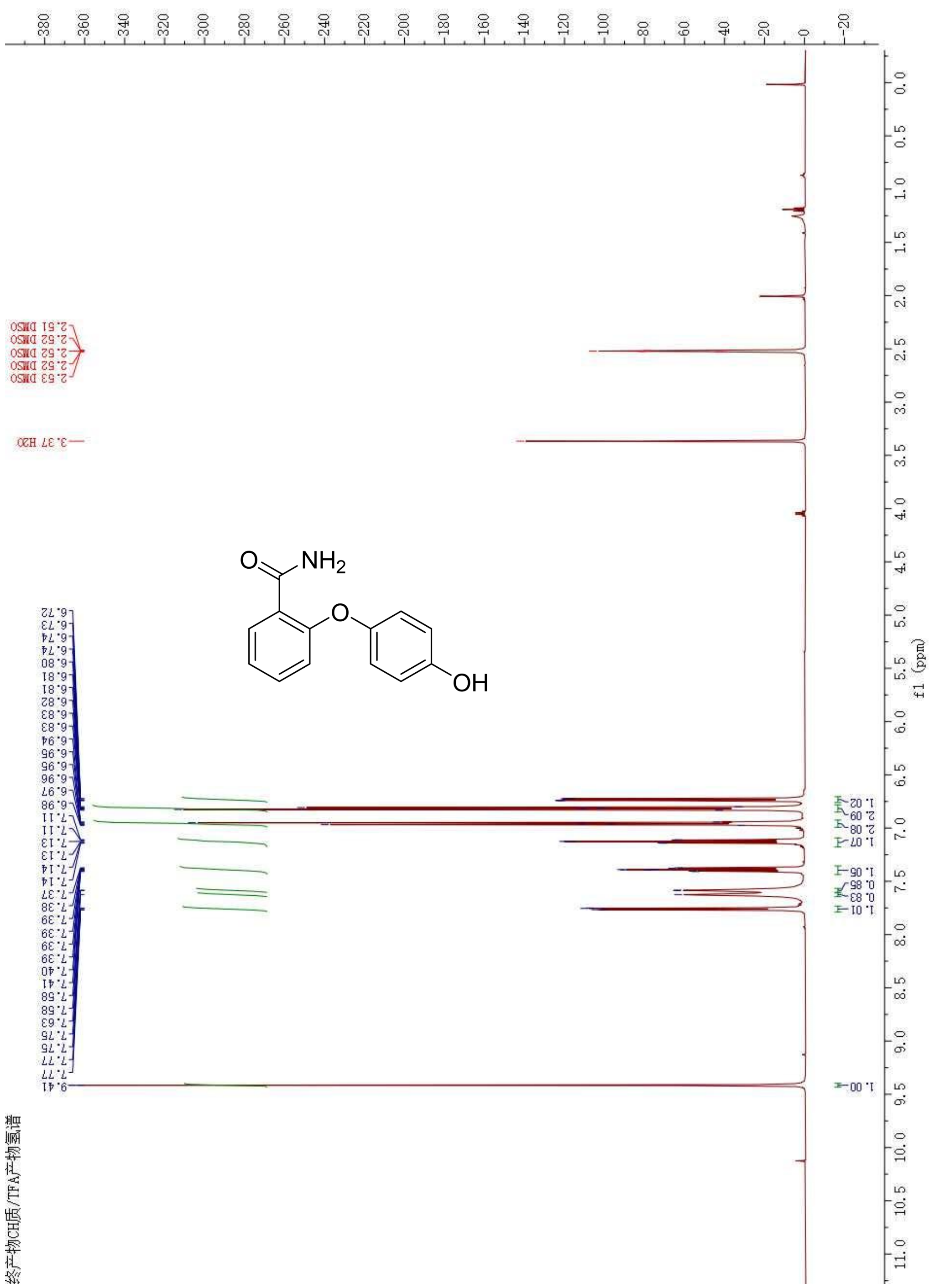

S37: ^{1}H-NMR of **15a**

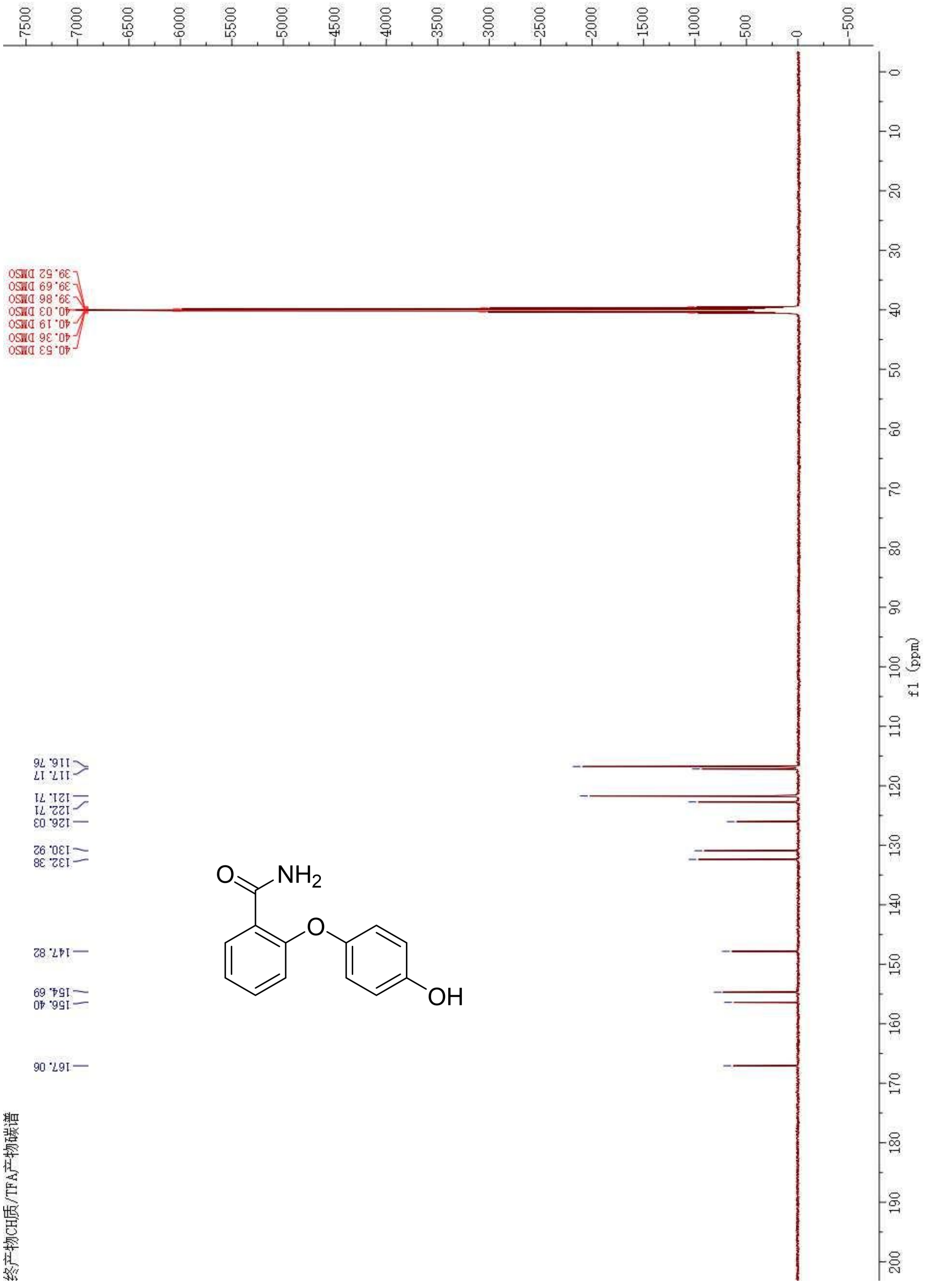

S38: ^{13}C-NMR of **15a**

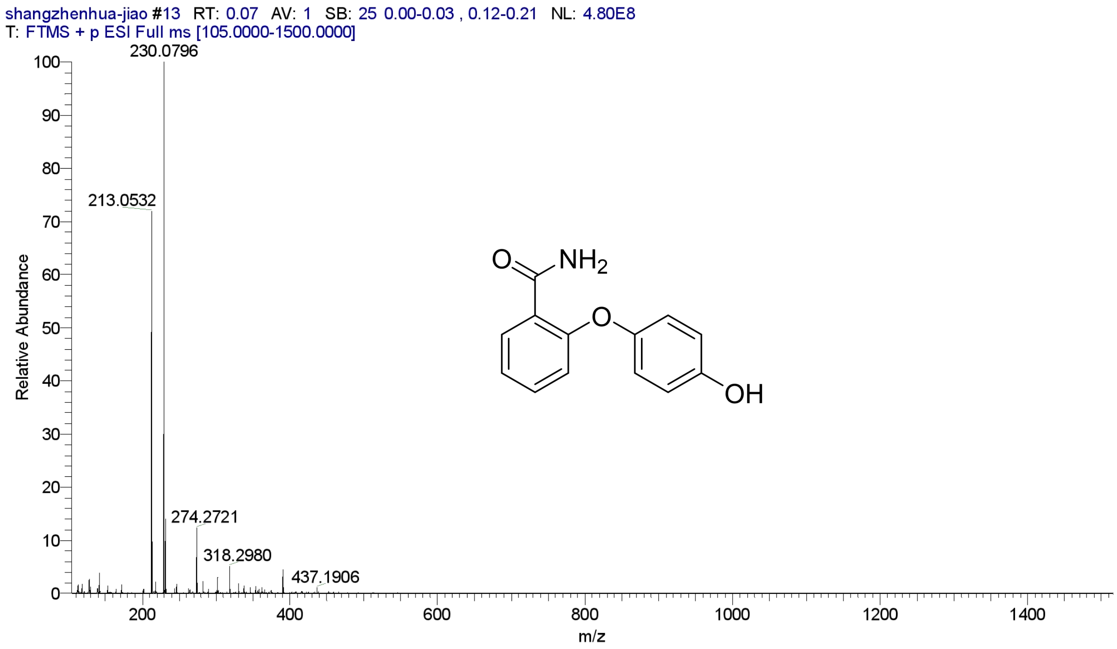
shangzhenhua-jiao #13 RT: 0.07 AV: 1 SB: 25 0.00-0.03 , 0.12-0.21 NL: 4.80E8
T: FTMS + p ESI Full ms [105.0000-1500.0000]
230.0796
213.0532
274.2721
318.2980
437.1906
Relative Abundance
m/z
O
NH2
O
OH

S39:MS of **15a**

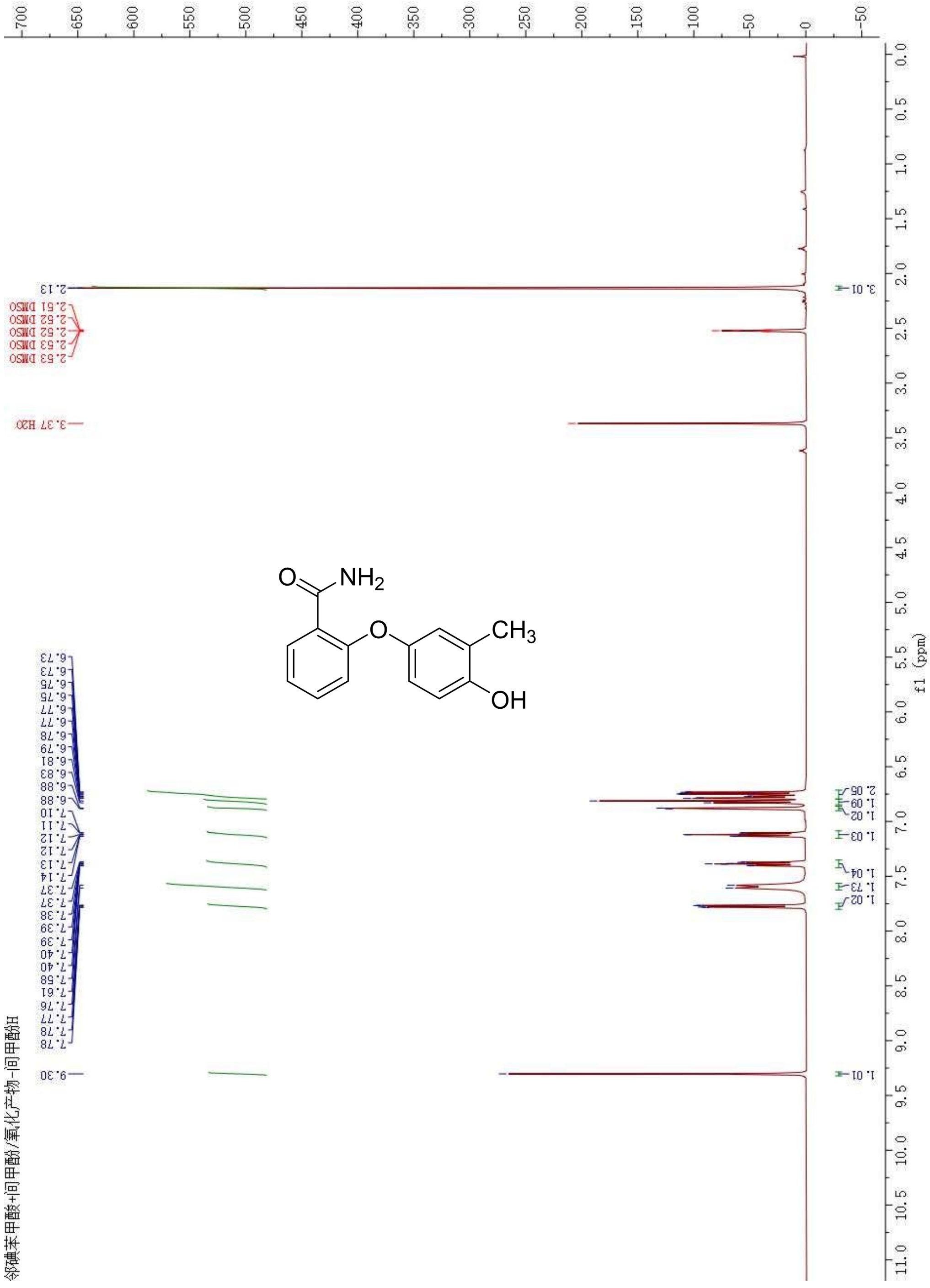

S40: ^{1}H-NMR of **15b**

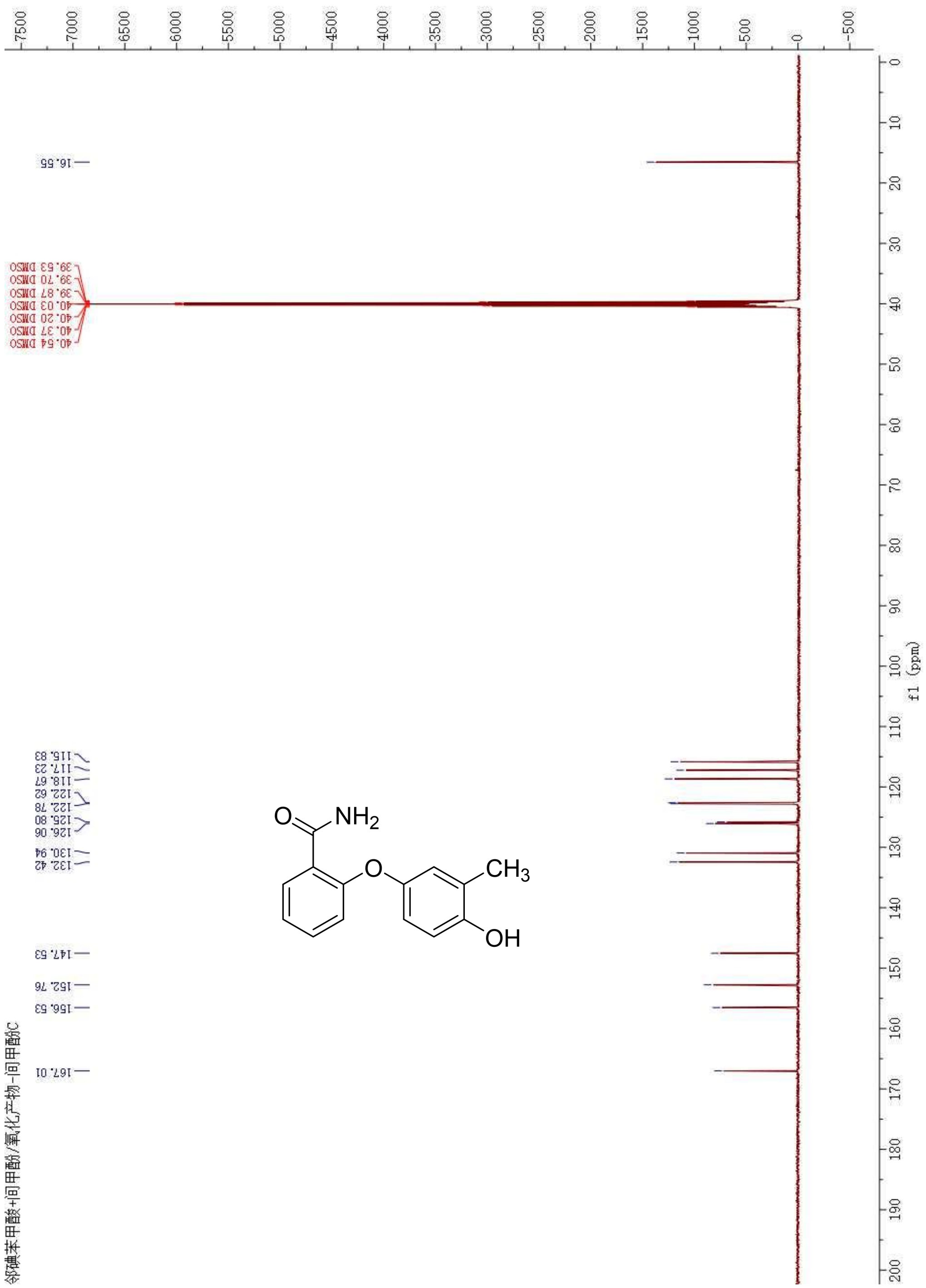

S41: 13C-NMR of **15b**

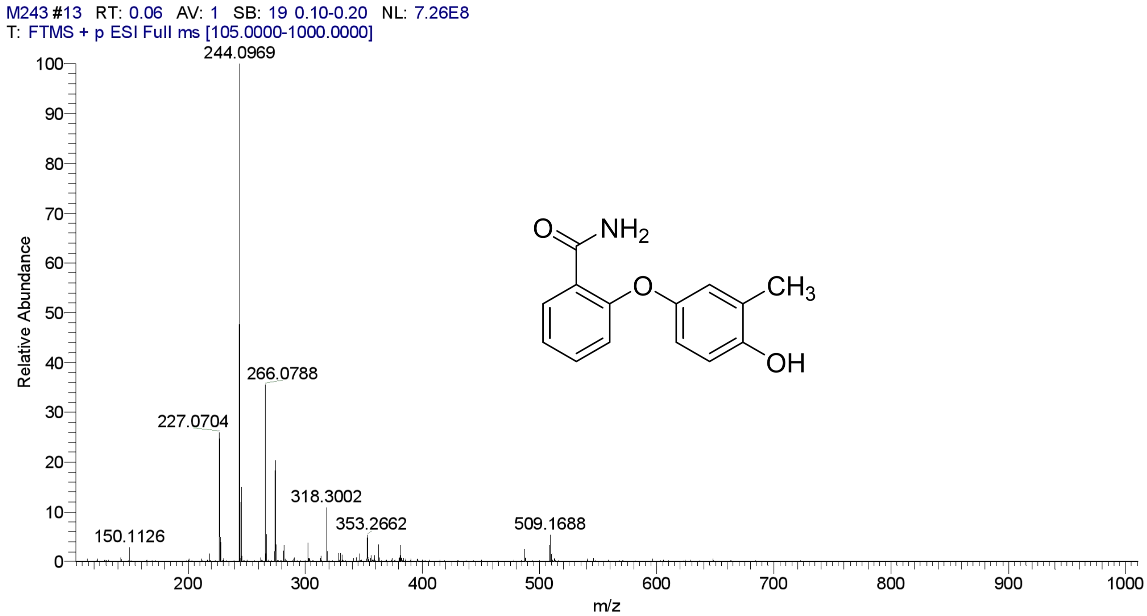

M243 #13 RT: 0.06 AV: 1 SB: 19 0.10-0.20 NL: 7.26E8
T: FTMS + p ESI Full ms [105.0000-1000.0000]
244.0969
266.0788
227.0704
318.3002
353.2662
509.1688
150.1126
Relative Abundance
m/z
O
NH2
O
CH3
OH

S42:MS of **15b**

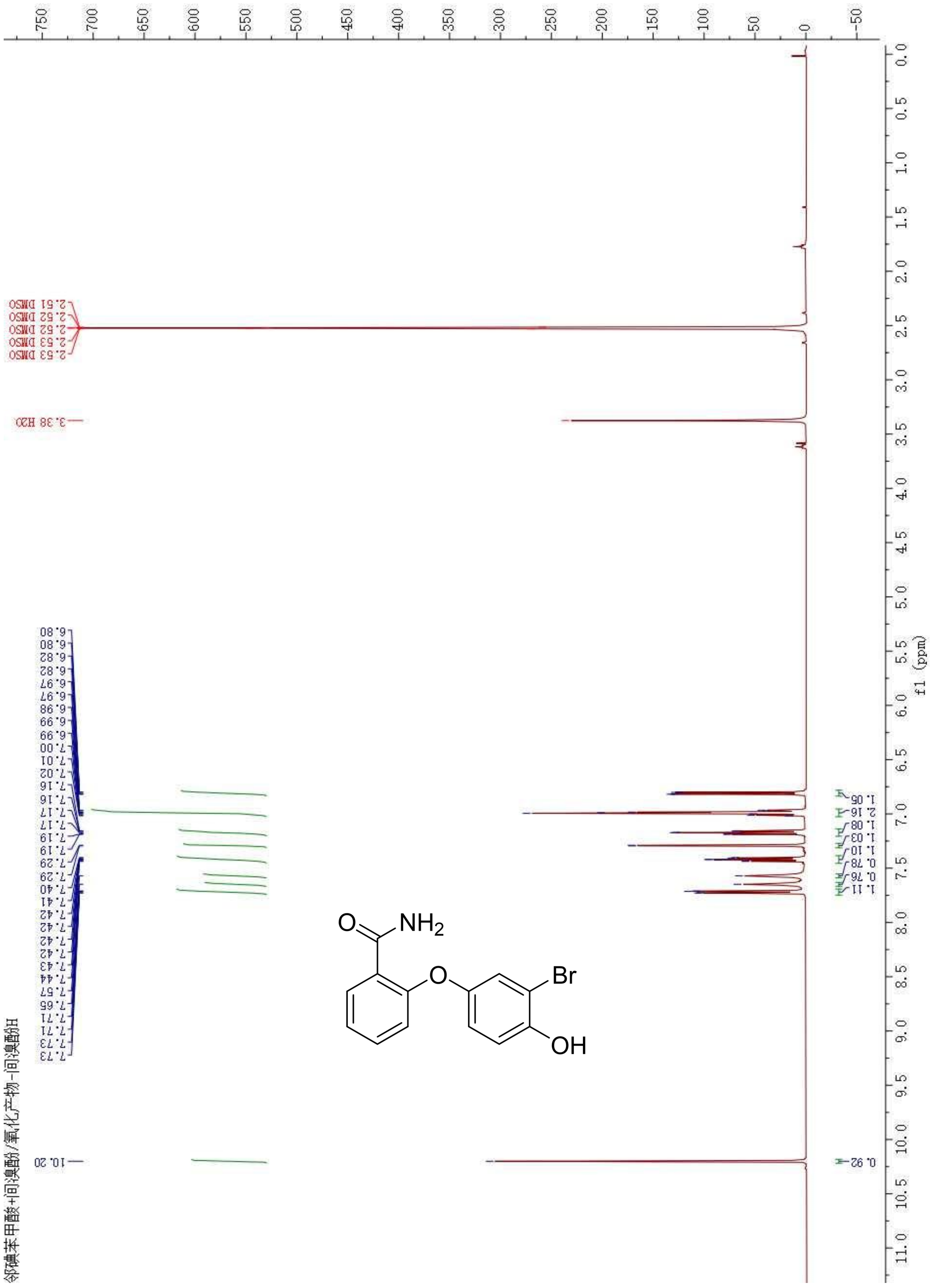

S43: ^{1}H-NMR of **15c**

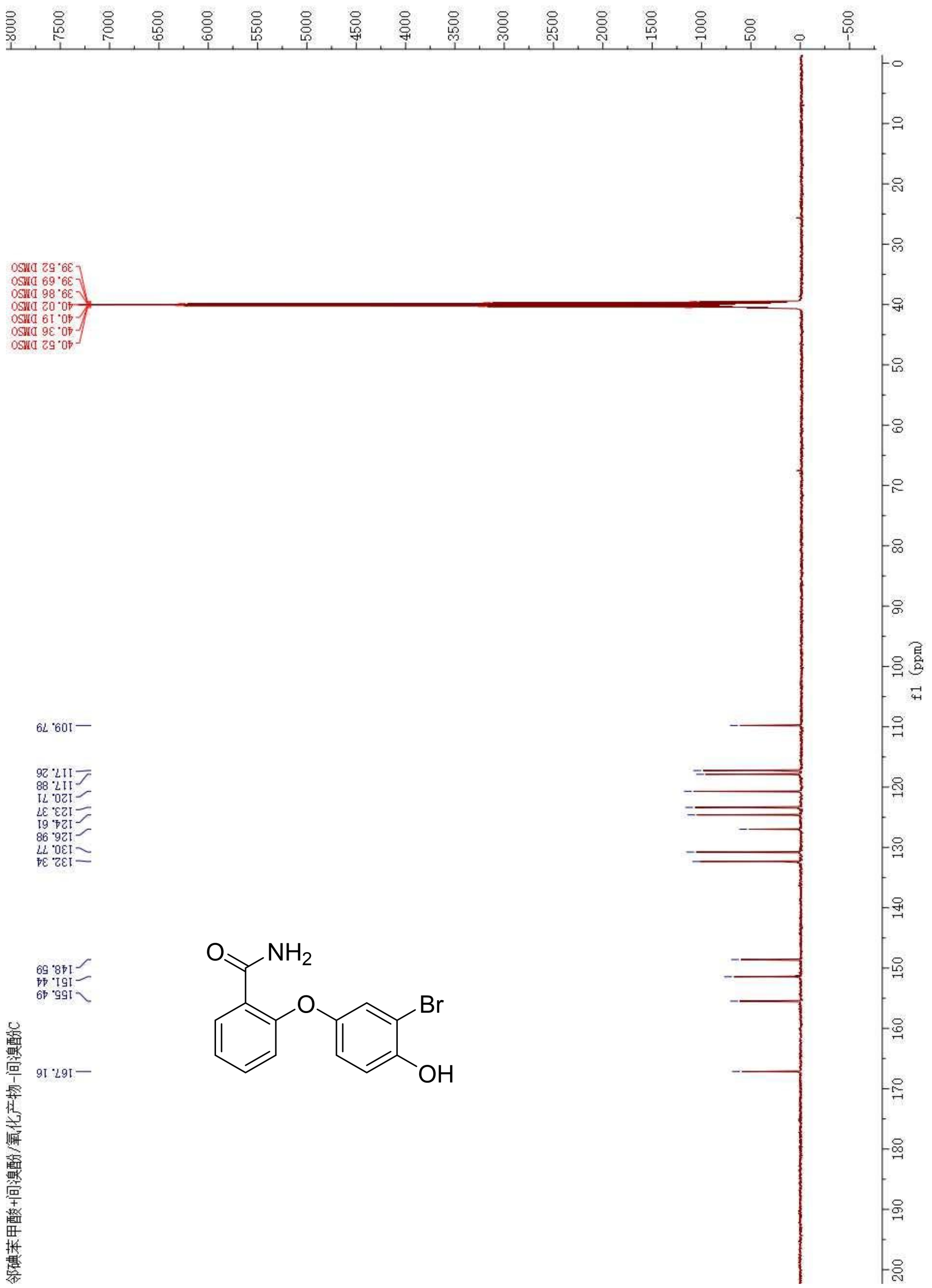

S44: ^{13}C-NMR of **15c**

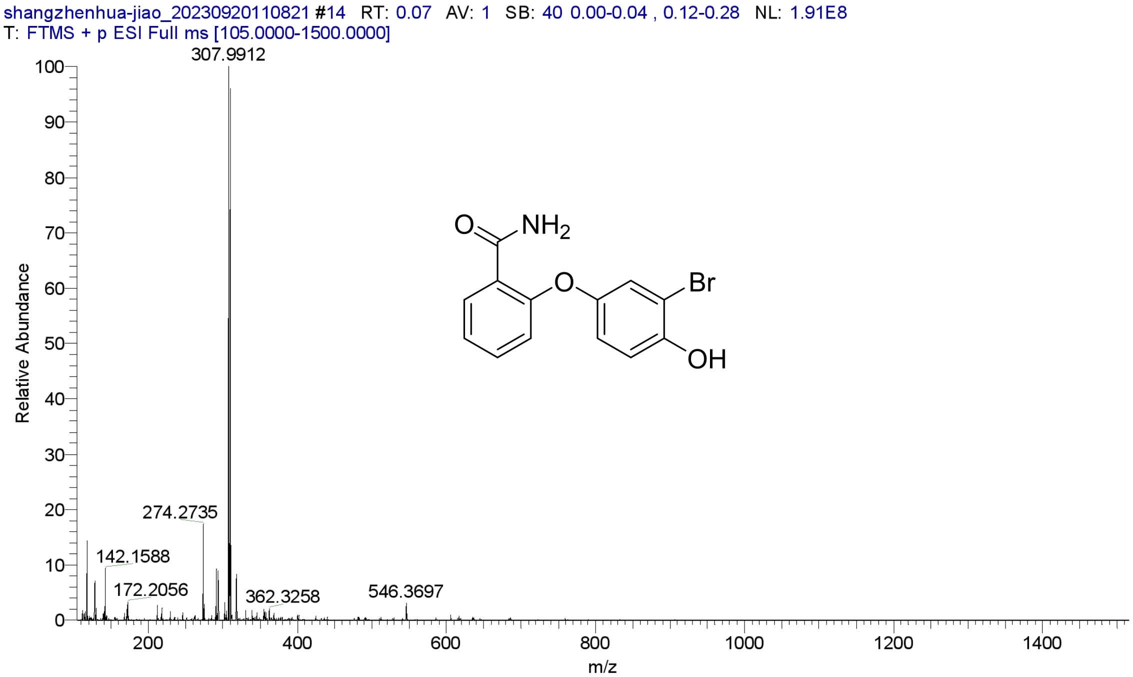
shangzhenhua-jiao_20230920110821 #14 RT: 0.07 AV: 1 SB: 40 0.00-0.04 , 0.12-0.28 NL: 1.91E8
T: FTMS + p ESI Full ms [105.0000-1500.0000]
307.9912
274.2735
142.1588
172.2056
362.3258
546.3697
Relative Abundance
m/z
NH2
O
O
Br
OH

S45:MS of **15c**

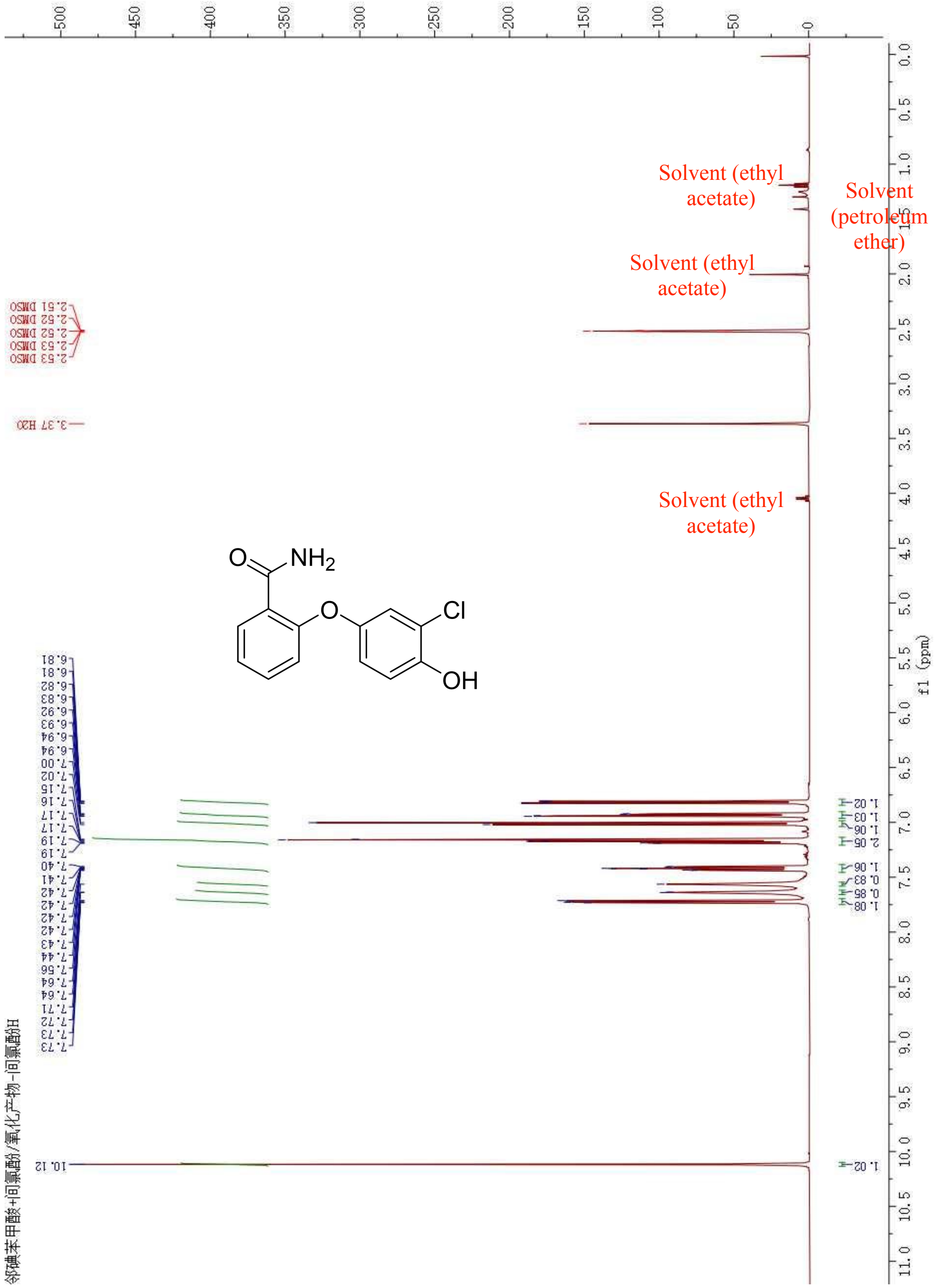

S46: ^{1}H-NMR of **15d**

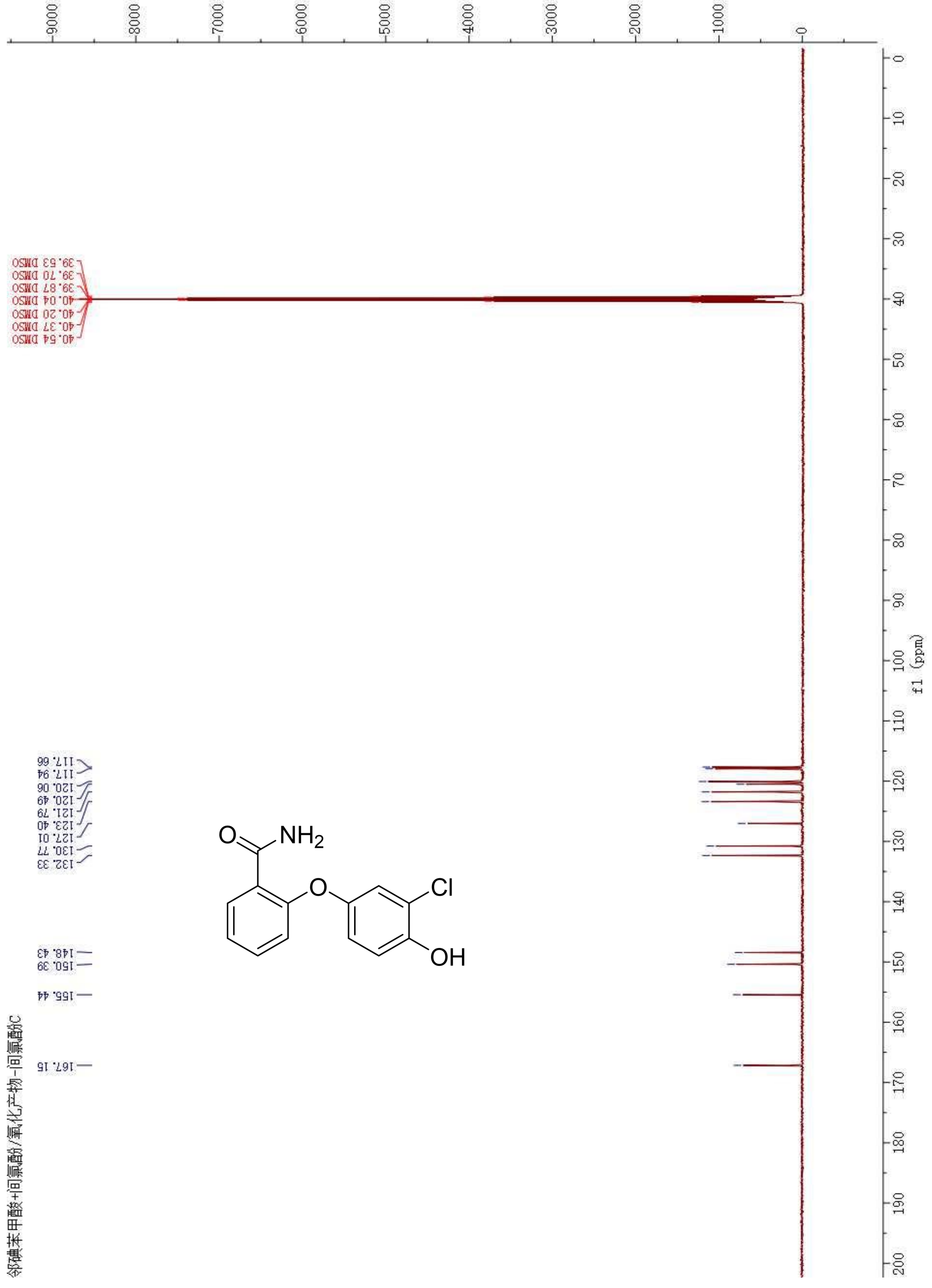

S47: ^{13}C-NMR of **15d**

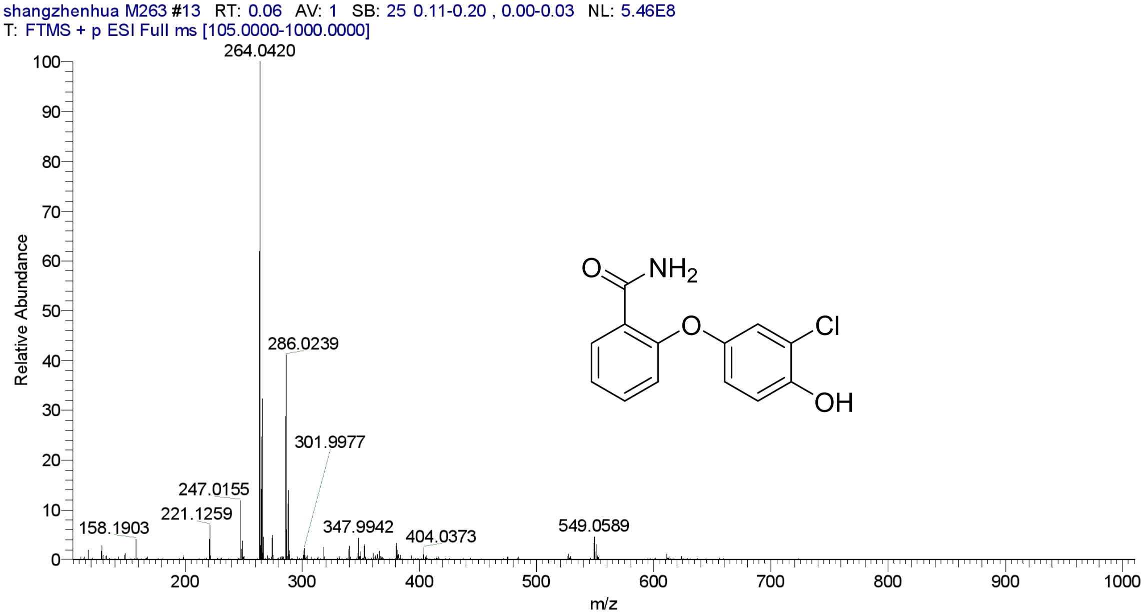
shangzhenhua M263 #13 RT: 0.06 AV: 1 SB: 25 0.11-0.20 , 0.00-0.03 NL: 5.46E8
T: FTMS + p ESI Full ms [105.0000-1000.0000]
264.0420
286.0239
301.9977
247.0155
221.1259
158.1903
347.9942
404.0373
549.0589
Relative Abundance
m/z
O
NH2
O
Cl
OH

S48:MS of **15d**

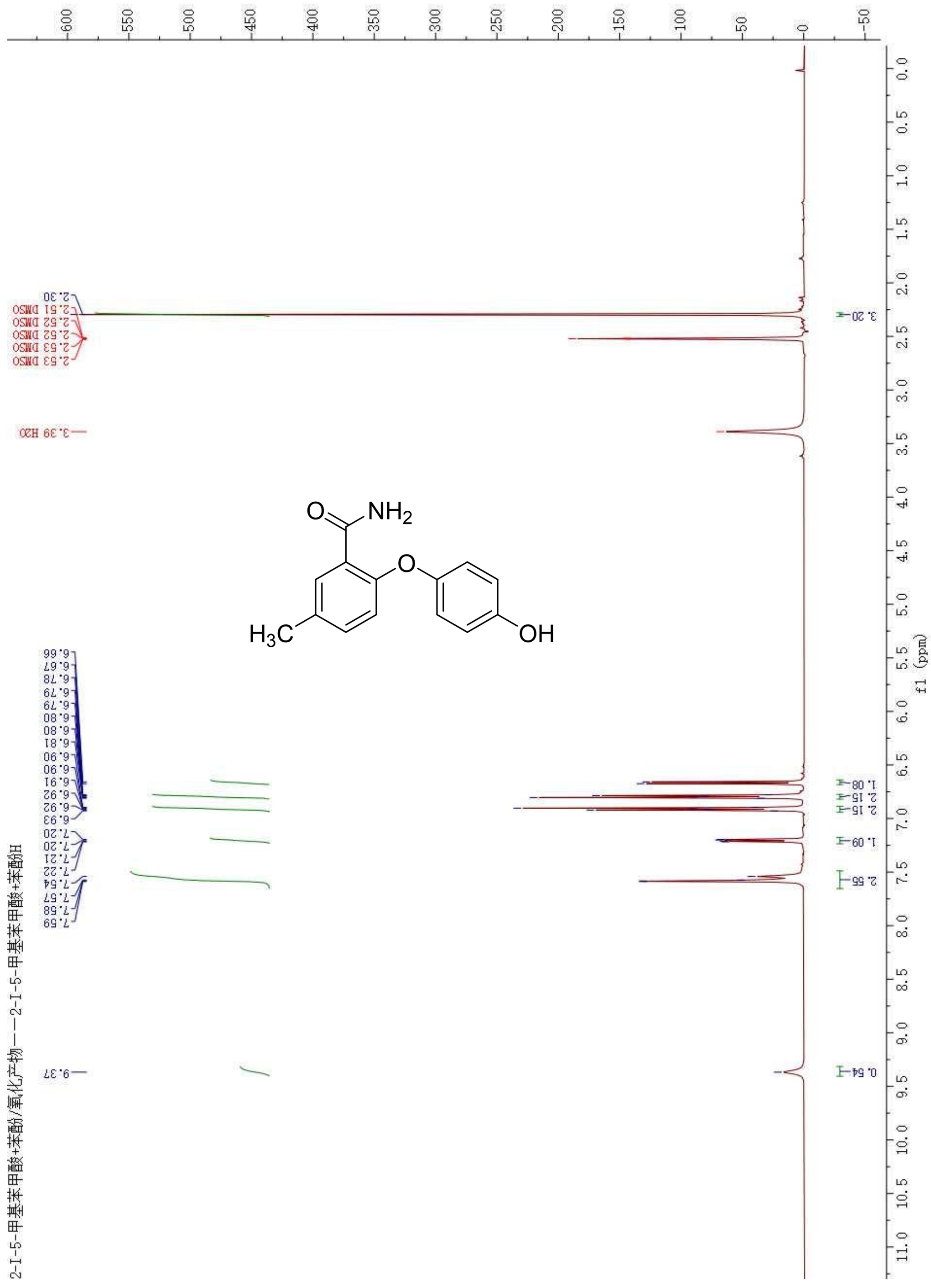

S49: ¹H NMR of **15e**

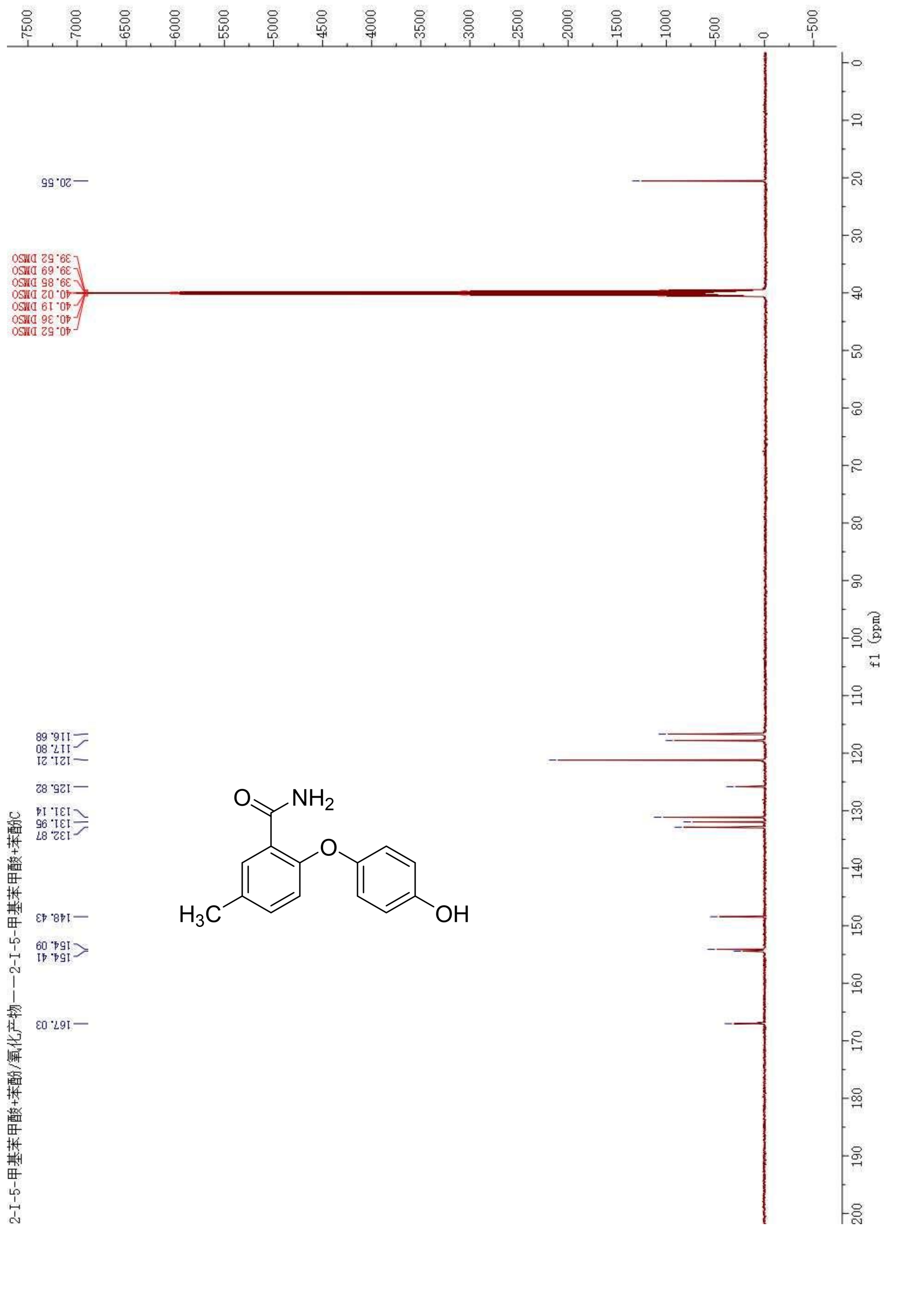

S$_{50}$: ^{13}C-NMR of **15e**

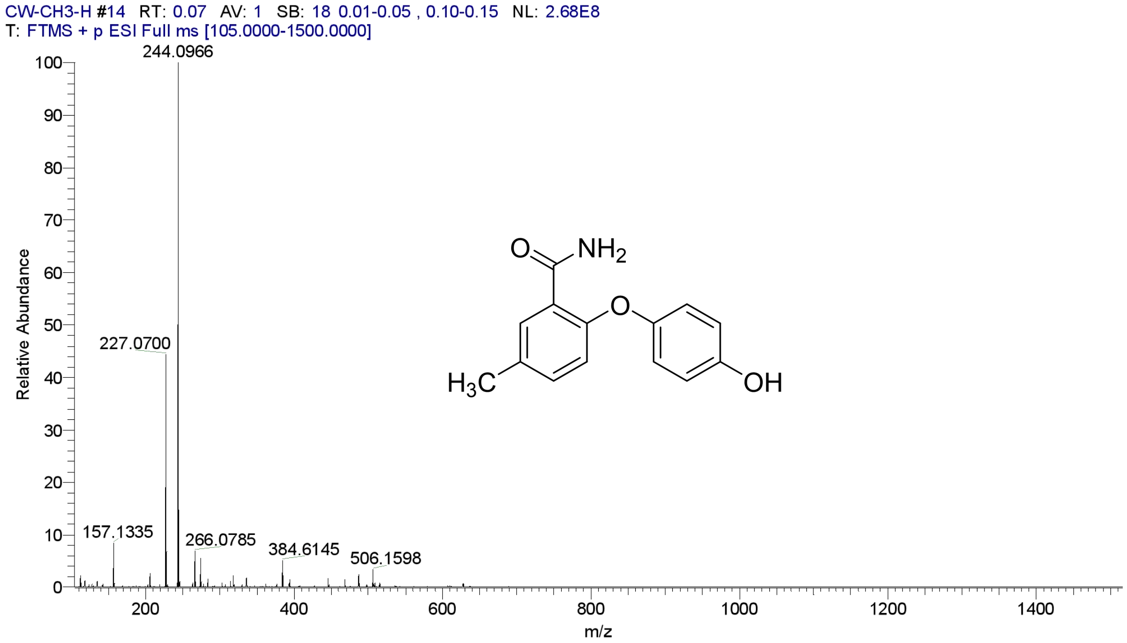

S$_{51}$: HRMS of **15e**

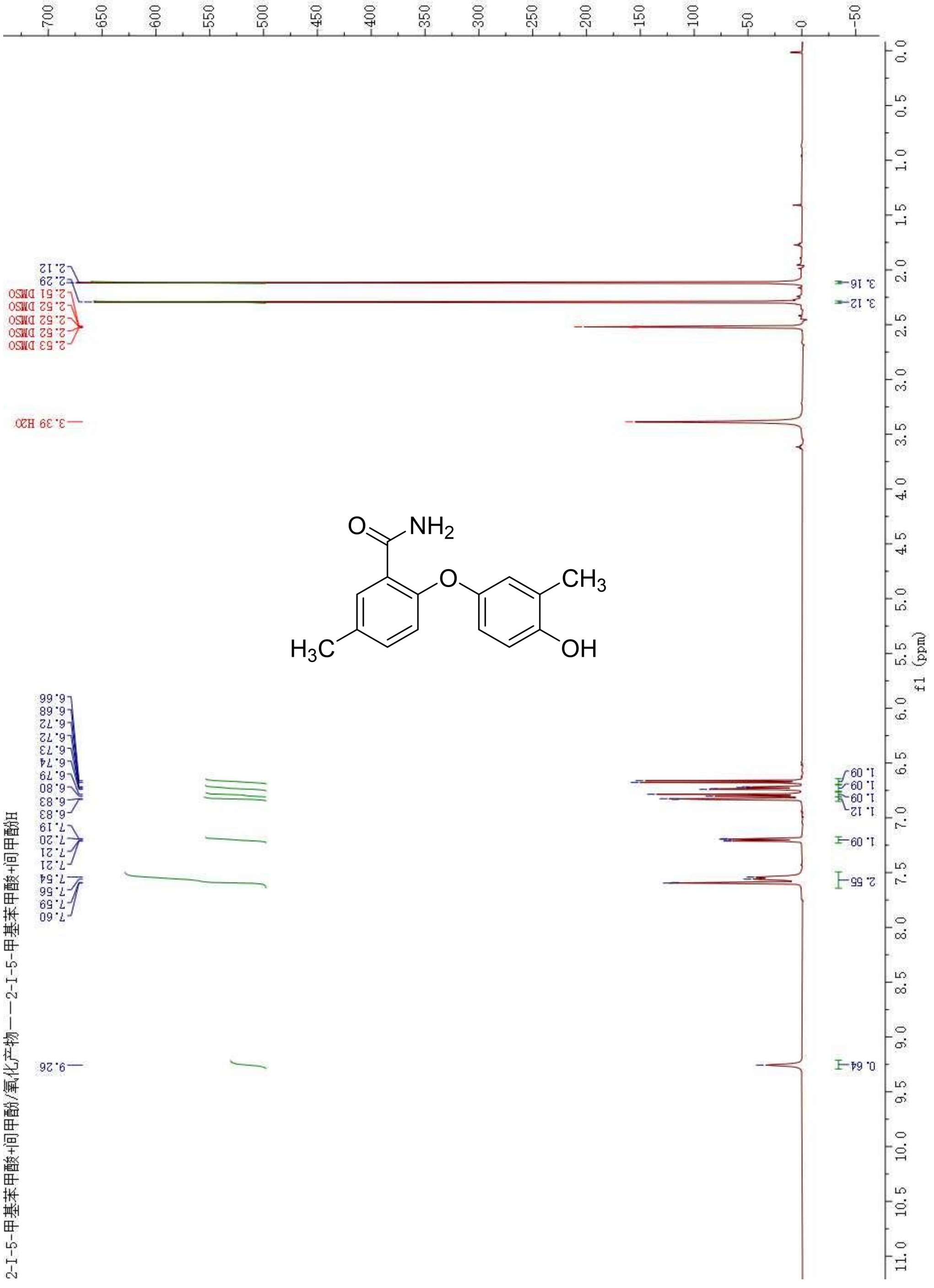

S_{52}: ^{1}H NMR of **15f**

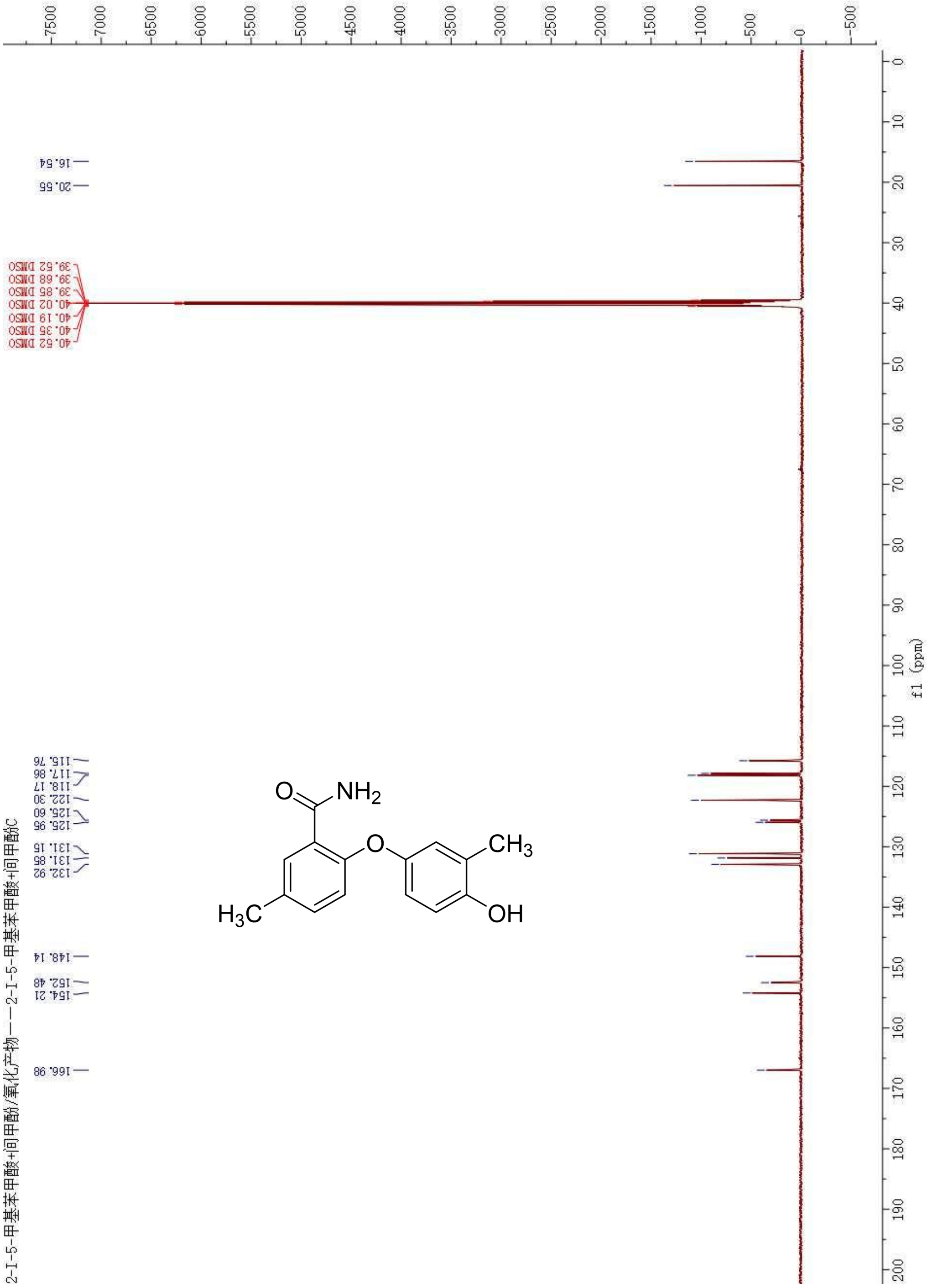

S53: ^{13}C-NMR of **15f**

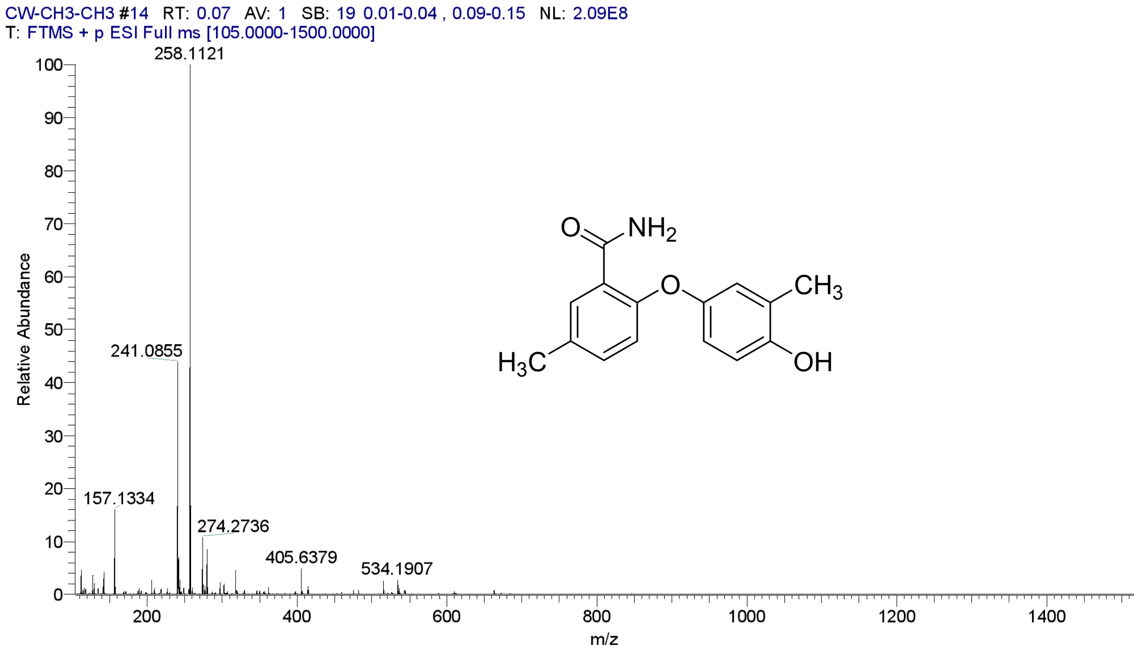

S54: HRMS of **15f**

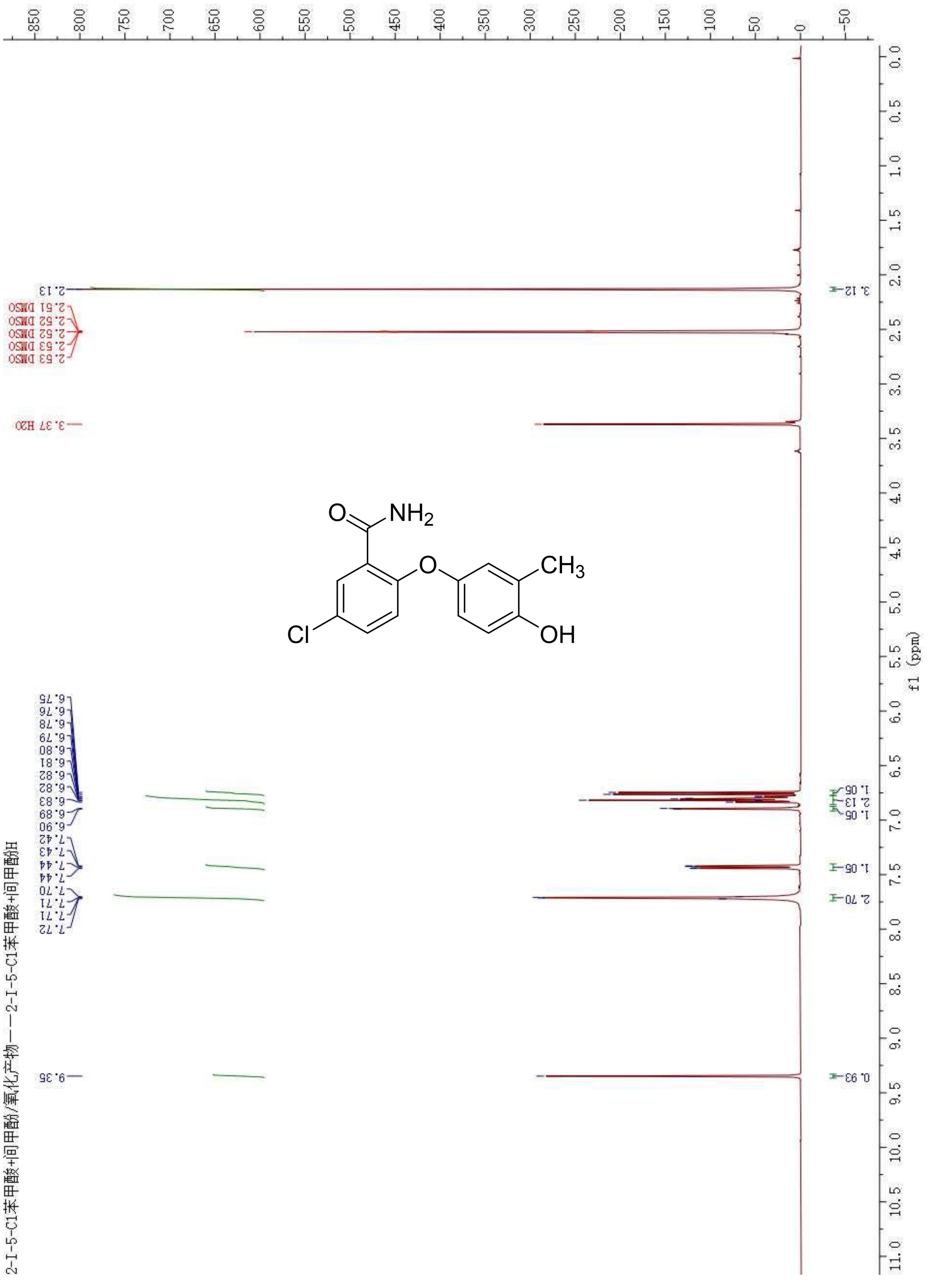

S55: ^{1}H NMR of **15g**

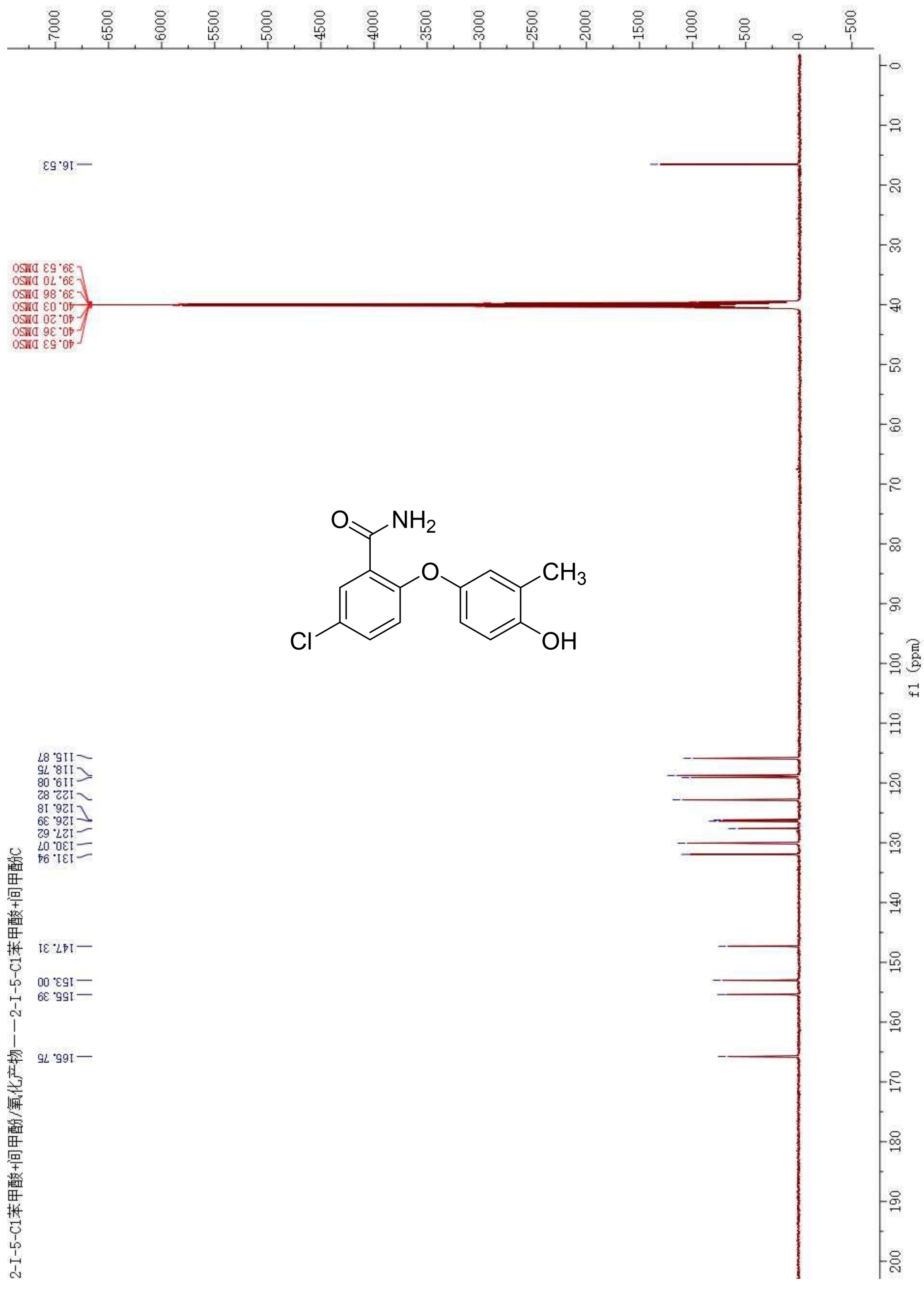

S56: ^{13}C-NMR of **15g**

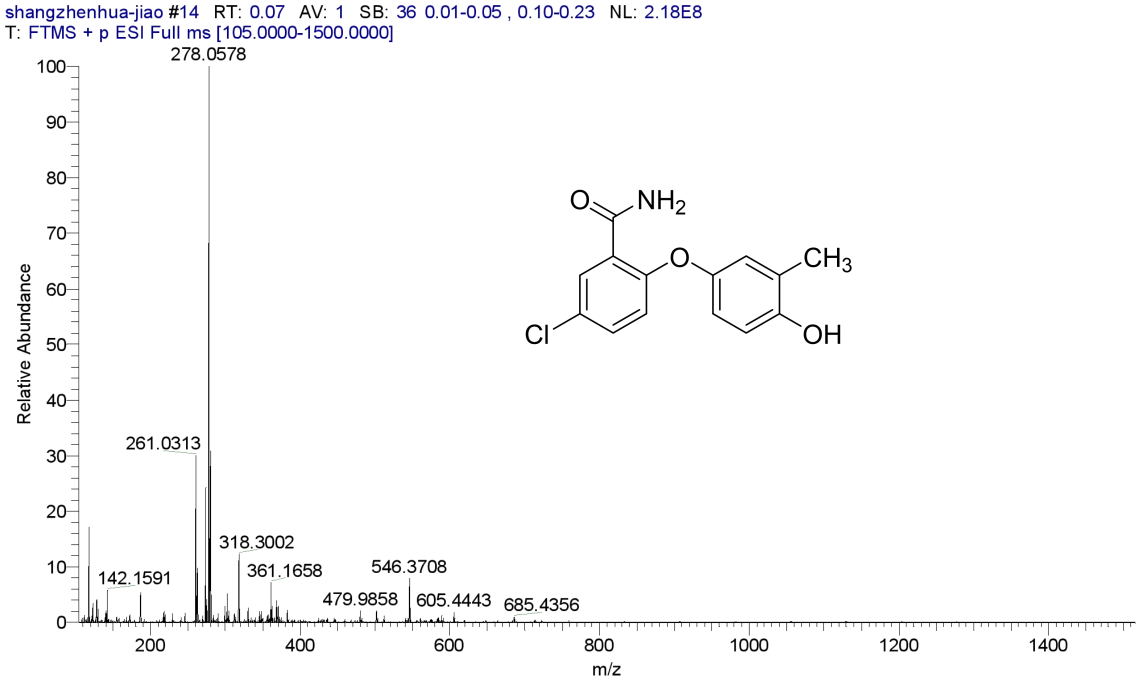

S57: HRMS of **15g**

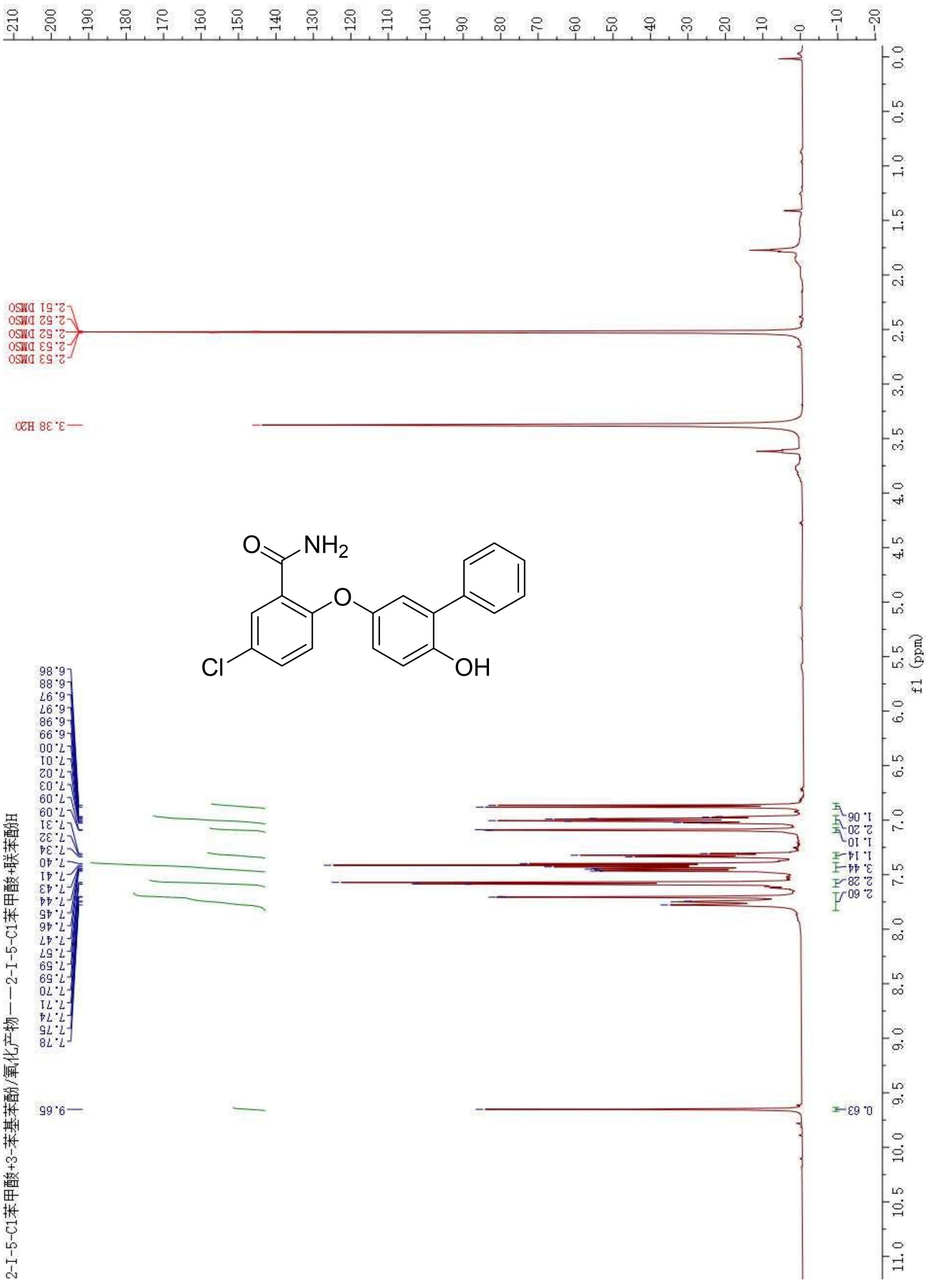

S58: ^{1}H NMR of **15h**

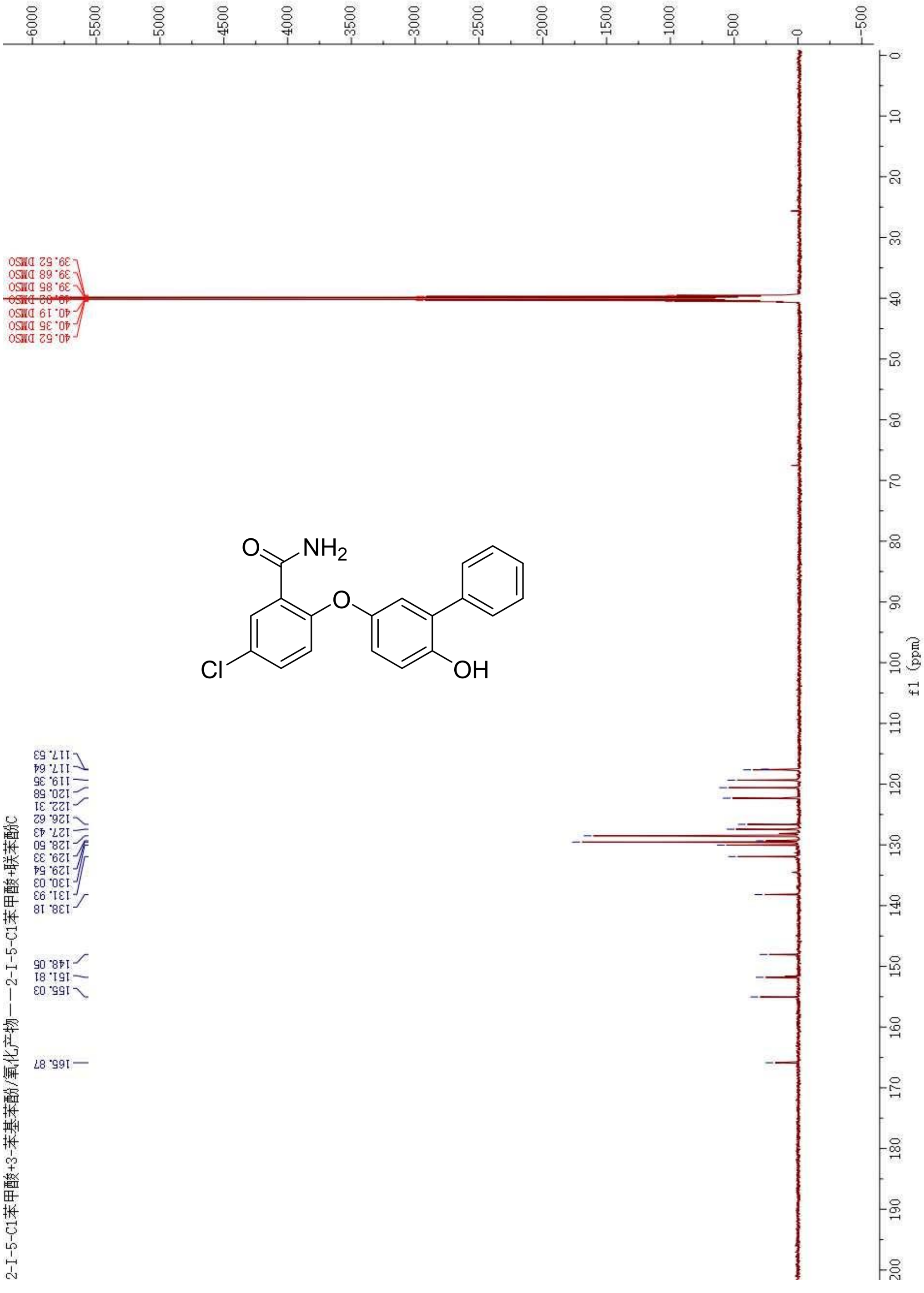

S59: ^{13}C-NMR of **15h**

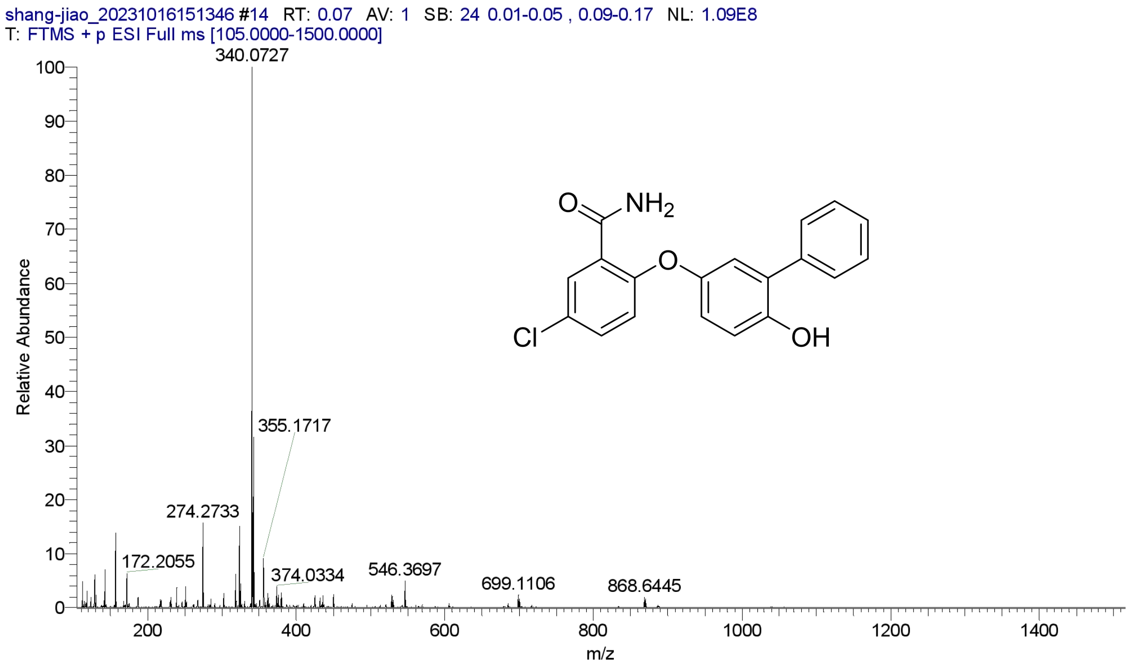

S60: HRMS of **15h**

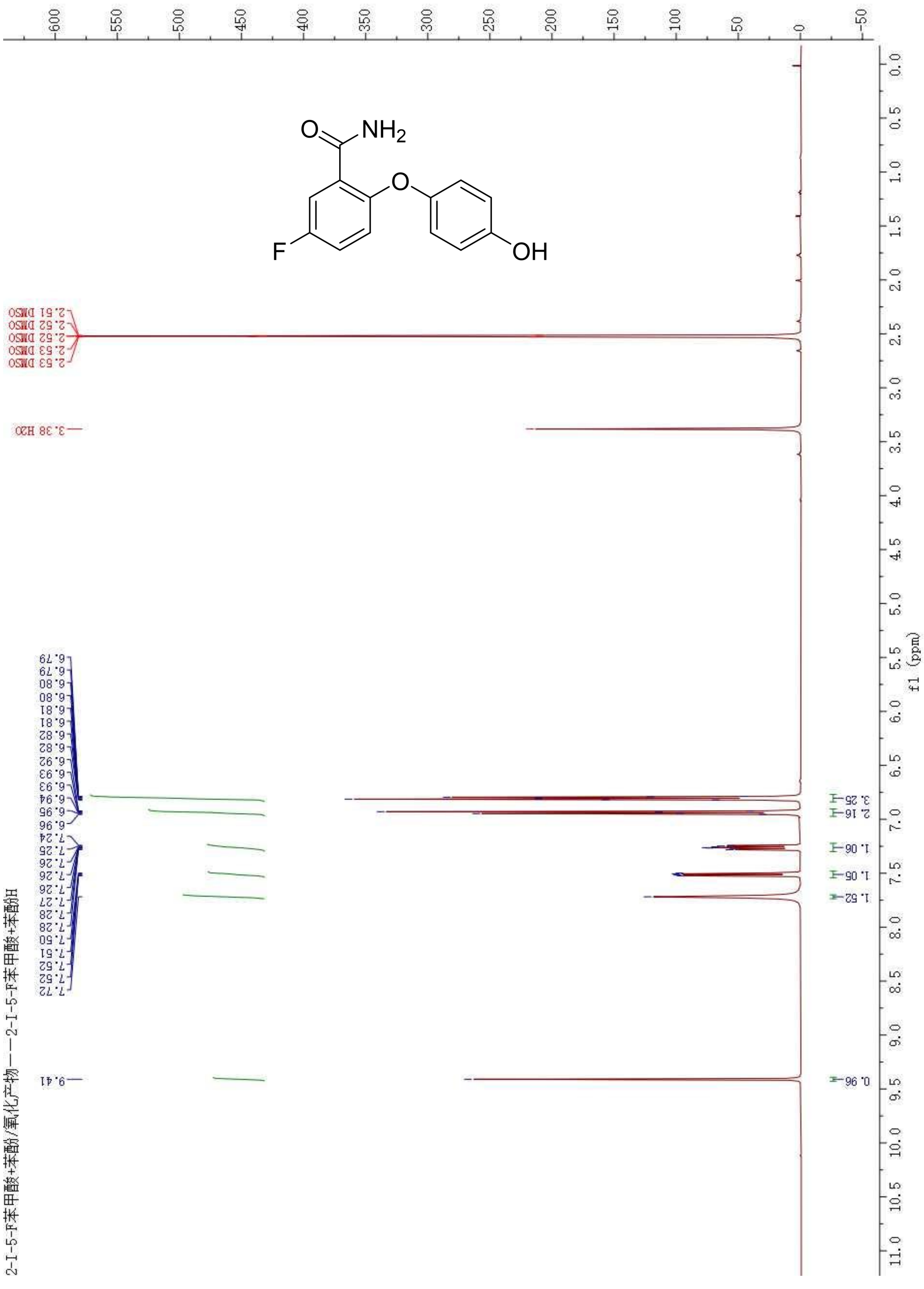

S$_{61}$: ^{1}H NMR of **15i**

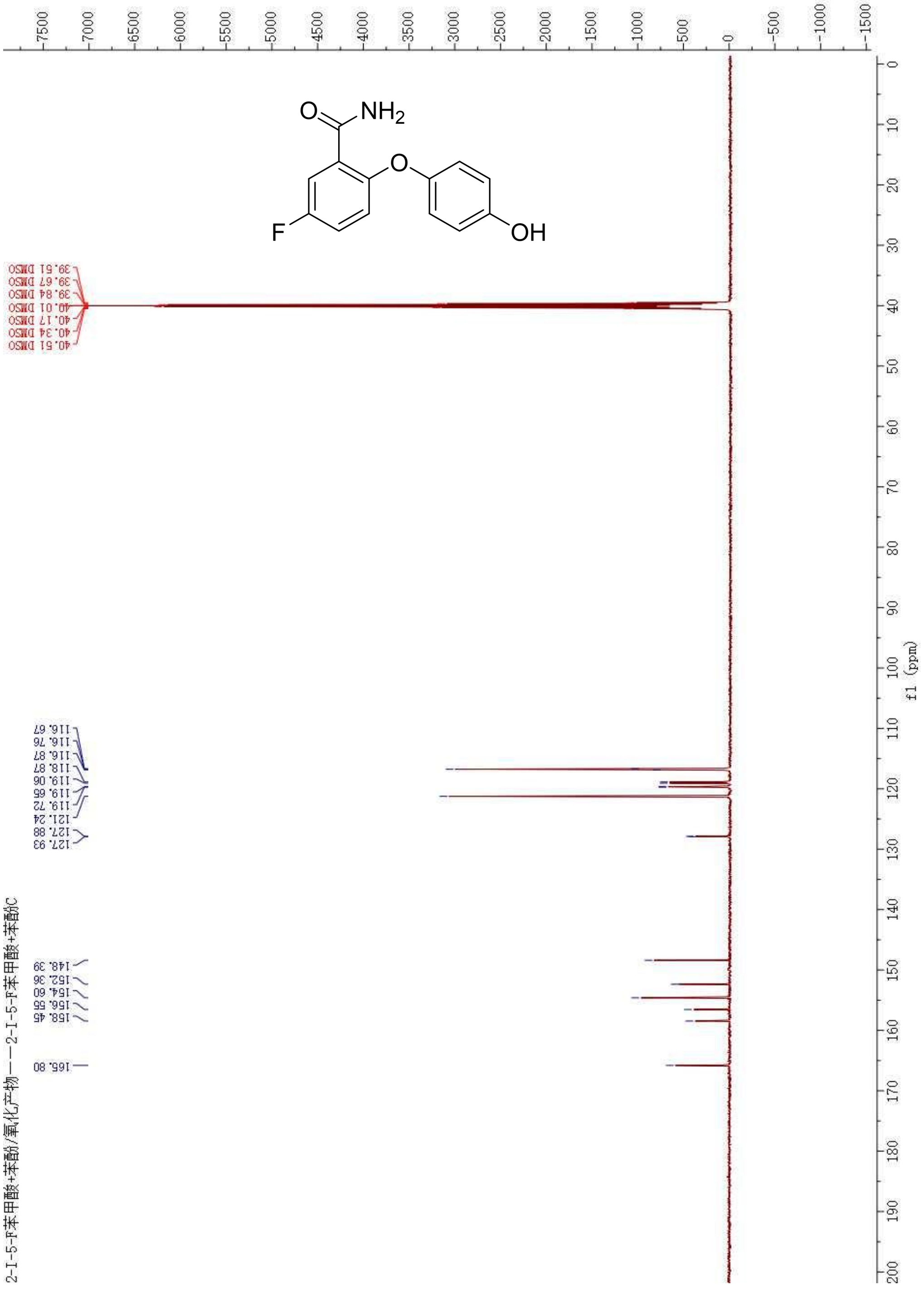

S$_{62}$: ^{13}C-NMR of **15i**

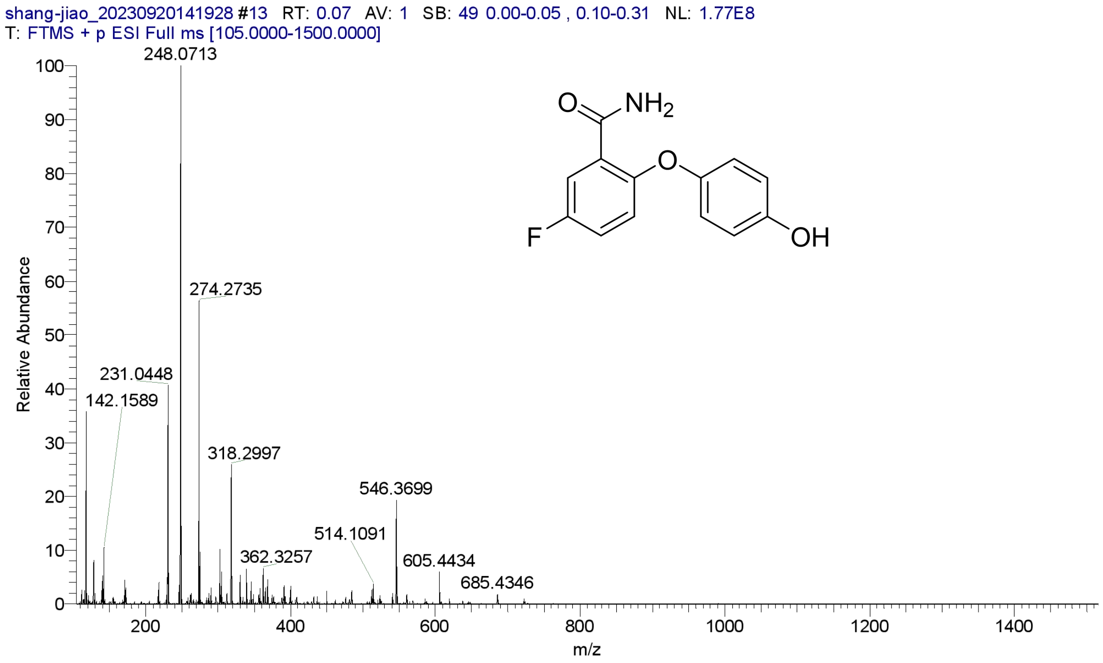

S63: HRMS of **15i**

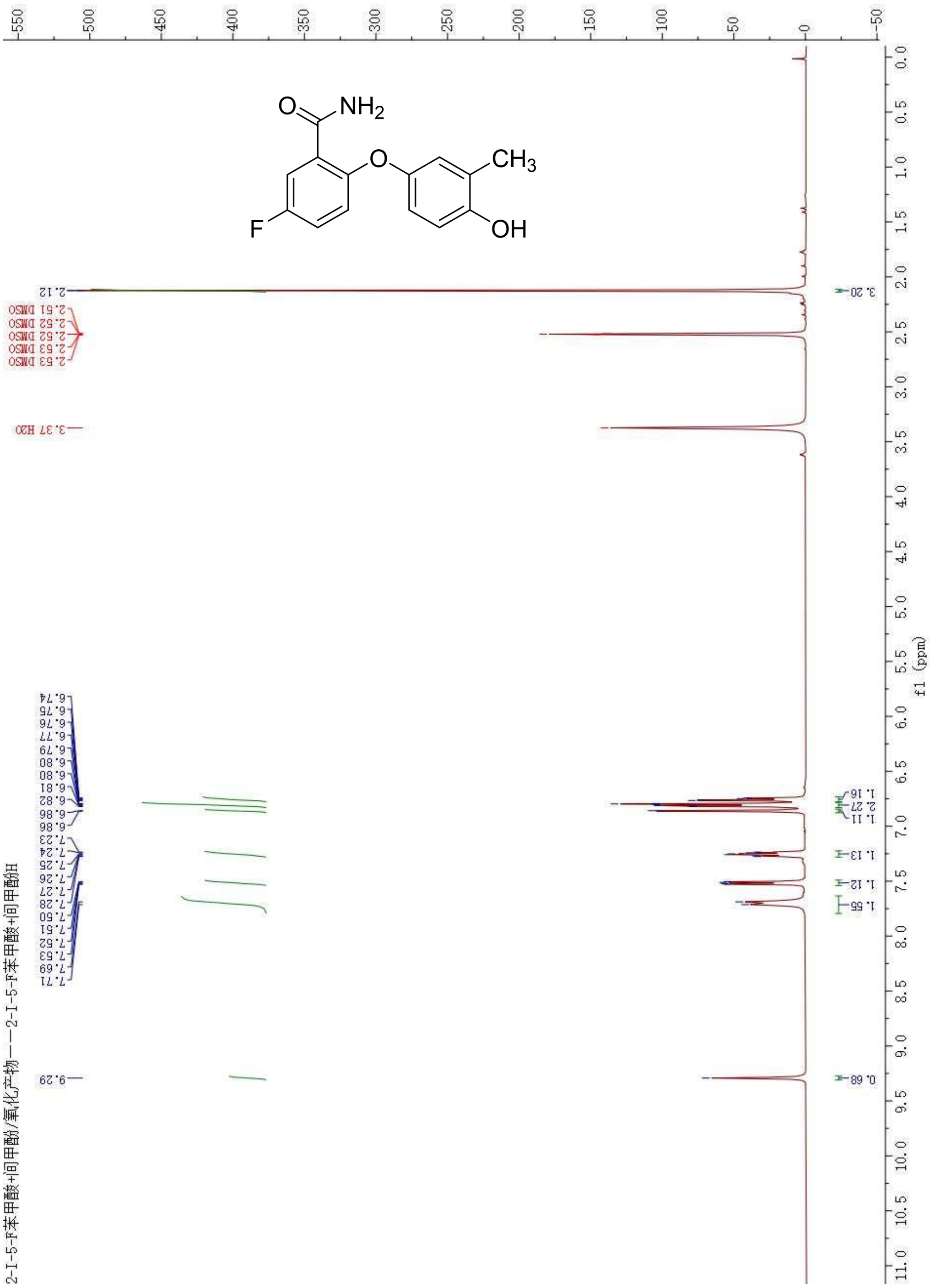

S64: ^{1}H NMR of **15j**

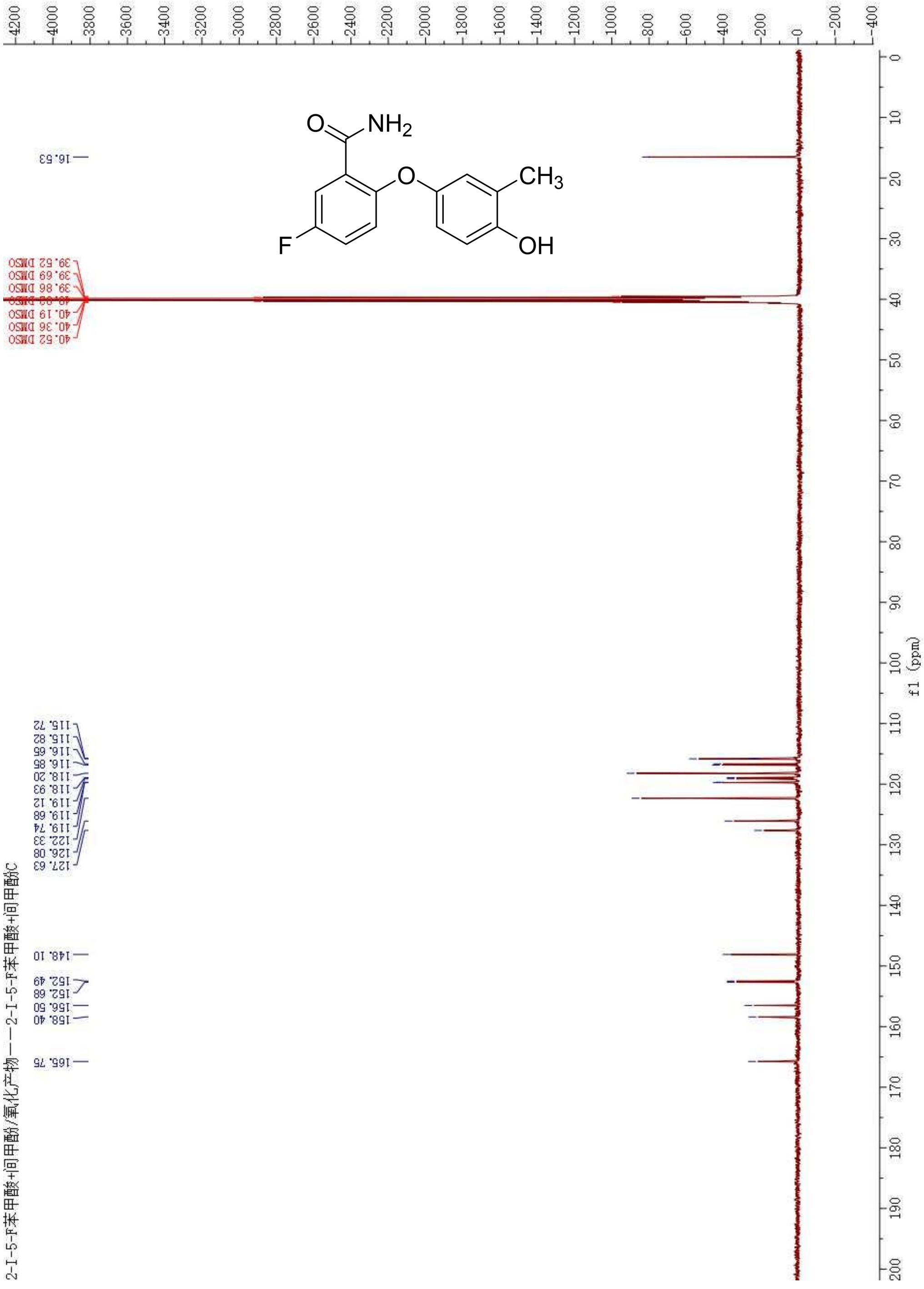

S$_{65}$: ^{13}C-NMR of **15j**

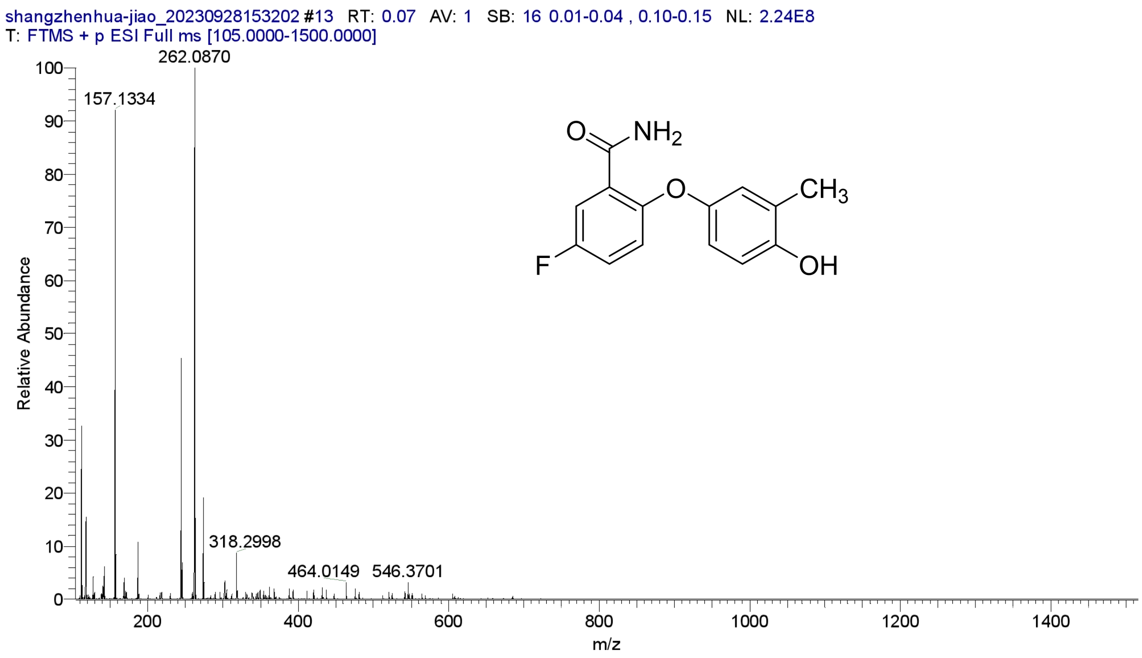

S$_{66}$: HRMS of **15j**

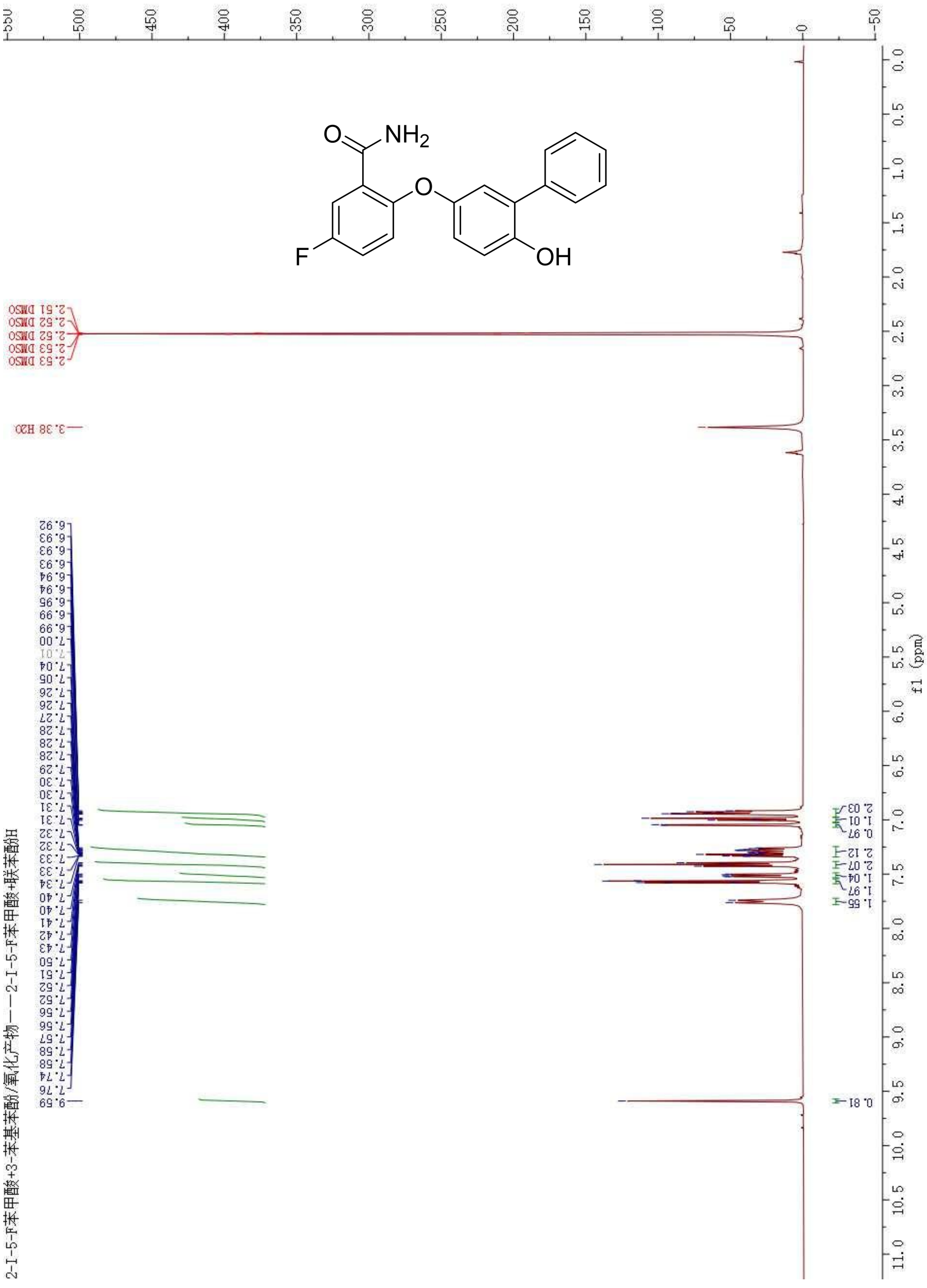

S$_{67}$: ^{1}H NMR of **15k**

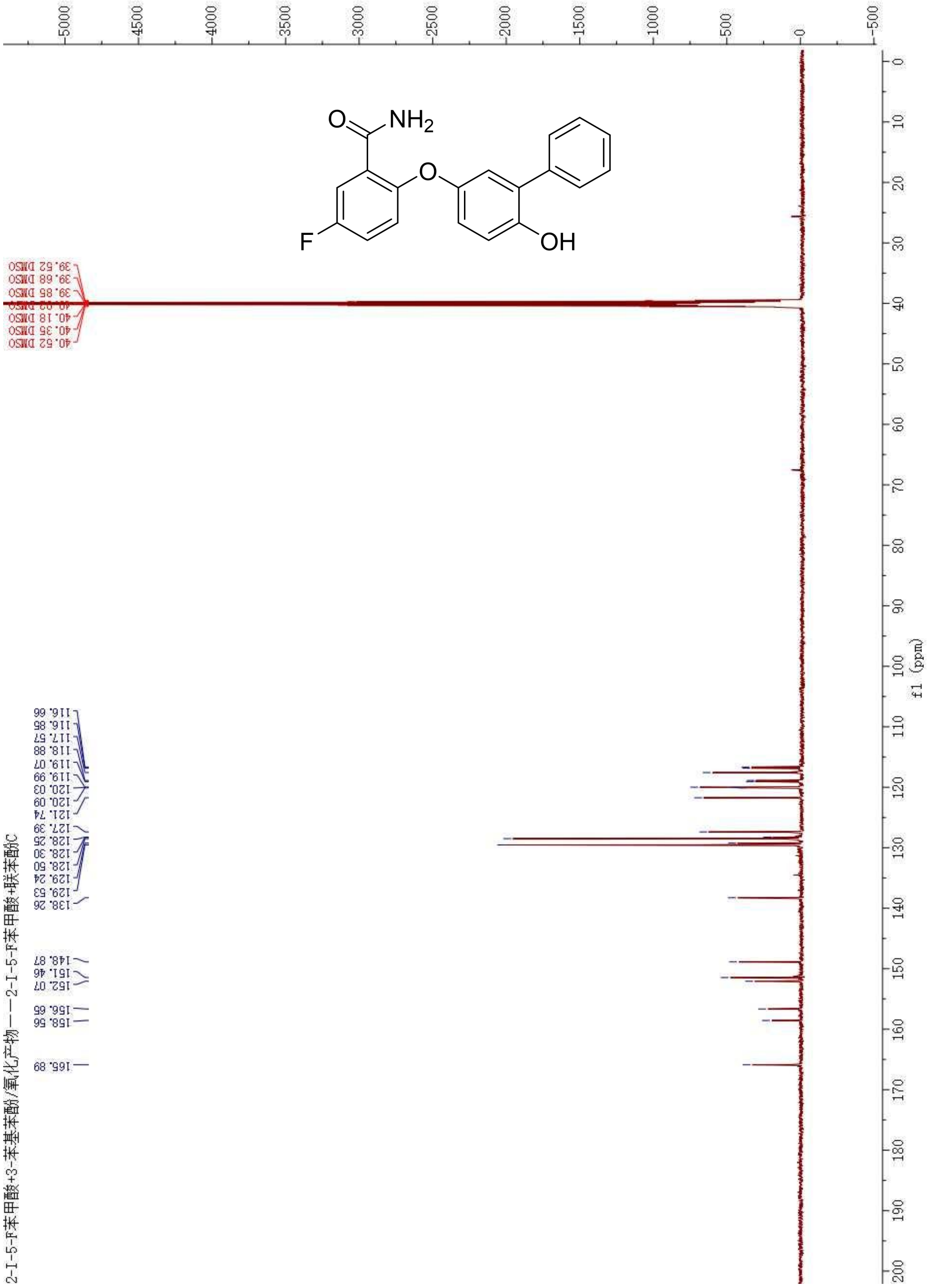

S$_{68}$: ^{13}C-NMR of **15k**

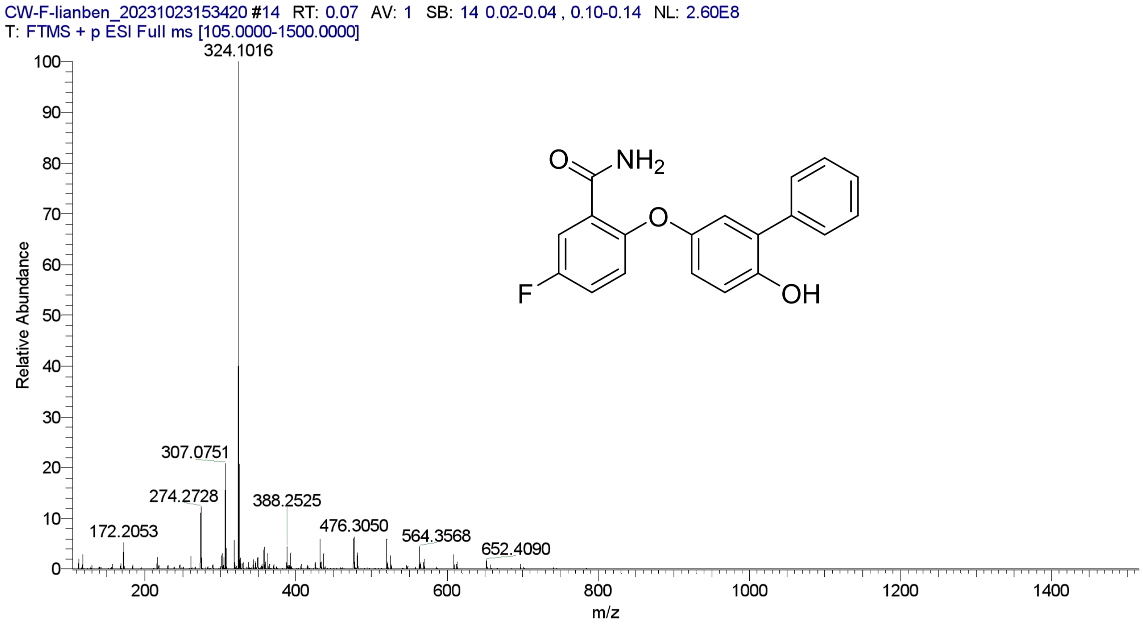

S69: HRMS of **15k**

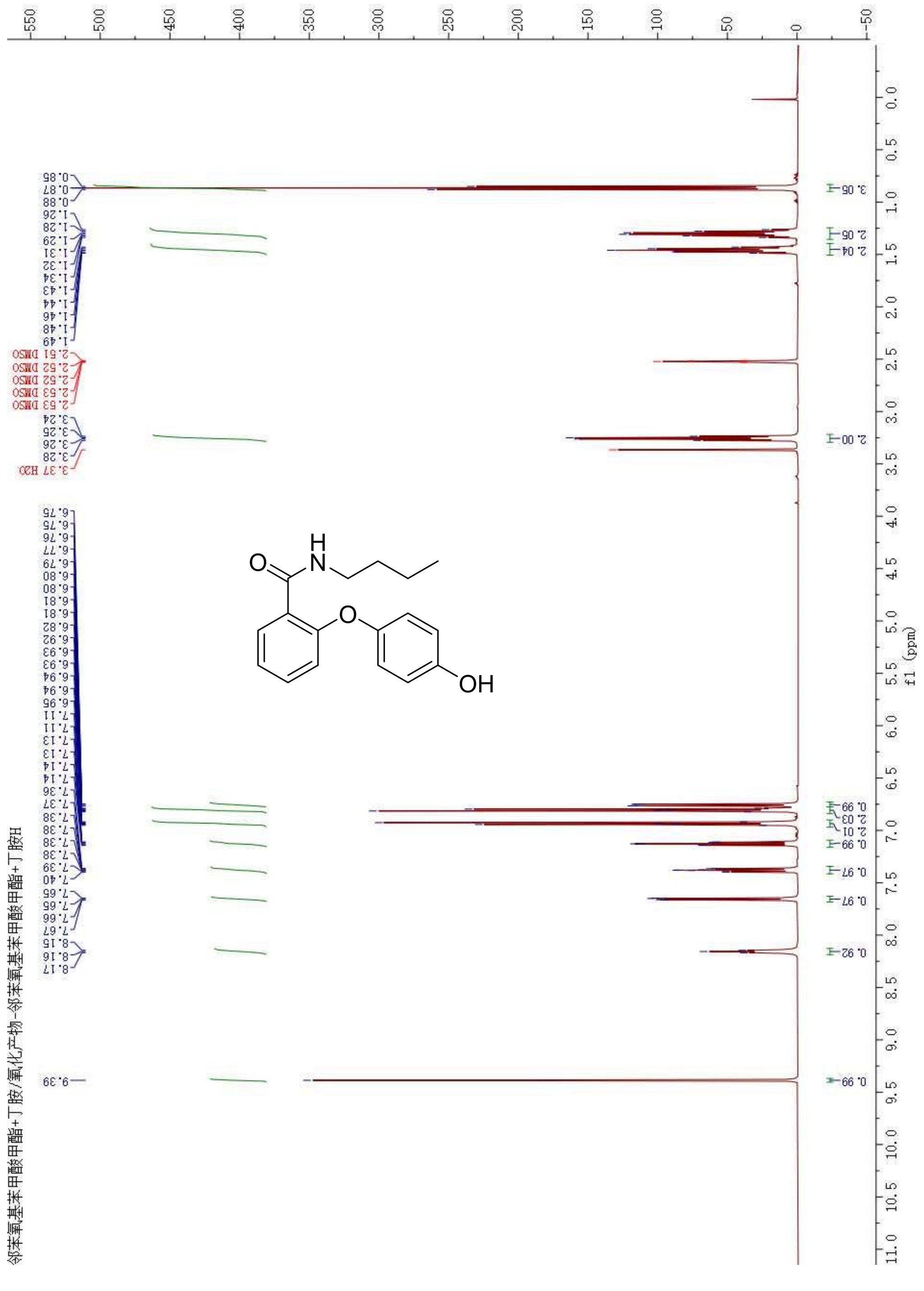

S$_{70}$: ^{1}H NMR of **15l**

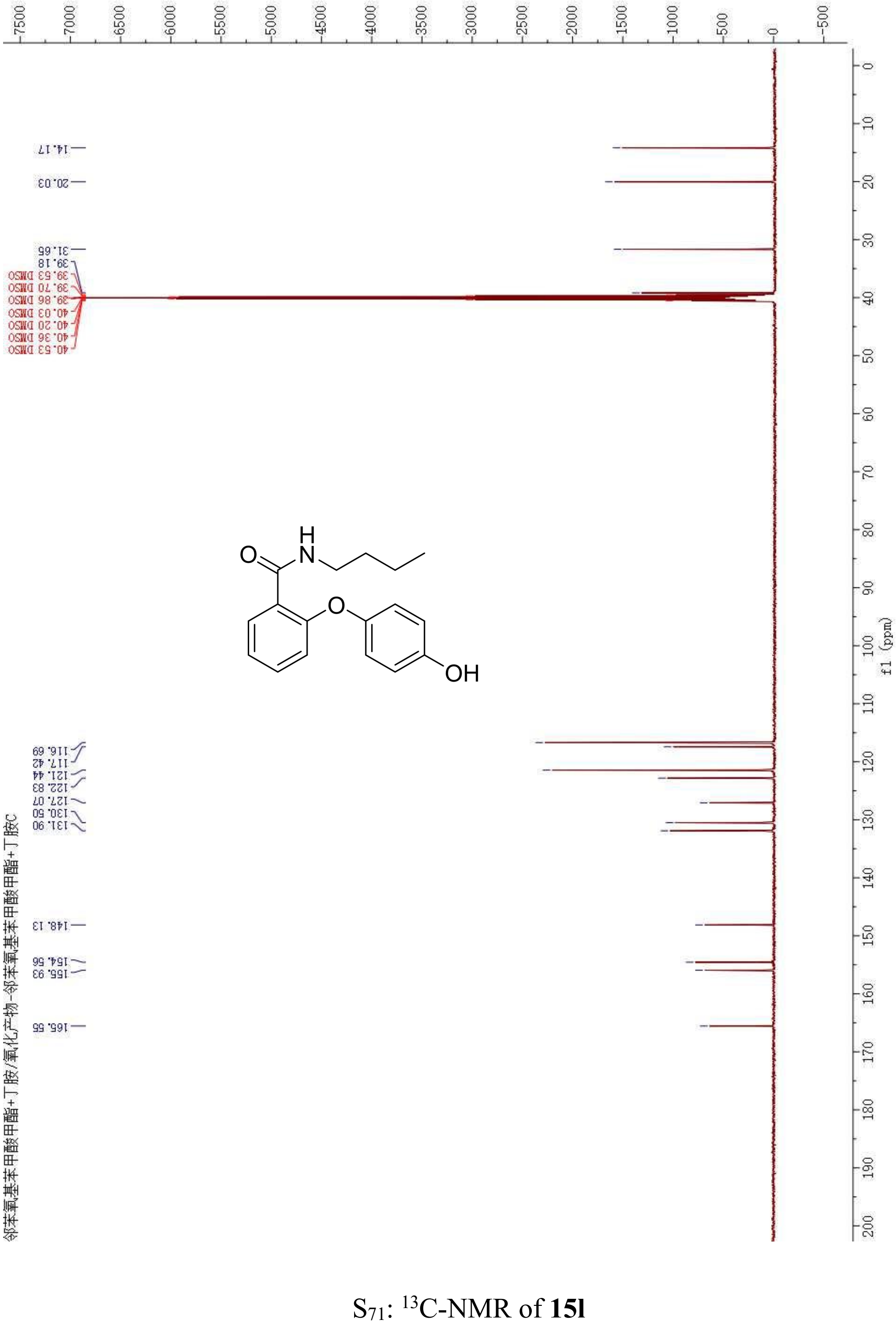

S$_{71}$: ^{13}C-NMR of **15l**

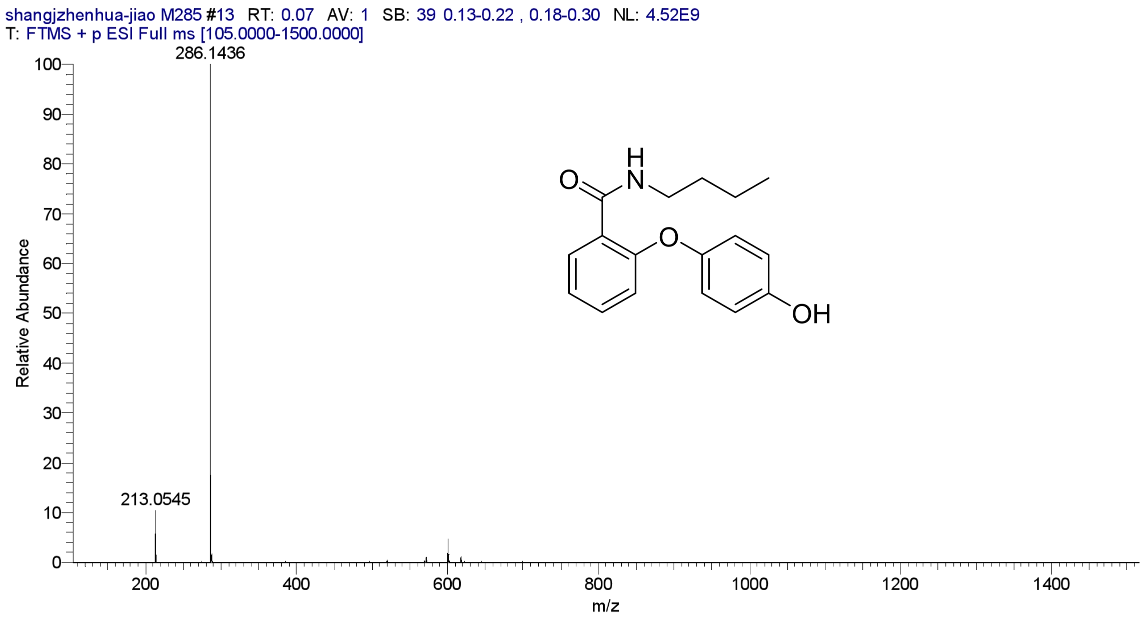

S72: HRMS of **15l**

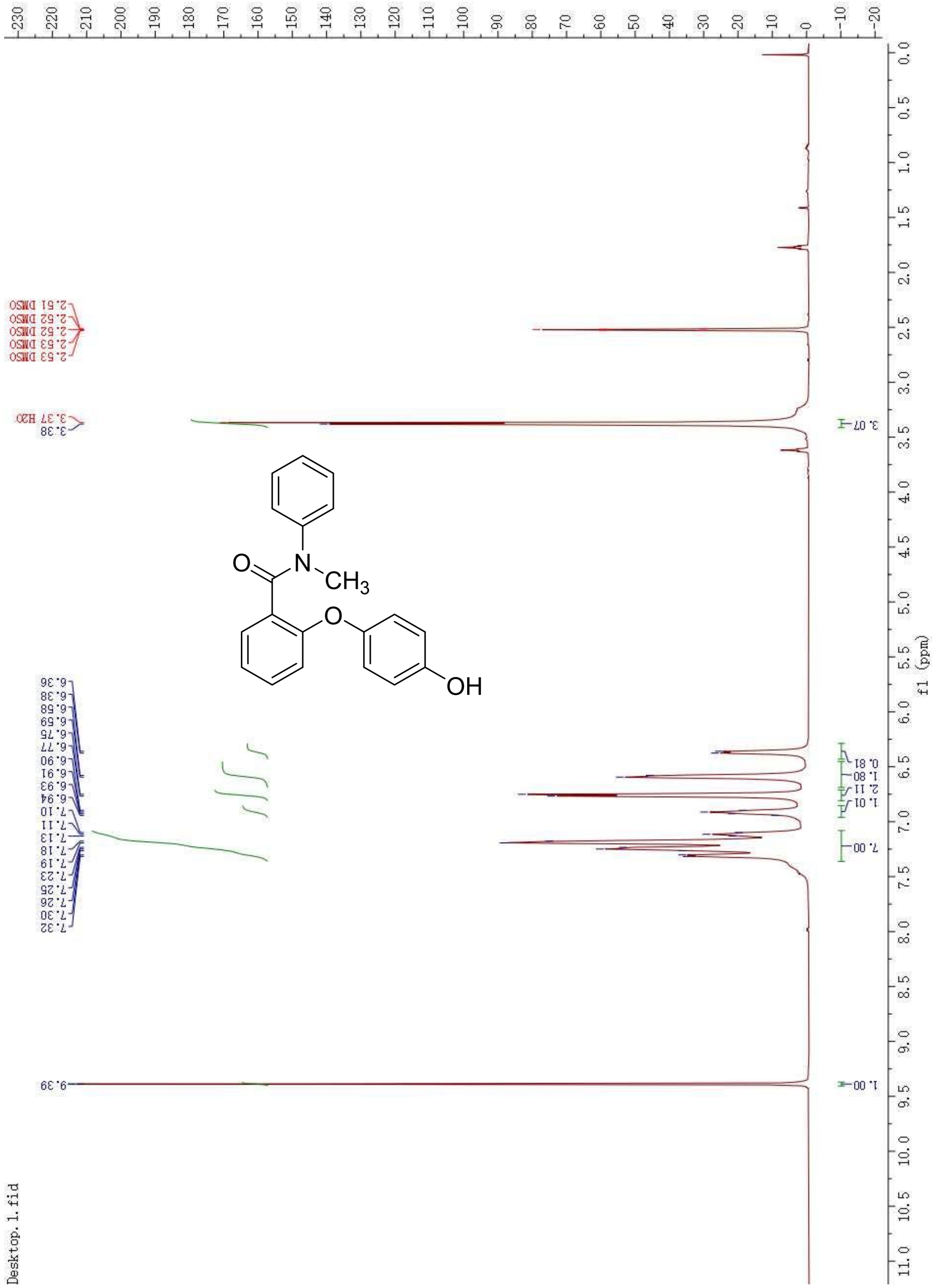

S$_{73}$: ^{1}H NMR of **15m**

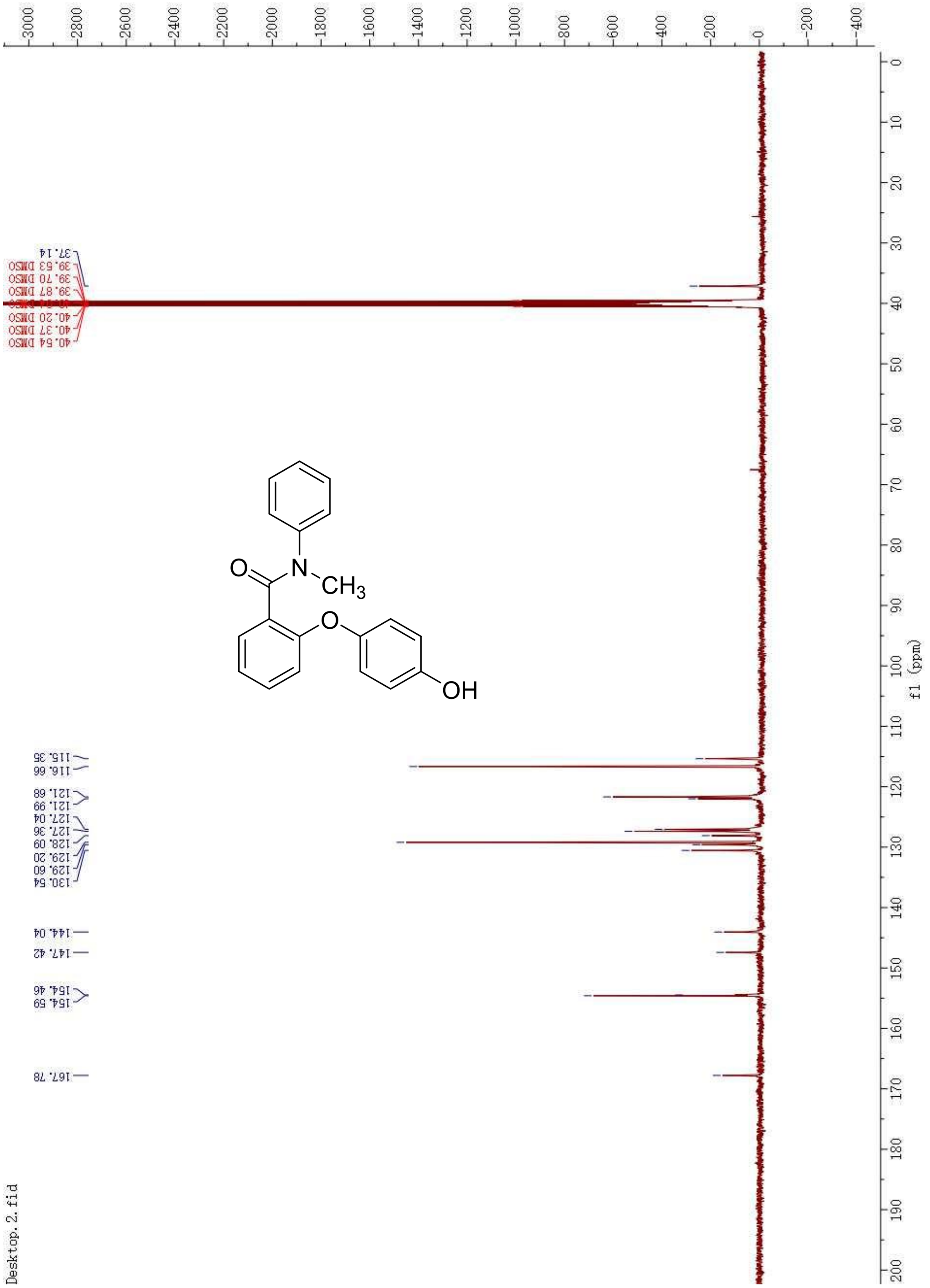

S$_{74}$: ^{13}C-NMR of **15m**

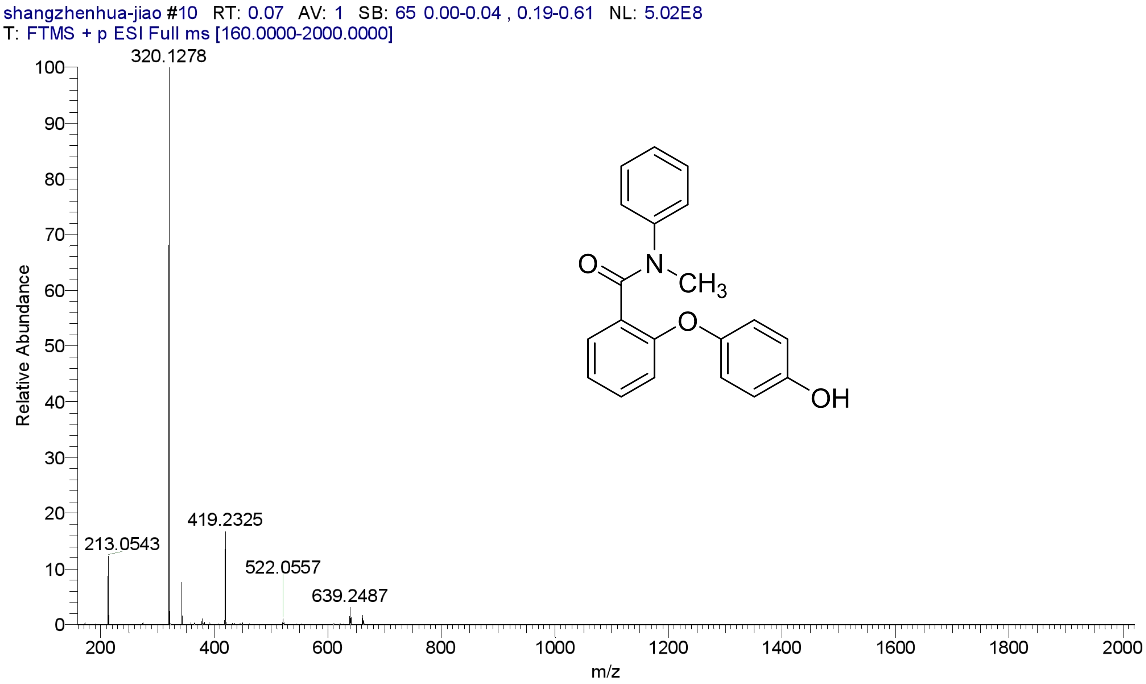

S75: HRMS of **15m**

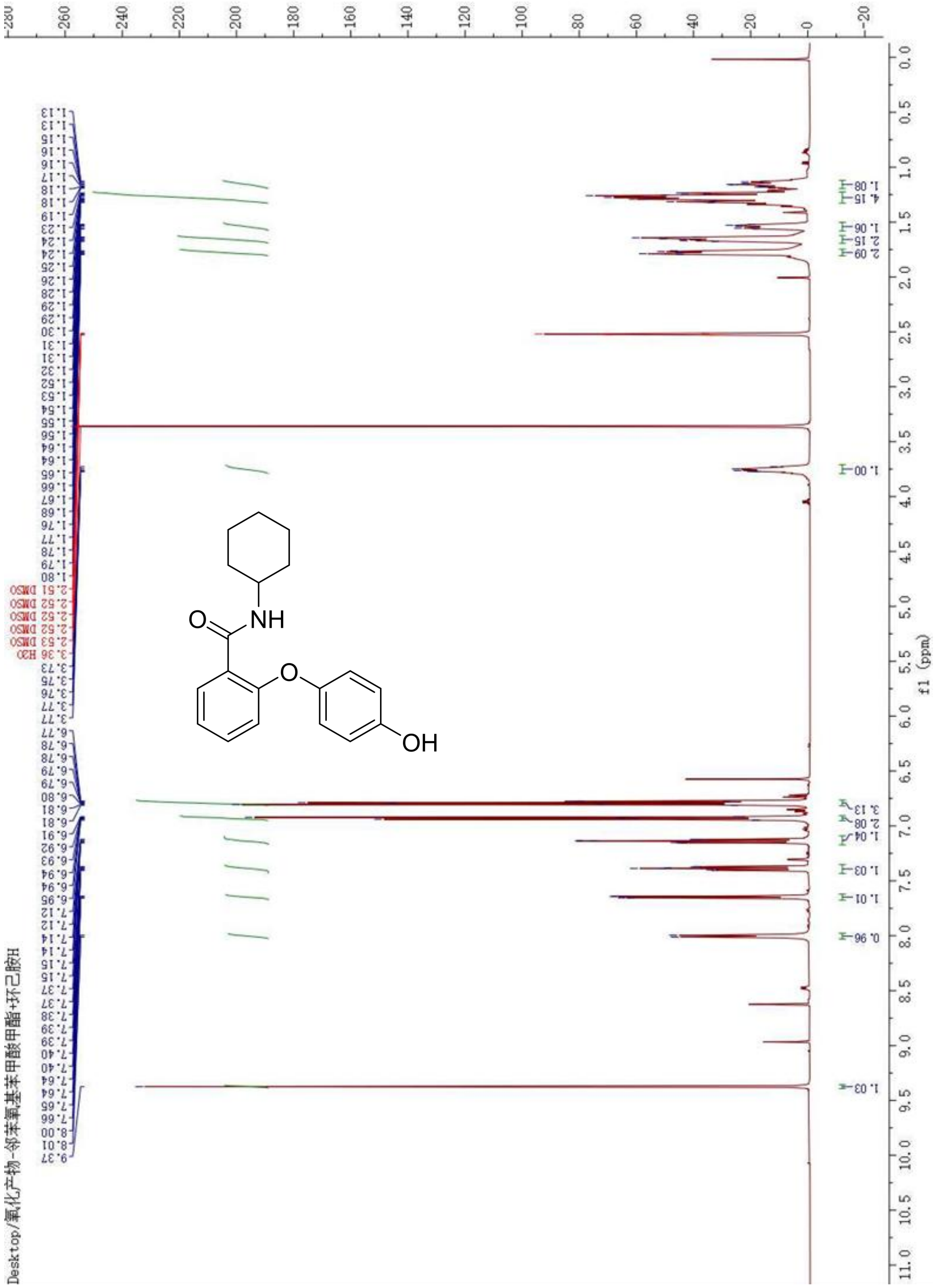

S$_{76}$: ^{1}H NMR of **15n**

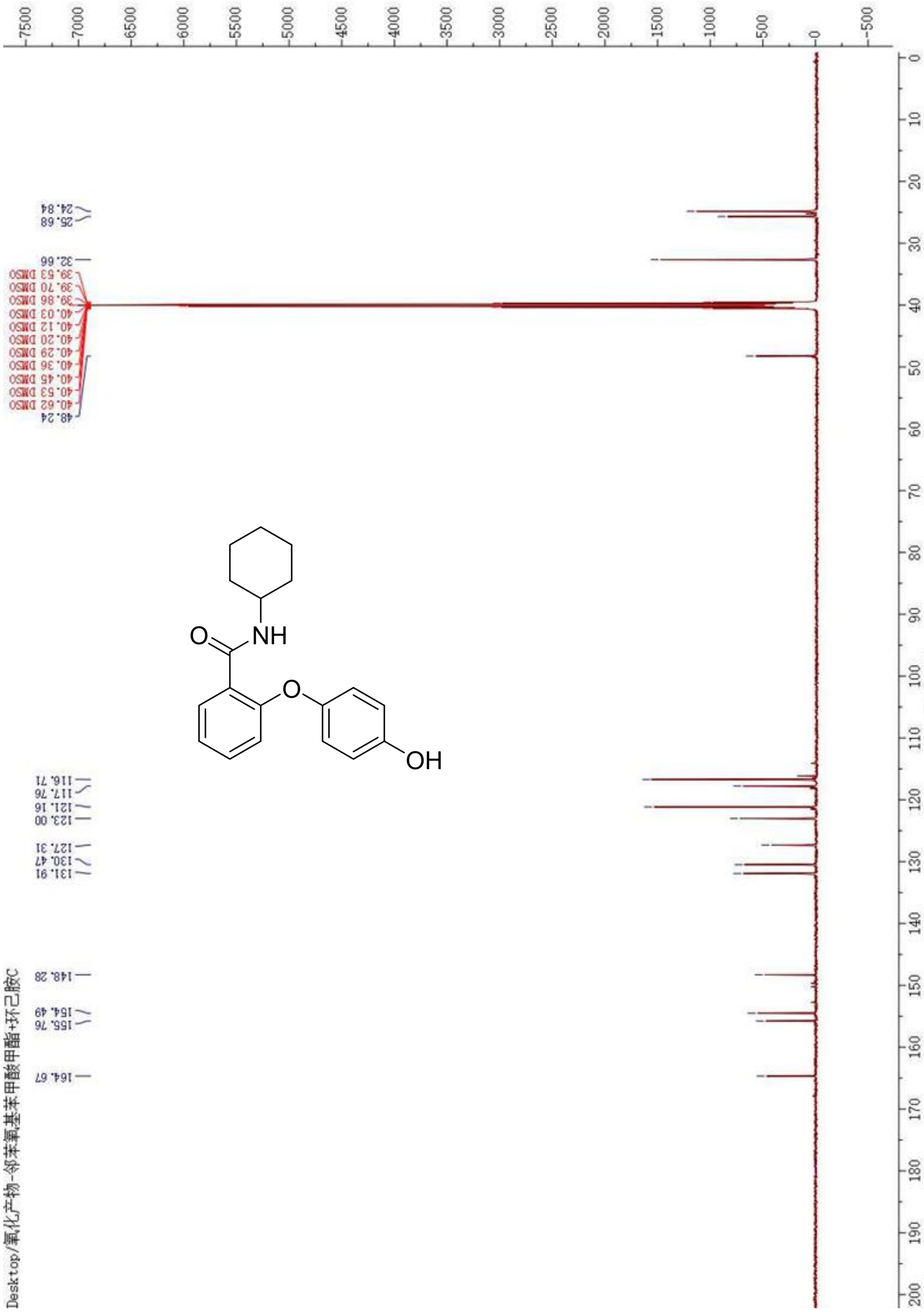

S$_{77}$: ^{13}C-NMR of **15n**

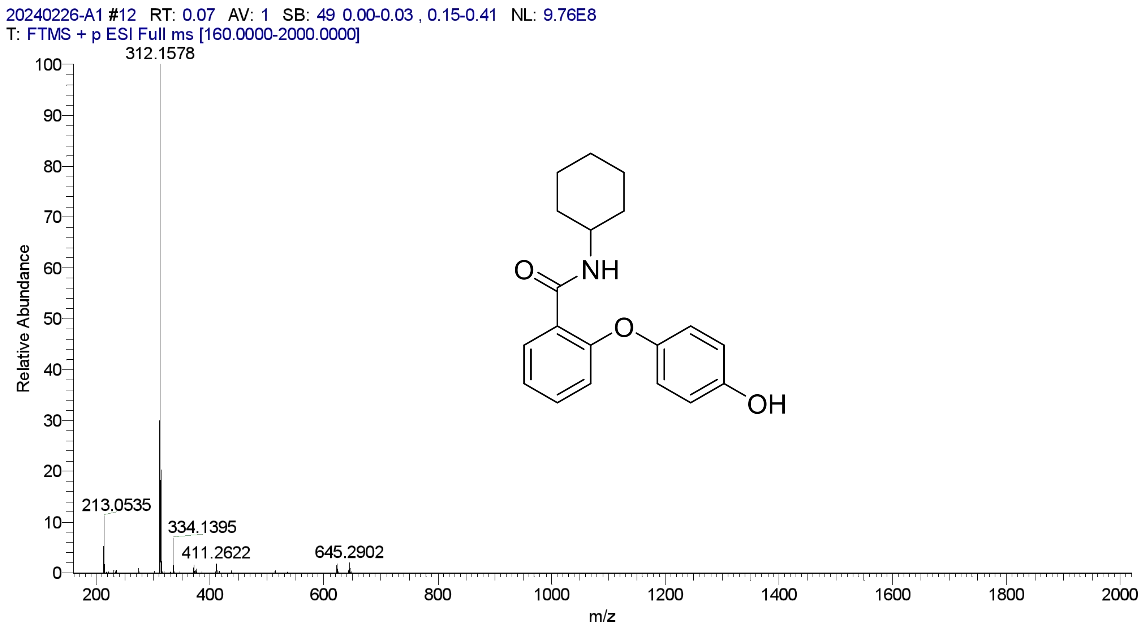

S78: HRMS of **15n**

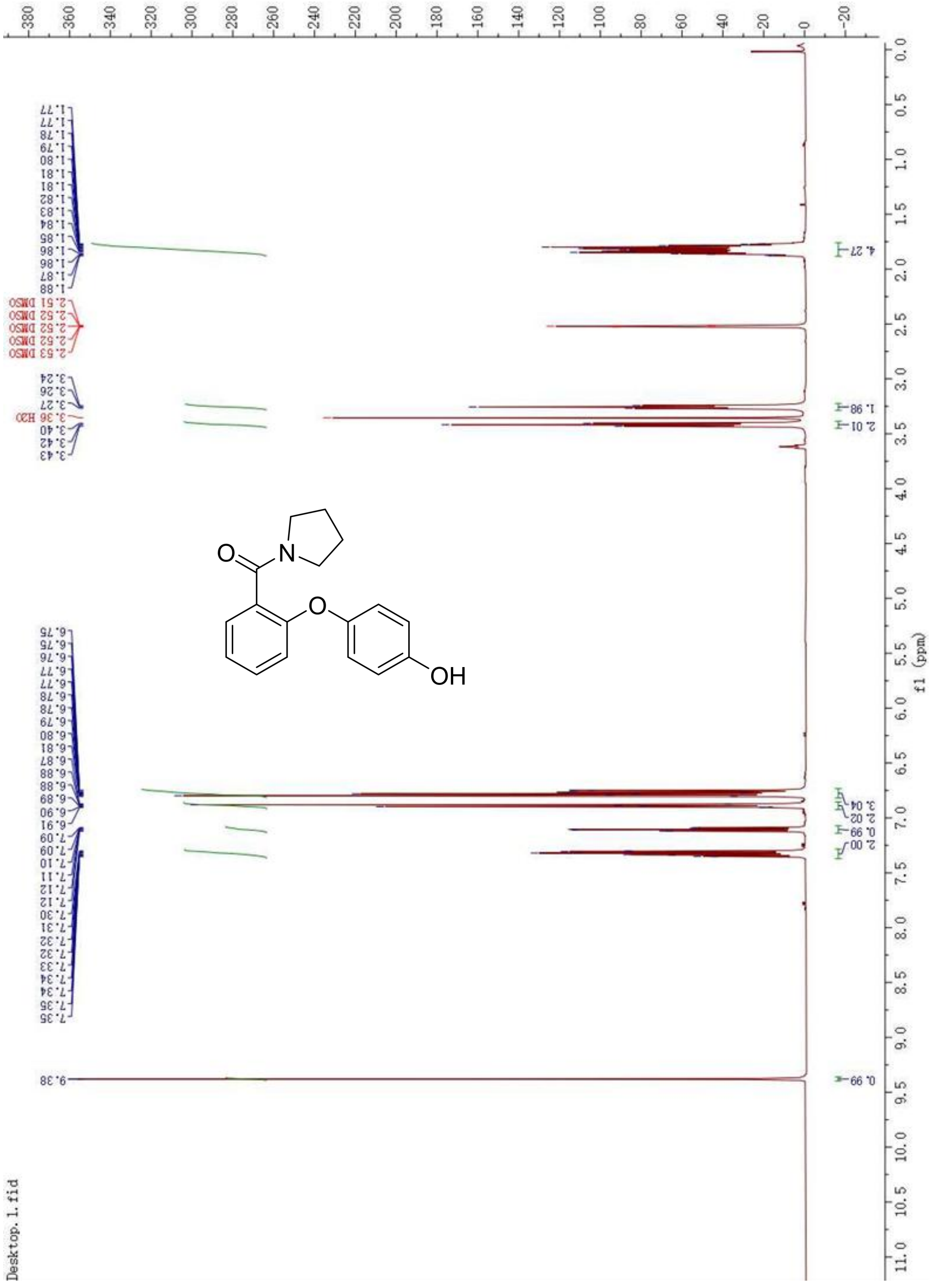

S79: ^{1}H NMR of **15o**

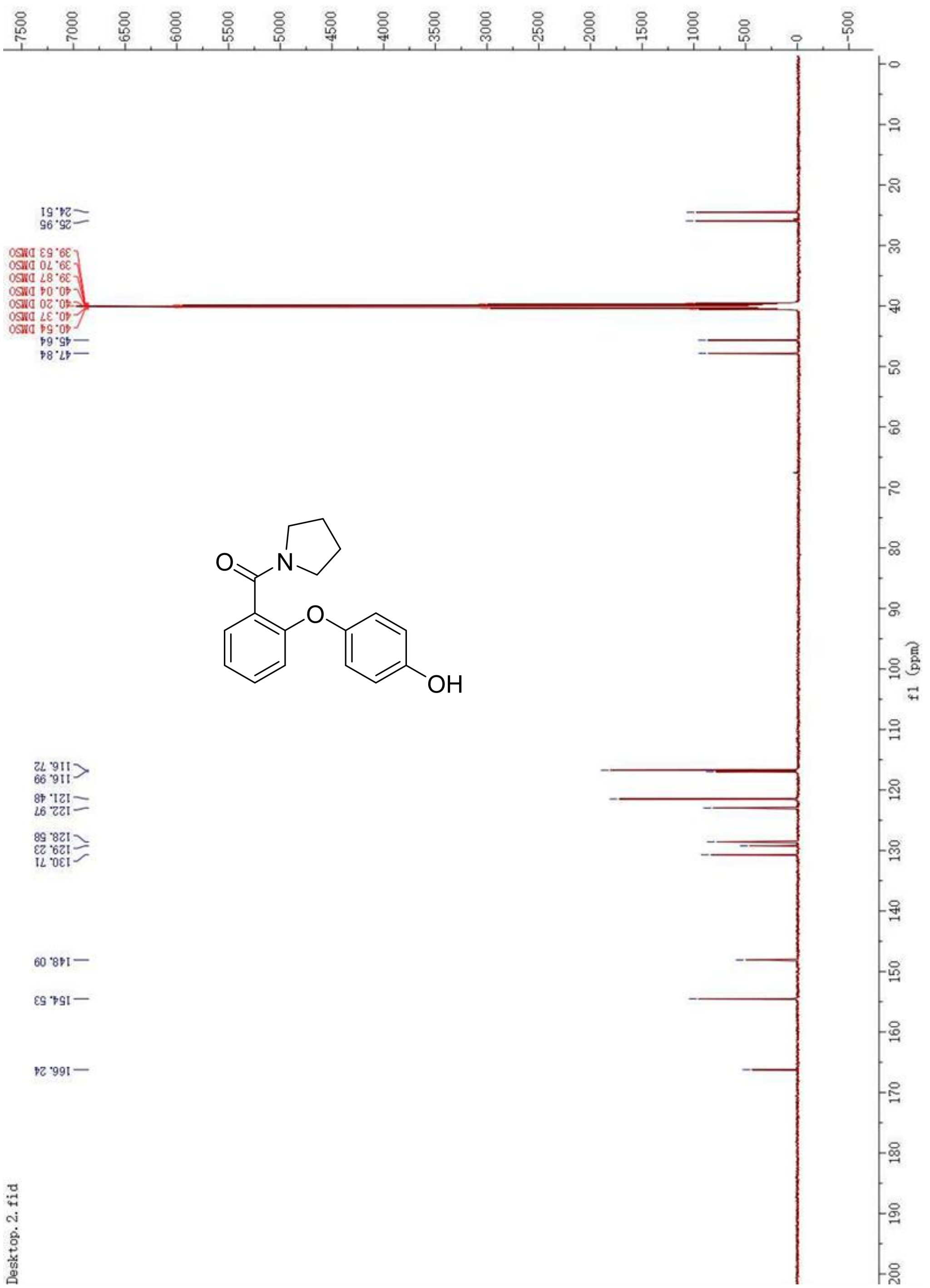

S$_{80}$: ^{13}C-NMR of **15o**

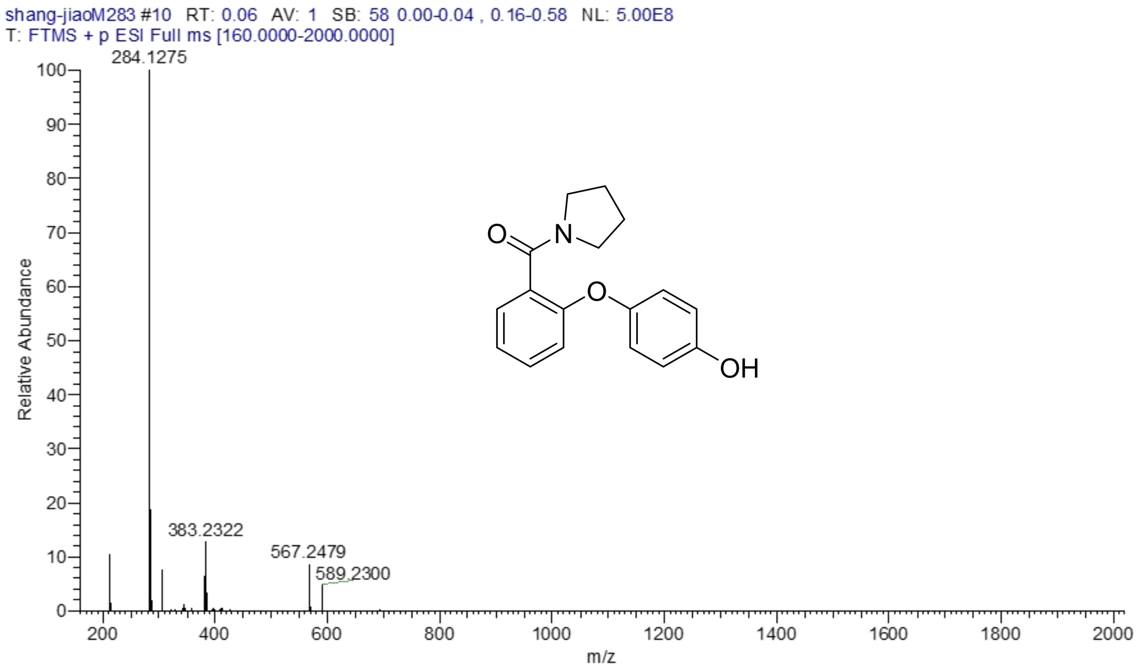

S81: HRMS of **15o**

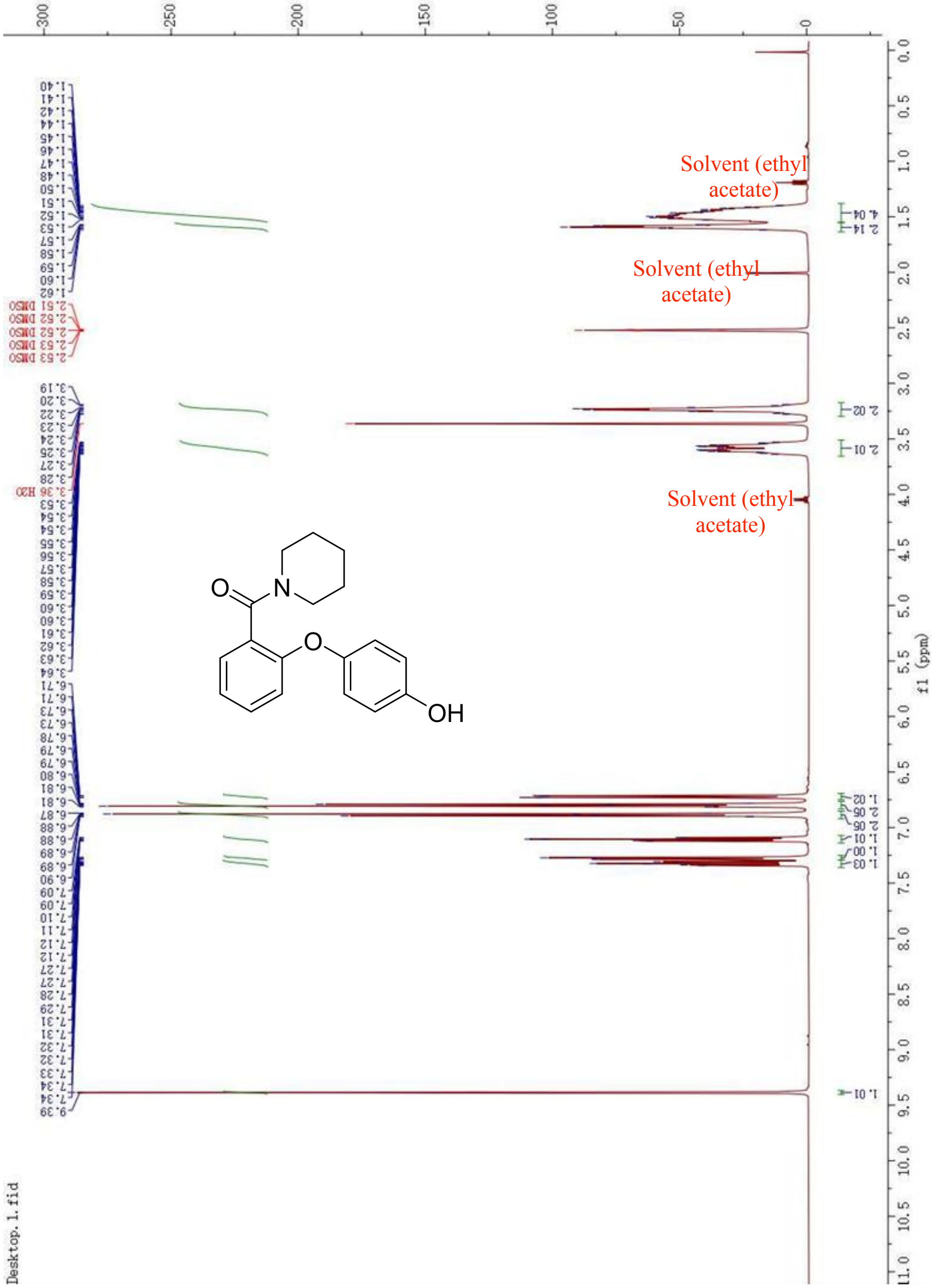

S82: ^{1}H NMR of **15p**

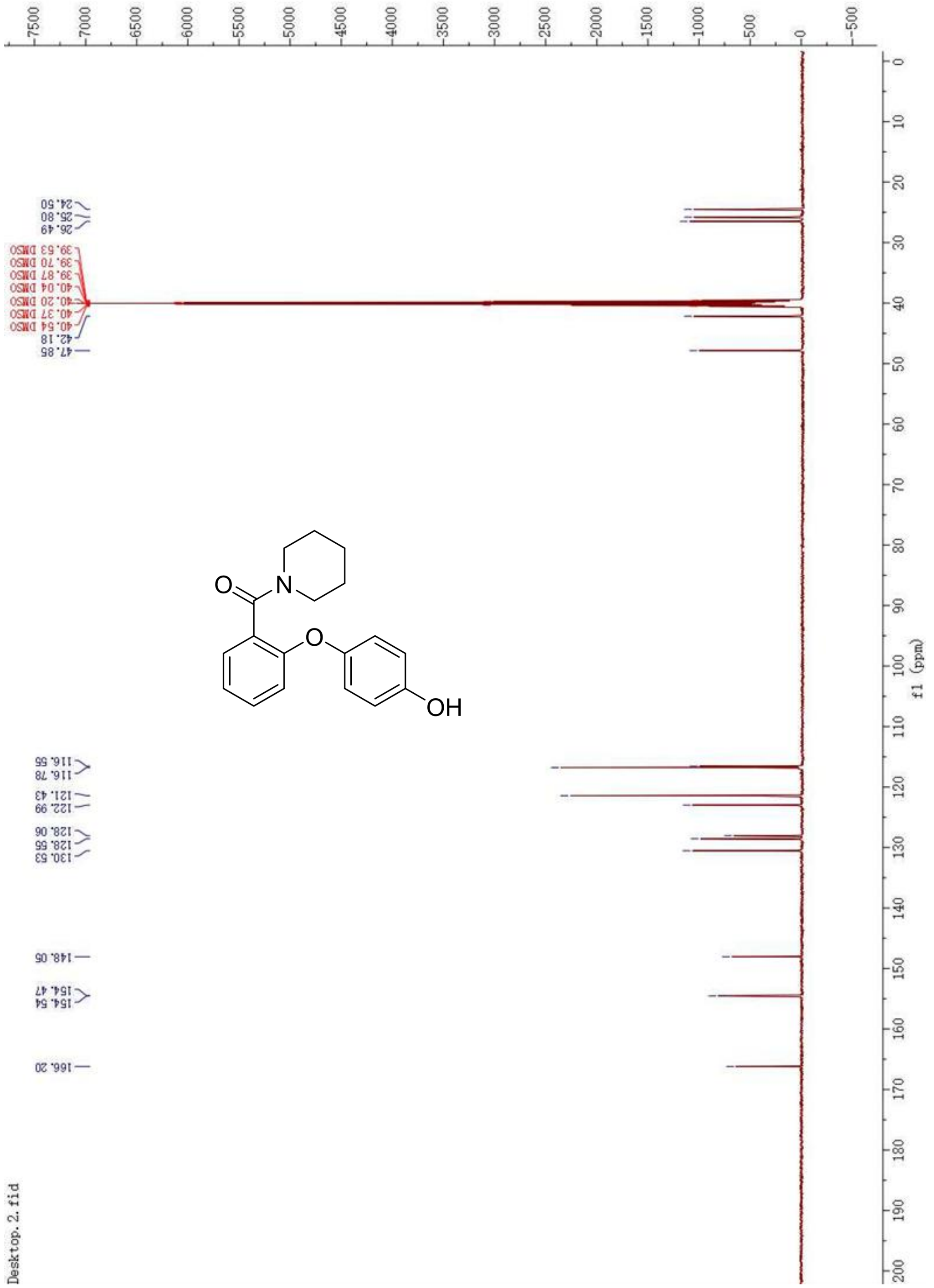

S$_{83}$: ^{13}C-NMR of **15p**

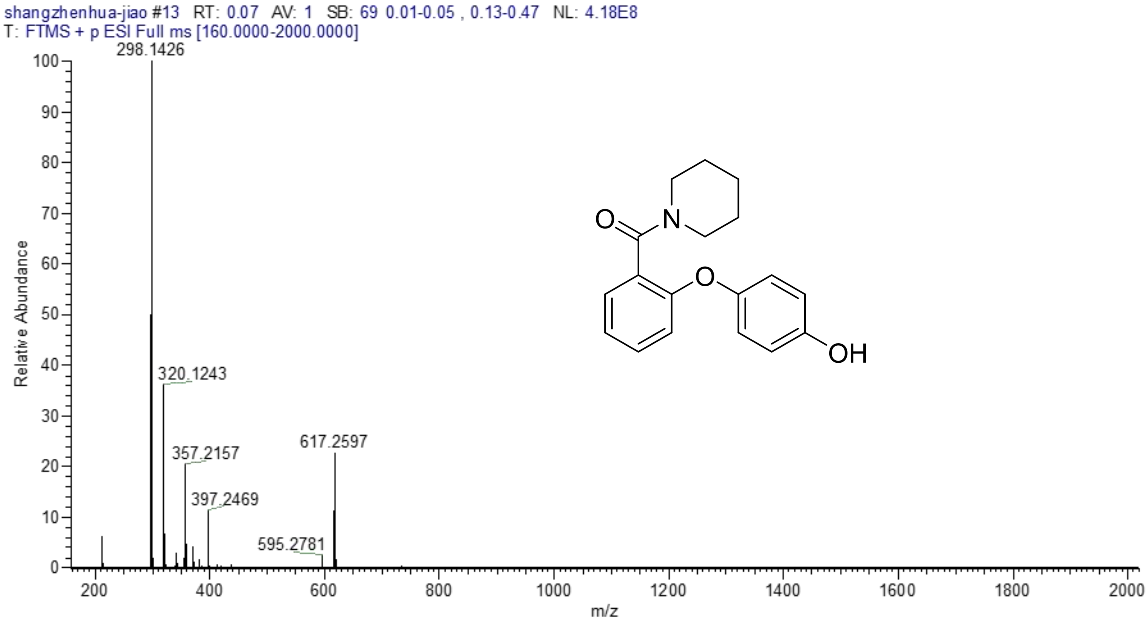

S84: HRMS of **15p**

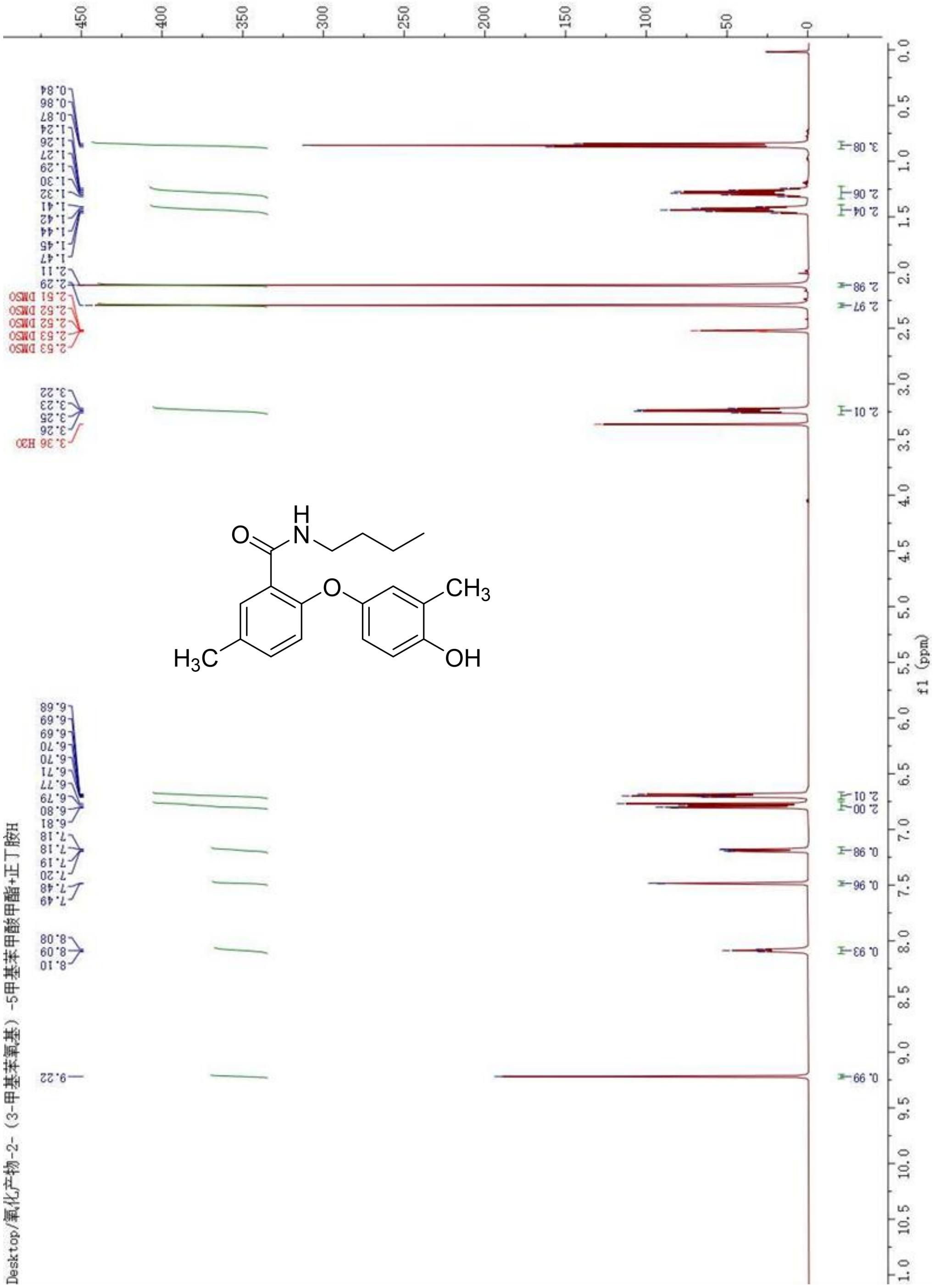

S$_{85}$: ^{1}H NMR of **15q**

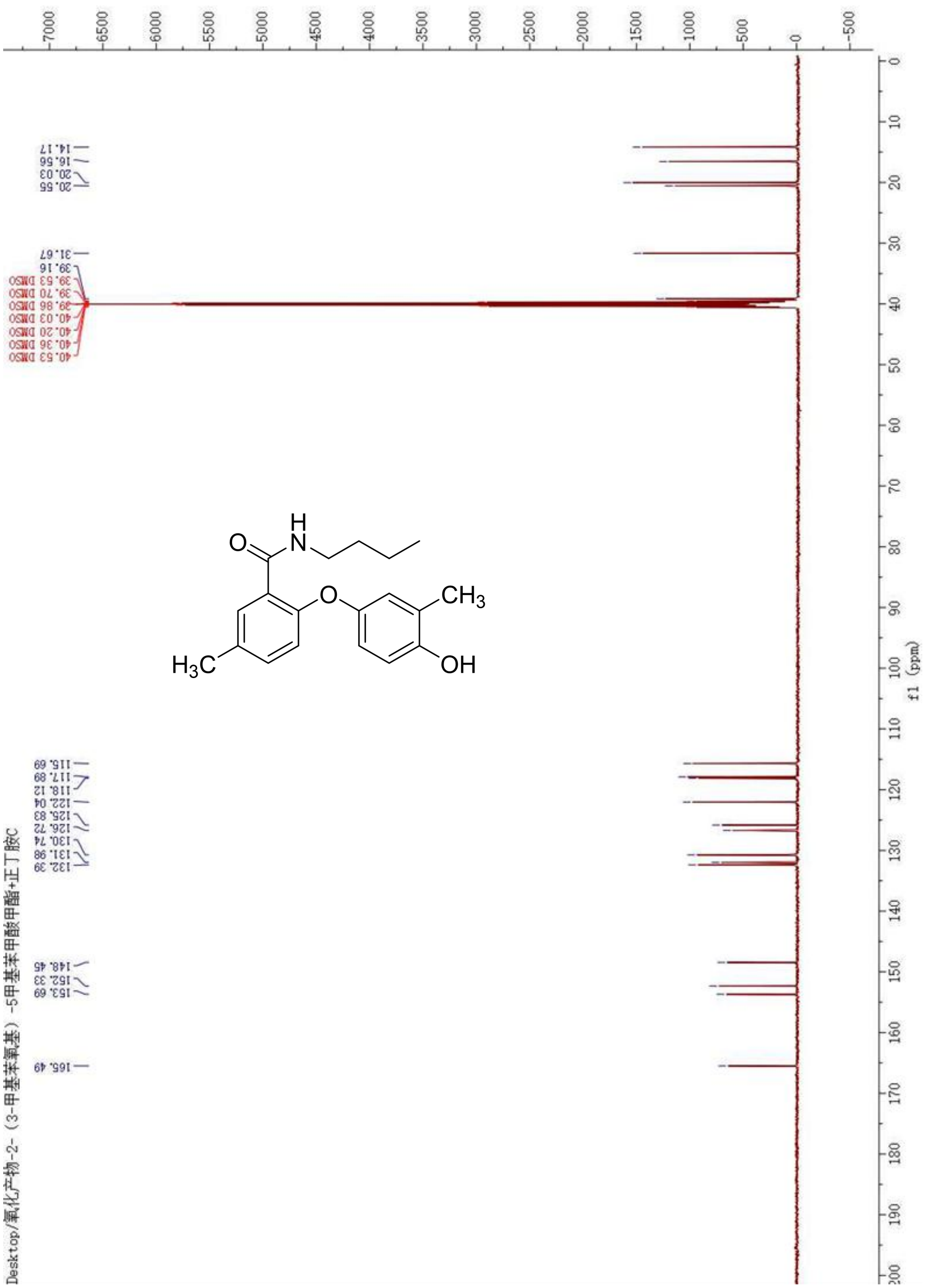

S86: ^{13}C-NMR of **15q**

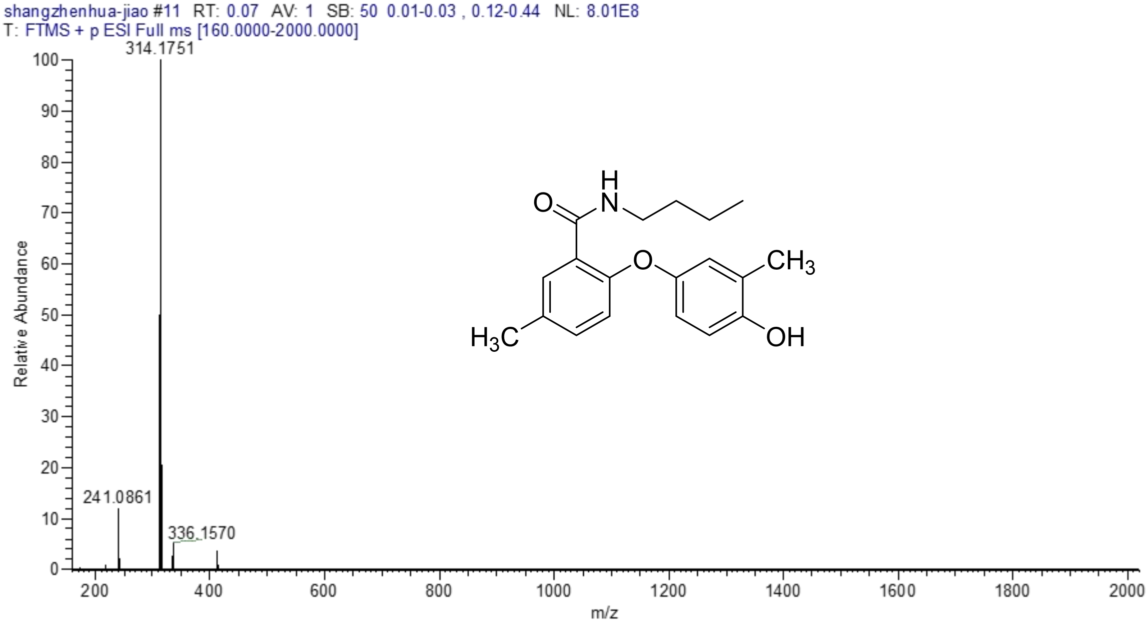

S87: HRMS of **15q**

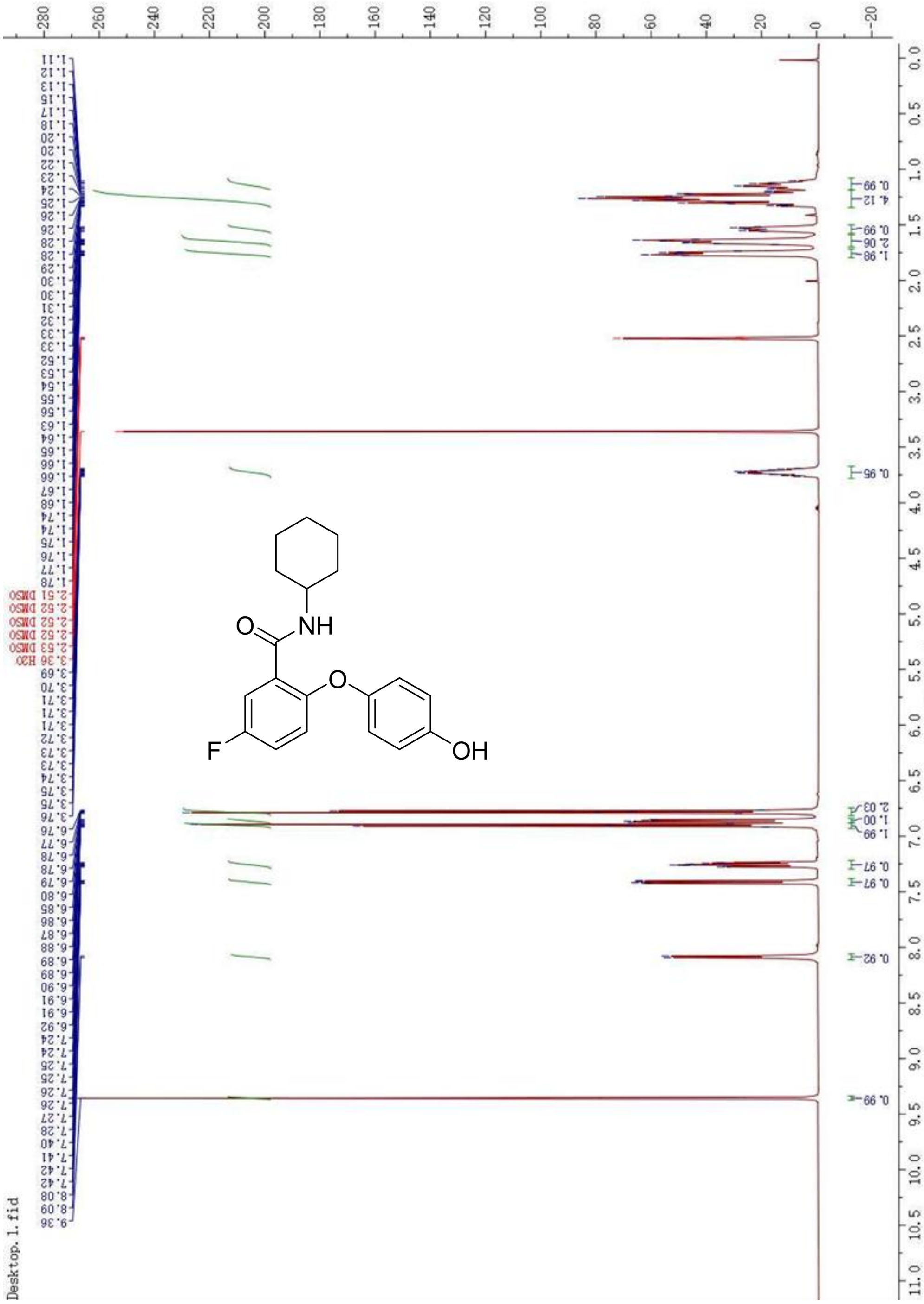

S$_{88}$: ^{1}H NMR of **15r**

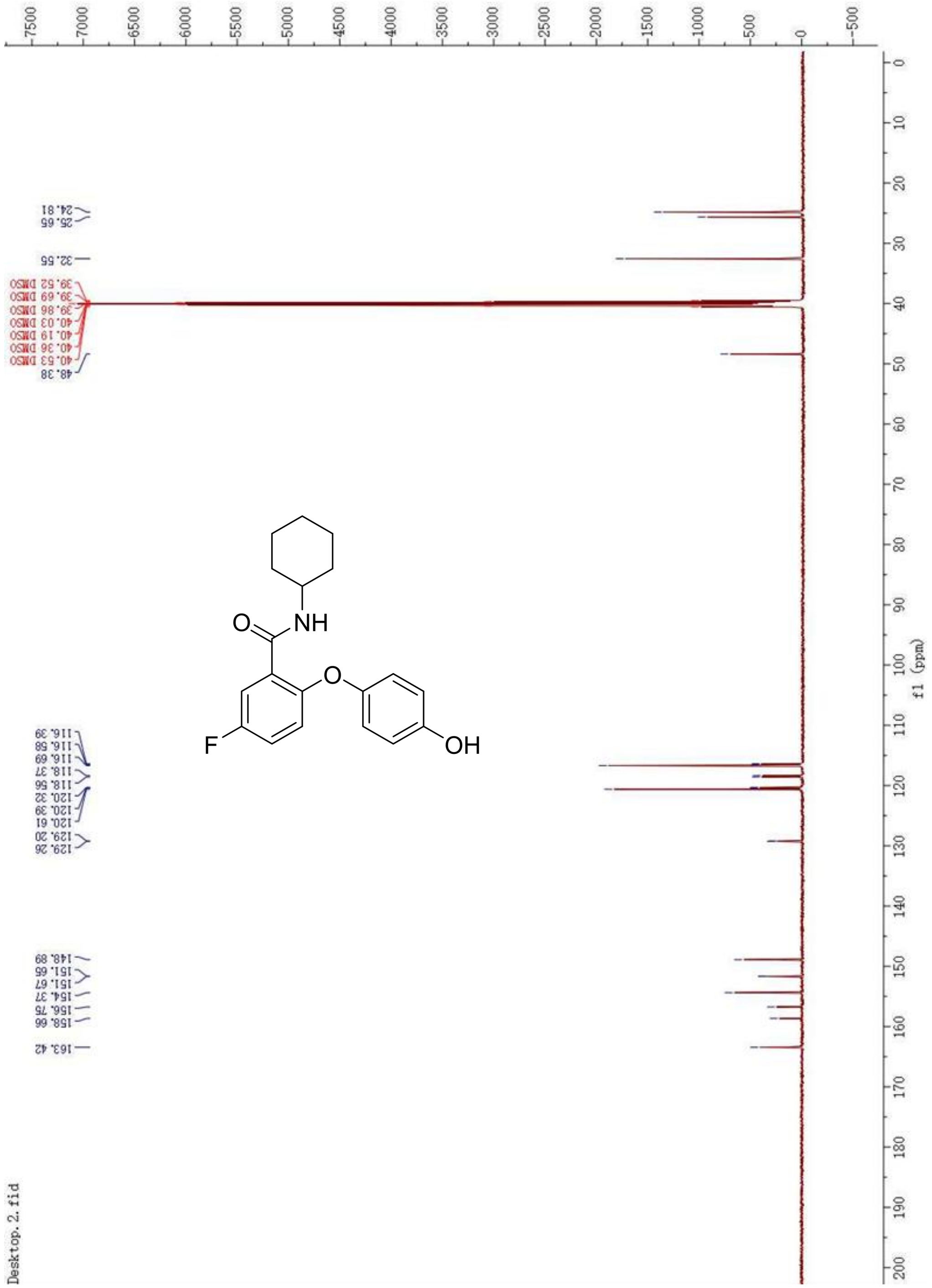

S$_{89}$: ^{13}C-NMR of **15r**

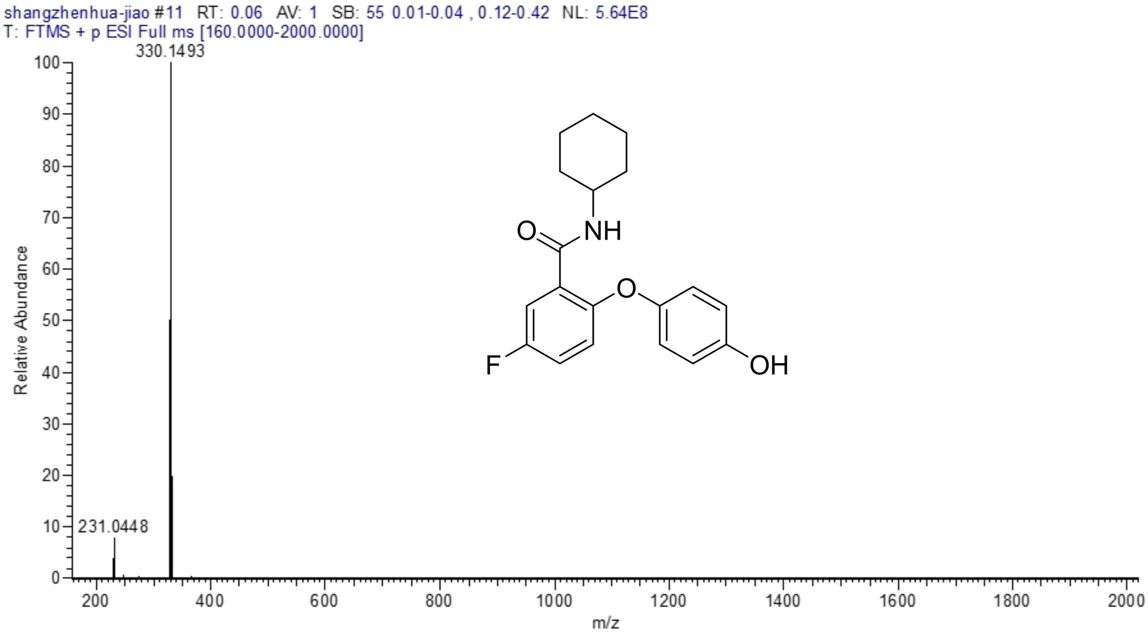

S$_{90}$: HRMS of **15r**
